# Effects of empagliflozin on quality of life and healthcare use and costs in chronic kidney disease: a health economic analysis of the EMPA-KIDNEY trial

**DOI:** 10.1016/j.eclinm.2025.103338

**Published:** 2025-07-08

**Authors:** Junwen Zhou, Claire Williams, Natalie Staplin, Parminder K. Judge, Kaitlin J. Mayne, Nikita Agrawal, Ryoki Arimoto, Jennifer B. Green, David Z.I. Cherney, Katherine R. Tuttle, Jose Leal, Philip Clarke, Jonathan R. Emberson, David Preiss, Christoph Wanner, Martin J. Landray, Colin Baigent, Richard Haynes, William G. Herrington, Borislava Mihaylova, EMPA-KIDNEY Collaborative Group, Colin Baigent, Martin J. Landray, Christoph Wanner, William G. Herrington, Richard Haynes, Jennifer B. Green, Sibylle J. Hauske, Martina Brueckmann, Mark Hopley, Maximillian von-Eynatten, Jyothis George, Susanne Brenner, Alfred K. Cheung, David Preiss, Zhi-Hong Liu, Jing Li, Laiseong Hooi, Wen Liu, Takashi Kadowaki, Masaomi Nangaku, Adeera Levin, David Cherney, Roberto Pontremoli, Aldo P. Maggioni, Natalie Staplin, Jonathan Emberson, Stefan Hantel, Shinya Goto, Rajat Deo, Katherine R. Tuttle, Michael Hill, Parminder Judge, Kaitlin J. Mayne, Sarah Y.A. Ng, Xavier Rossello, Emily Sammons, Doreen Zhu, Peter Sandercock, Rudolf Bilous, Charles Herzog, Paul Whelton, Janet Wittes, Derrick Bennett, Patricia Achiri, Chrissie Ambrose, Cristina Badin, Jill Barton, Richard Brown, Andy Burke, Sebastian Butler, Rejive Dayanandan, Pia Donaldson, Robert Dykas, Lucy Fletcher, Kate Frederick, Hannah Kingston, Mo Gray, Emily Harding, Akiko Hashimoto, Lyn Howie, Susan Hurley, Ryonfa Lee, Nik Luker, Kevin Murphy, Mariko Nakahara, John Nolan, Michelle Nunn, Sorcha Mulligan, Akiko Omata, Sandra Pickworth, YanRu Qiao, Shraddha Shah, Karen Taylor, Alison Timadjer, Monique Willett, Liz Wincott, Qin Yan, Hui Yu, Nichola Jones, Bridget Henderson, Genna Bobby, Louise Bowman, Fang Chen, Robert Clarke, Michelle Goonasekera, William G. Herrington, Waseem Karsan, Marion Mafham, Christina Reith, Mohammed Zayed, Doreen Zhu, Nikita Agrawal, Ryoki Arimoto, Ritva Ellison, Rowan Moys, Will Stevens, Kevin Verdel, Karl Wallendszus, Chris Bowler, Anna Brewer, Andy Measor, Guanguo Cui, Charles Daniels, Angela Field, Bob Goodenough, Ashley Lawson, Youcef Mostefai, Dheeptha Radhakrishnan, Samee Syed, Shuang Xia, Ruth Adewuyi-Dalton, Thomas Arnold, Anne-Marie Beneat, Anoushka Bhatt, Chloe Bird, Andrew Breach, Laura Brown, Mark Caple, Tatyana Chavagnon, Karen Chung, Sarah Clark, Luminita Condurache, Katarzyna Eichstadt, Marta Espino Obrero, Scarlett Forest, Helen French, Nick Goodwin, Andrew Gordon, Joanne Gordon, Cat Guest, Tina Harding, Michal Hozak, Matthew Lacey, David MacLean, Louise Messinger, Stewart Moffat, Martin Radley, Claire Shenton, Sarah Tipper, Jon Tyler, Lesley Weaving, James Wheeler, Elissa Williams, Tim Williams, Hamish Woodhouse, Angela Chamberlain, Jo Chambers, Joanne Davies, Denise Donaldson, Pati Faria-Shayler, Denise Fleming-Brown, Jennifer Ingell, Carol Knott, Anna Liew, Helen Lochhead, Juliette Meek, Isabel Rodriguez-Bachiller, Andrea Wilson, Patrick Zettergren, Meera Mistry, Rach AitSadi, Ian Barton, Alex Baxter, Yonghong Bu, Lukasz Danel, Sonja Grotjahn, Rijo Kurien, Michael Lay, Archie Maskill, Aleksandra Murawska, Rachel Raff, Allen Young, Rebecca Sardell, Vladimir Cejka, Marcela Fajardo-Moser, Christian Hartner, Doris Poehler, Janina Renner, Franziska Scheidemantel, Sharang Ghavampour, Miya Bryant, Anita Hepditch, Cassandra Johnson, Erin Latore, Yolanda Miller, Lauren Price, Merilee Whalen, Ashleigh Wheeler, Sarah Y.A. Ng, Jenny Ingell, Yu An, Yinghua Chen, Peiling Chen, Hao Dai, Hong Du, Fang Feng, Qing Guo, Libo Hou, Wuhanbilige Hundei, Binbin Jin, Yan Li, Jiamin Liu, Xia Song, Yanping Wang, Yanwu Yu, Ning Zhang, Lingshan Zhao, Hui Zhong, Yi Yang, Ying Sun, Cheng Beng Goh, Ye Mun Low, Soon Yi Sor, Farah Hanis Zulkipli, Sarojini Sivanandam, Nurusyifaa’ Nadhirah Mohd Shahfari, Natsuki Arai, Ai Fukasawa, Mizue Furukawa, Keisuke Habuki, Shoko Hayashi, Wakako Isari, Saki Kanegae, Maria Kawai, Reiki Kobayashi, Takako Kuramae, Chika Kuribayashi, Sawako Maeno, Satoshi Masumoto, Tomoko Morisaki, Minoru Oda, Kazue Sawada, Kenta Sugamori, Ayana Tatsuzawa, Aiko Tomita, Kazuyuki Yuasa, Hiroko Inazawa, Amanda Axler, Kerri Gallo, Ester Baldini, Barbara Bartolomei Mecatti, Francesca Bianchini, Martina Ceseri, Laura Cipressa, Gianna Fabbri, Andrea Lorimer, Donata Lucci, Sharang Ghavampour, Anja Knoppe, Tereza Cairns, Hans Schmidt-Gurtler, Hubert Dumann, Sybille Merscher, Margret Patecki, Georg Rainer Schlieper, Anke Torp, Bianca Weber, Maja Zietz, Bernd Hohenstein, Urs Benck, Diliana Draganova, Thomas Weinreich, Lothar Wolf, Jasmine Gaidu, Hanna Reiner, Mandy Visnjic, Daniel Steffl, Marie Breitenfeldt, Annette Kraemer-Guth, Christine Braun, Simone Hagge, Michael Schomig, Stephan Matthias, Dominik Stoffler, Beate Schumacher, Thomas Sitter, Louise Fuessl, Julia Krappe, Jerome Loutan, Volker Vielhauer, Luciano Andriaccio, Magdalena Maurer, Sybille Spies, Bernhard Winkelmann, Martin Dursch, Linda Seifert, Linda Tenbusch, Gudrun Schneckenburger, Tina Geinitz, Kerstin Michalek, Simon Steininger, Julia Mueller, Julia Weinmann-Menke, Simone Boedecker, Wiebke Kaluza-Schilling, Daniel Kraus, Carina Krieger, Margit Schmude, Anne Schreiber, Ewelina Eckrich, Diethelm Tschope, Abdulwahab Arbi, Young Lee-Barkey, Bernd Stratmann, Natalie Prib, Sina Rolfsmeier, Irina Schneider, Lars Rump, Johannes Stegbauer, Christine Pötz, Mara Schemmelmann, Claudia Schmidt, Sinje Landmann, Michael Koch, Sendogan Aker, Annika Küpper, Manuela Martin, Thiemo Pfab, Christian Albert, Michael Haase, Barbara Zander, Claudia Schneider-Danwitz, Wolfgang Seeger, Wolf-Adam Seeger, Britta Zemann, Christoph Stellbrink, Kristin Marx, Ekaterina Stellbrink, Britta Brettschneider, Stephanie Watson, Marion Iselt, Gerhard Klausmann, Inga-Nadine Kummer, Auguste Kutschat, Simone Streitenberger, Matthias Girndt, Silke Markau, Ina Girakossyan, Claudia Hanf, Joachim Beige, Ralph Wendt, Ulrike Schmidt, Birgit Labitzke, Leoni Leistner, Andreas Schneider, Roland Veelken, Claudia Donhauser, Luis Becker, Nexhat Miftari, Ricarda Wolfling, Sarah Morlok, Christian Hugo, Alexander Paliege, Jens Passauer, Julian Stumpf, Annegret Fleischer, Kerstin Haaser, Bernhard Kraemer, Jan Jochims, Bernd Kruger, Claudia Foellinger, Anastassiya Reisler, Frank Strutz, Stefan Haack, Ursula Hohenstatt, Martin Busch, Konstantin Herfurth, Gunter Wolf, Rainer Paul, Andy Steiner, Hermann Haller, Jessica Kaufeld, Jan Menne, Elisabeth Bahlmann-Kroll, Angela Bergner, Kai Schmidt-Ott, Horst Weihprecht, Aydin Er, Florian Sonntag, Elif Turan, Michael Wittmann, Franziska Klauser, Eva Voigt, Julia Gatzschmann, Franziska Thieme, Volker Schettler, Egbert Schulz, Madlen Rohnstock, Elke Schettler, Bernd Schroppel, Rene van Erp, Martin Kachele, Ulla Ludwig, Lena Schulte-Kemna, Waltraud Kmietschak, Elke Preiss, Martina Ruocco, Gunnar Heine, Martin Brzoska, Sebastian Gabel, Christina Büttner, Asma Sabarai, Christina Buttner, Bernhard Banas, Tobias Bergler, Yvonne Ehrl, Franz Putz, Antonia Schuster, Stefanie Kuhn, Torsten Schramm, Stefan Degenhardt, Gerhard Schmidt, Lea Weiland, Ulrike Giebeln-Hudnell, Jan Kielstein, Gabriele Eden, Brigitte Fuchs, Gina Morig, Manuela Winkler, Christina Engel, Harald Darius, Charalampos Kriatselis, Carl-Philipp Roesch, Astrid Maselli, Dominik Alscher, Markus Ketteler, Moritz Schanz, Severin Schricker, Bianka Rettenmaier, Andrea Schwab, Fateme Rahimi, Pablo Pergola, Irene Leal, Melissa Cagle, Anna Romo, Anthony Torres, Natalia Cabrera, Sucharit Joshi, Kulli Barrett, Alexis Africano, Vicki Dodds, Dorleena Gowen, Ashlee Morris, Stacey Perry, Juan Fernandez, Guillermo Jimenez, Ricardo Viera, Kendaling Bruce, Ryan Barrios, Maylin Garcia, Kerelyn Garcia, Iradis Leal, David Tietjen, David Bains, Carlo Castillo, Genielle Brewer, Justin Davis, Natalie Freking, Brittany Golson, Sally Ham, Jesslyn Roesch, Pusadee Suchinda, Shameem Beigh, Usah Lilavivat, Joyce Bilton, Kim Bocchicchia, Heidi Griswold, Jeffrey Turner, Neera Dahl, Aldo Peixoto, Yasemin Kavak, Lauren Liberti, Hari Nair, Nicolas Page, Stephanie Rosenberg, Kathryn Simmons, Tamara Isakova, Rebecca Frazier, Rupal Mehta, Anand Srivastava, Patrick Fox, Jonathan Hecktman, Alexander Hodakowski, Carlos Martinez, Rachel Phillips, Alexis Stevenson, Marija Zimkute, Reed Jaworski, Reem Mustafa, Kyle Jansson, Cassandra Kimber, Jason Stubbs, Ahmad Tuffaha, Sri Yarlagadda, Debbie Griffin, Elisabeth Laundy, Zhuo Tang, Casey Tan, Abigayle Joyce, Radica Alicic, Ann Cooper, Lisa Davis, Ashwini Gore, Rebecca Goldfaden, Leslie Harvill, Lisa Hichkad, Barry Johns, Thomas Jones, Kayla Merritt, Jennifer Sheldon, Jennifer Stanfield, Lindsay Alexander, Kaitlyn Preston, Lindsey Wood, Rajesh Pradhan, Roger DeRaad, Kelli McIntosh, Louis Raymond, Michael Shepperd, Susan McLaughlin, Mary Seifert, Andrew Shepherd, Joseph Aiello, William Durham, Laurie Loudermilk, John Manley, Sabrina Burnette, Stephanie Evans, Tara Johnson, Lance Sloan, Judy Ann Acosta, Stacy Gillham, Katia Sloan, SueAnn Squyres, Michael Rocco, Amret Hawfield, Ben Bagwell, Lauren Richmond, Joseph Soufer, Subha Clarke, Amanda Aliu, Kristine Calabrese, Amanda Davis, Veronica Poma, Tracy Spinola, James Magee, Ricardo Silva, Rushab Choksi, Lorraine Dajani, John Evans, Anil George, Prasanth Krish, Gerard Martins, Mae Sheikh-Ali, David Sutton, Freda Driver, Abraham Hanburry, Laura Hume, Amber Hurst, Matthew Taddeo, Marla Turner, Veronica Yousif, Srinivasan Beddhu, Laith Al-Rabadi, Nikita Abraham, Amalia Caamano, Judy Carle, Victoria Gonce, Kaitlyn Staylor, Na Zhou, Shweta Bansal, Manoj Bhattarai, Kumar Sharma, Subrata Debnath, Aliseiya Garza, Chakradhar Velagapudi, Sergio Rovner, Javier Almeida, Pablo Casares, Verlaine Stewart-Ray, Rene Almaraz, Renata Dayrell, Ana Moncada, Ricardo Pulido, Roxana Rodriquez, Wasim Deeb, Kathryn DeGoursey, Rodel Gloria, Trevor Greene, Robert Miller, Edward Pereira, Miguel Roura, Debbie Domingo, Sasha Dorestin, William Hodge, Cathy Jackson, Deborah Lund, Katrina Taylor, Kenneth Boren, Brittany Cleveland, Sandra Gaiser, Mandeep Sahani, Logan Aldrich, Exodus Edmerson, Edmond Limon, Cole Valletta, Patricia Vasquez, Amanda Harrington, Haley Edwards, Jennifer Green, Christopher Provenzano, Navkiranjot Brar, Heather Henderson, Bellovich Keith, Qur Khai, Quresh Khairullah, Gail Makos, Joel Topf, Sherry Gasko, Rosemarie Henschel, Kaitlin Knapp, Teresa Kozlowski, Paula LaFleur, Ashwathy Varughese, Hui Xue, Patricia Wu, Olga Arechiga, Shan Darbeau, Michael Fechter, Stephanie Martinez, Katherine Klein, Eva Rodriguez, Lenita Hanson, Nyla Cooper, Arelis Madera, Jay Cadorna, Rita Sheridan, Helen Sparks, Bradley Eilerman, Susanne Bodine, Wael Eid, Rebecca Flora, Amber Avery, Cashmere Hardy, Mihaela Biscoveanu, Steven Nagelberg, Tracey Cummins, Frederic Rahbari-Oskoui, Anju Oommen, Zohreh Forghani, Stacie Hitchcock, Darya Hosein, Diane Watkins, Minesh Patel, Anthony Lambert, Elizabeth Newman, Autumn Wood, Tammy Ross, Stephany Topping, Jeffrey Mulhern, Lorna Murphy, Ann Vasseur, Gregory Greenwood, Alexander Hadley, Denise Laurienti, Christopher Marshall, Nicholas McLean, Scott Satko, Brandy Caudill, Jacob Maris, Janice Rogers, Cindy Vanhoy, George Thomas, Georges Nakhoul, John O'Toole, Jonathan Taliercio, Leslie Cooperman, Marina Markovic, Barbara Tucky, Devasmita Dev, Alia Hasan, Hima Yalamanchili, Namita Jain, Lesley McNeil, Eric Wines, Jean Park, Adline Ghazi, Mia Hamm, Tejas Patel, Amy Mottl, Emily Chang, Vimal Derebail, Emmie Cole, Anne Froment, Sara Kelley, Jordan Osmond Foster, Vahid Mahabadi, Golriz Jafari, Anita Kamarzarian, Wendy Arriaga, Daisy Arteaga, Rosario Machicado, Genesis Naverrete, Prashant Kumar, Imran Nazeer, Karina Urquia, Tammi Glider, Vickie Jones, Savannah Rucker, Jennifer Wiley, Tammy Rider, Rahul Pandey, Jesus Arroyo, Harish Pariani, Mohammad Ahmad, Shahin Mozaffari, Erika Perez, Andres Miranda, Matthew Budoff, Sion Roy, Divya Birudaraju, Ahmed Ghanem, Sajad Hamal, Stephen Aronoff, Elisa Joye Petr, Richard Sachson, Jaime Wiebel, Sana Akram, Laurie Jones, Curtis Knight, Maurie Tarlac, Idara Ukpong, Kim Quiroga, Shahbaz Ahmed, Harold Szerlip, Akinwande Akinfolarin, Ankit Mehta, Shana Camp, Cindy Castro, Zanaida Cooper, Jessica Terry, Ahmed Awad, Bhavya Kothapalli, Ryan Lustig, Serine Alfaress, Hyder Jasim, Mary Parrigon, Dennis Karounos, Sadiq Ahmed, Maggie Berry, Ruth Oremus, Carlos Hernandez-Cassis, Elias Ugwu, Nazia Junejo, Nancy Suazo, Todd Clark, Rosalinda Cruz, Mark Segal, Amir Kazory, Sherry Brown, Tristan Daniels, Sofia Dayi, Renee Hogan, Kathy McCray, Jennifer Stickley, Mahboob Rahman, Mirela Dobre, Lavinia Negrea, Aparna Padiyar, Nishigandha Pradhan, Arash Rashidi, Nagaraju Sarabu, Vicki Donley, Tricia Young, Elizabeth DeCaro, Godson Oguchi, Judepatricks Onyema, Kahla Damianik, Jack Dienes, Judith Plummer-Morgan, Marilyn Roman, Mauver Skipper, Stacey-Ann Villaruel, Krystle Williams, Svetlana Shilo, Numaliz Chokr, Danny Sugimoto, Jeffrey Dugas, Ismeal Ahmed, Jamie Bhairoo, Dolores Rijos, Huzaifa Salim, Kaleena Urquidi, Madita Gavrila, Kathryn Lafferty, Ria Rabara, Sally Ruse, Maria Weetman, James Bushnell, Albert Power, Alison Jenkins, Stefanie Jones, Amanda Scott, Cath Byrne, Mark Jesky, Alison Cowley, Emma McHaffie, Holly Waterfall, Neha Bhalla, Jo Taylor, Laura Bough, Thomas Phillips, Barbara Winter-Goodwin, Keegan Lee, Sui Phin Kon, Iain MacDougall, Eirini Lioudaki, Sapna Shah, Claire Sharpe, Francisco Aguilar, Abegail Hernandez Pena, Conception Pugay, Amelia Te, Tony Johny, Philip Francisco, Hugh Finn, Wasim Hanif, Samiul Mostafa, Alice Aitken, Katharine Draxlbauer, Evelina Grobovaite, Jennifer Kearney, Theresa McCarthy, Faye Moore, Christianah Morakinyo, Sephora Thorpe, Giorgio Gentile, Duncan Browne, Palanichamy Chellamuthu, Tabinda Dugal, Terri Chant, Laura Jones, Emily Laity, Megan Miners, James Muir, Elizabeth Swanson, Andrew Frankel, James Tomlinson, Marlon Alegata, Rashid Almasarwah, Anthoula Apostolidi, Maria Vourvou, Thomas Walters, Maarten Taal, Hari Dukka, Nitin Kolhe, Carly McDonald, Kelly White, Shiva Ugni, Smita Gunda, Rotimi Oluyombo, Vicki Brindle, Ping Coutts, Tracy Fuller, Evelyn Nadar, Suresh Ramadoss, Nichola Motherwell, Susannah Pajak, Louise Tonks, Mandy Beekes, Sunil Bhandari, Richard Bodington, Adil Hazara, Dominic Fellowes, Christopher Wong, Christopher Goldsmith, Sherald Barnes, Ann Bennett, Claire Burston, Samantha Hope, Nicola Hunt, Lini Kurian, Richard Fish, Daniela Farrugia, Judy Lee, Emma Sadler, Hannah Turner, Christopher Hill, Henry Brown, Agnes Masengu, Peter Maxwell, Nina Bleakley, Hugh Murtagh, William Petchey, Vivian Yiu, Joanne Kellett, Angharad Williams, Veronica Mendez Morro, Helen Clarke, Victoria Carnall, Sarah Benyon, Caroline Blake, Stephanie Estcourt, Jane Piper, Gigee Joseph, Neal Morgan, Carolyn Hutchinson, Teresa McKinley, Alastair Woodman, Judi Graham, Niall Leonard, John Smyth, Vicki Adell, Samantha Hagan, Ben Caplin, Amin Oomatia, Eleanor Damian, Toluleyi Sobande, Phil Gardiner, Tim Doulton, Michael Delaney, Mahmoud Montasser, Jenny Hansen, David Loader, Angela Moon, Frances Morris, Smeeta Sinha, Chukwuma Chukwu, Amy Hudson, Diane Campbell, Melanie Kershaw, Stephanie Whittaker, Katarzyna Adeniji, Ayesha Irtiza-Ali, Farid Ghalli, Heba Nosseir, Allison Leslie, Kate Trivedi, Donald Fraser, Mohammad Alhadj Ali, Sian Griffin, Farah Latif, Justyna Witczak, Alexa Wonnacott, Lynda Jeffers, Yvette Webley, Paul Phelan, Eve Miller-Hodges, Ailsa Geddes, Margaret Glenwright, Amy Hunter, Thomas Pickett, Jim Moriarty, Linda Hill, Amanda Tyler, Waqar Ayub, Gail Evans, Sue Hewins, Davina Hewitt, Kerry Read, Samira Bell, Leanne Cosgrove, Rachel Craik, Shona Murray, Nitin Bhandary, Holly Coles, Rashmi Easow, Maya Joseph, Deepa Thapa, Arif Khwaja, Yvonne Jackson, Angeline Mbuyisa, Rachel Sellars, Sadaf Younis, Kimiko Chapman, Nihil Chitalia, Cynthia Mohandas, Anca Gherman, Charlotte Kamundi, Olumide Olufuwa, Ryan Coe, Kieran McCafferty, Adedolapo Adeleke, Cara Healy, Damini Jeyarajah, Edward Kinsella-Perks, Richard Smith, Brian Camilleri, Carol Buckman, Jenny Finch, Vanessa Rivers, Andrew Connor, Sheila Carr, Lisa Shainberg, Andrew Lewington, Richard Baker, Suzannah Dorey, Kay Tobin, Rosalyn Wheatley, Debasish Banerjee, Richard Hull, Sharirose Abat, Riny Paul, Mahzuz Karim, Zay Htet, Saad Tufail, Ravi Varma, Karen Convery, Deirdre Fottrell-Gould, Lisa Hudig, Emily Tropman, Jane Platt, Thahir Abdul-Samad, Anne Grace, Marie Phipps, Gemma Highway, Rebecca Suckling, Subash Somalanka, Bhrigu Sood, Pauline Swift, Sarah Acheampong, Kwame Ansu, Martia Augustin, Anna Sampson, Lynn Vinall, Kim Wren, Shamila Wanninayake, Nicholas Wooding, Heather Edwards, Lydia Owen, Stephanie Bolton, Marion Carson, Michael Matthews, Nigel Brunskill, Jorge Jesus-Silva, Alex Howson, Mary Quashie-Akponeware, April Maria Murillo, Hilary Tindall, Chidambaram Nethaji, Helen Eldon, Rajan Patel, Patrick Mark, Alastair Rankin, Michael Sullivan, Kirsty Forsyth, Rowan McDougall, Tanaji Dasgupta, Louisa Davies, Maggie Ryder, Suzannah Pegler, Philip Grimmer, Clare Macdonald, Mary Webster, Newcastle Newcastle, Timothy Ellam, Edwin Wong, Christine Meshykhi, Andrea Webster, Peter Wilson, Enric Vilar, Jocelyn Berdeprado, Eunice Doctolero, Lily Wilkinson, Frank McCarroll, Hesham Ammar, Ying Kuan, Conor Moran, Girish Shivashankar, Ryan Campbell, Deborah Glowski, Paula McDermott, Amar Ali, Zuber Patel, Christine Bond, Gillian Whalley, Haitao Zhang, Liu Yang, Lihua Zhang, Tingting Kan, Ling Zhu, Jinghong Zhao, Weiping Hou, Jing Wu, Hong Cheng, Weijing Bian, Zhirui Zhao, Fengmin Shao, Huixia Cao, Xiaojing Jiao, Peiyuan Niu, Jianying Niu, Yu Chen, Lihong Zhang, Shenglang Zhu, Haiyan Lin, Shaopeng Yao, Jiehui Chen, Ying Jiang, Ying Hu, Huaying Xiao, Fuye Yang, Xinzhou Zhang, Baochun Guo, Qiu Jin, Lixia Liu, Xiangcheng Xiao, Yanyun Xie, Ting Meng, Chuanwen Xu, Jie Huang, Yanmei Xu, Weixin Kong, Xiaoliang Wang, Qianpan Liu, Xueying Wang, Ming Gao, Xiumei Hu, Ying Lu, Li Wang, Kun Peng, Wei Wang, Qiuhong Gong, Jianfang Cai, Xiaojue Li, Xuejiao Liu, Shuhan Zhou, Hong Liu, Yao Weng, Shuai Tang, Yao Yao, Shi Zhao, Chen Cheng, Wei Wei, Na Li, Sadanah Aqashiah Mazlan, Alia Zubaidah Bahtar, Elliyyin Katiman, Noraini Othman, Lily Mushahar, Nurdiana Mazlan, Nur Sharafina Safiee, Sarasa Ramasamy, Hin Seng Wong, Hajar Ahmad Rosdi, Esther Zhao Zhi Tan, Ju Fan Tay, Kok Seng Teng, Hasnah Yahaya, Wen Jiun Liu, Lik Wee Ee, Kenneth Kay Leong Khoo, Yuana Mohd Yusoff, Fariz Safhan Mohamad Nor, Mohd Kamil Ahmad, Mohd Ramli Seman, Clare Hui Hong Tan, Laura Lui Sian Ngu, Jaime Yoke May Chan, Javelin Peji, Chek Loong Loh, Yee Yan Lee, Sridhar Ramanaidu, Kah Mean Thong, Yik Hong Wong, Suria Junus, Chen Hua Ching, Mohammad Faisal Asmee, Ku Ruziana Ku Md Razi, Chun Leong Low, Christopher Sze Bing Sim, Zhang Duan Tham, Noor Kamila Abdullah, Tai Meng Chen, Yong Chieh Chan, Eason Chang, Huan Yean Kang, Kai Quan Lee, Sue Ann Lee, Aik Kheng Lee, Jeevika Vinathan, Chyi Shyang Tan, Rizna Abdul Cader, Ruslinda Mustafar, Lydia Kamaruzaman, Rozita Mohd, Rahimah Ismail, Chong Men Leong, Chee Koon Low, Liang Wei Wong, Yik Shen Lim, Norlezah Adnan, Sabariah Ibrahim, Mohamad Zaimi Abdul Wahab, Sunita Bavanandan, Yik Shen Lim, Wan Hazlina Wan Mohamad, Siti Munirah Jaafar, Nur Ashykeen Mohd Fauzi, Aziee Sudin, Soo Kun Lim, Chye Chung Gan, Albert Hing, Wan Ahmad Faizal Alaidin Razali, Yew Fong Liew, Chelsia Bao Tyng Chan, Mei Chih Cheng, Yu Chen Ong, Loke Meng Ong, Farah Amalina Mohamed Affandi, Korina Rahmat, Ban Chai Peng, Masayu Amat, Nuzaimin Hadafi Ahmad, Doo Yee Mah, Yi Loon Tye, Zaid Azhari, Siti Nabilah Mohamad Zaini, Mohd Aidil Musa, Nur Nadzifah Hanim Zainal Abidin, Zher Lin Go, Norazinizah Ahmad Miswan, Rafizanur Ramli, Nor Aziah Ahmad, Bak Leong Goh, Nurul Izah Ahmad, Fairol Huda Ibrahim, Tze Jian Ng, Malini Shanmuganathan, Li Lian Tay, Zaiha Harun, Salmi Ramli, Nurul 'Ain Yusof, Rossenizal Abd Rahman, Muhammad Iqbal Abdul Hafidz, Nur Hidayati Mohd Sharif, Irda Yasmoon Awang, Eitaro Nakashima, Rui Imamine, Makiko Minatoguchi, Yukari Miura, Miduki Nakaoka, Yoshiki Suzuki, Hitomi Yoshikawa, Koki Shin, Kanae Fujita, Misuzu Iwasa, Haruka Sasajima, Airi Sato, Yoshiyuki Hamamoto, Yuki Fujita, Takuya Haraguchi, Takanori Hyo, Kiyohiro Izumi, Toshiyuki Komiya, Sodai Kubota, Takeshi Kurose, Hitoshi Kuwata, Susumu Nakatani, Kaori Oishi, Saki Okamoto, Kaori Okamura, Jun Takeoka, Nagaaki Tanaka, Katsuya Tanigaki, Naohiro Toda, Koin Watanabe, Hiromi Komori, Rika Kumuji, Asako Takesada, Aya Tanaka, Shoichi Maruyama, Tomonori Hasegawa, Akiko Ishiguro, Takuji Ishimoto, Kazuhiro Ito, Yutaka Kamimura, Noritoshi Kato, Sawako Kato, Hiroshi Kojima, Tomoki Kosugi, Kayaho Maeda, Masasi Mizuno, Shoji Saito, Hitomi Sato, Yuka Sato, Yasuhiro Suzuki, Akihito Tanaka, Yoshinari Yasuda, Fujiko Hasegawa, Maiko Hayashi, Shizuka Higashi, Kaho Shimamura, Momoko Sumi, Kazuki Tajima, Chimaki Unekawa, Kana Wakayama, Yukiko Wakita, Takatoshi Otani, Ayako Imai, Sayaka Kawashima, Eri Kogure, Tomoe Sato, Misato Takezawa, Shinya Yoshida, Hideo Araki, Yuko Katsuda, Masahiro Konishi, Takahiro Matsunaga, Masashi Oe, Kunihiro Ogane, Masato Sakai, Tomoko Takahashi, Takahiro Yamano, Takuya Yokoyama, Hitomi Ito, Masayo Katayama, Emi Kuroda, Toru Ikeda, Takuma Kojo, Etsuo Yoshidome, Rieko Mizumachi, Akane Yamamoto, Narihisa Yamasaki, Yoshihiko Yamasaki, Jun Wada, Jun Eguchi, Chigusa Higuchi, Akihiro Katayama, Masaru Kinomura, Masashi Kitagawa, Shinji Kitamura, Satoshi Miyamoto, Hiroshi Morinaga, Atsuko Nakatsuka, Ichiro Nojima, Kenichi Shikata, Hitoshi Sugiyama, Katsuyuki Tanabe, Kenji Tsuji, Haruhito Uchida, Mayu Watanabe, Chie Hashimoto, Takahiro Kato, Sayaka Yamamoto, Takehiko Wada, Masafumi Fukagawa, Naoto Hamano, Masahiro Koizumi, Hirotaka Komaba, Yosuke Nakagawa, Michiyo Iwamoto, Kosuke Masutani, Akane Katanosaka, Mayu Kiyota, Hikari Uchi, Yuka Ueda, Sonoka Yamamoto, Hajime Nagasu, Seiji Itano, Tsukasa Iwakura, Hiroyuki Kadoya, Eiichiro Kanda, Naoki Kashihara, Kengo Kidokoro, Megumi Kondo, Tamaki Sasaki, Minoru Satoh, Atsuyuki Tokuyama, Reina Umeno, Yoshihisa Wada, Toshiya Yamamoto, Yu Yamanouchi, Masumi Abe, Yoko Inukai, Wataru Ogawa, Shunichiro Asahara, Hideki Fujii, Shunsuke Goto, Yushi Hirota, Tetsuya Hosooka, Keiji Kono, Shinichi Nishi, Yuko Okada, Kazuhiko Sakaguchi, Kenji Sugawara, Michiko Takahashi, Tomoko Takai, Yoshikazu Tamori, Kentaro Watanabe, Miyu Kitajima, Misaki Nishi, Junko Wada, Yasuhiko Ito, Hideki Kamiya, Akimasa Asai, Nao Asai, Saeko Asano, Shogo Banno, Yohei Ejima, Hanako Hase, Tomohide Hayami, Tatsuhito Himeno, Takahiro Ishikawa, Mayumi Ito, Shiho Iwagaitsu, Rina Kasagi, Yoshiro Kato, Makoto Kato, Koichi Kato, Takayuki Katsuno, Miyuka Kawai, Hiroshi Kinashi, Masaki Kondo, Masako Koshino, Naoya Matsuoka, Yoshiaki Morishita, Mikio Motegi, Jiro Nakamura, Hiromi Shimoda, Hirokazu Sugiyama, Shin Tsunekawa, Makoto Yamaguchi, Kazuyo Takahashi, Hirotaka Watada, Takashi Funayama, Yasuhiko Furukawa, Tomohito Gohda, Hiromasa Goto, Hideyoshi Kaga, Yasuhiko Kanaguchi, Akio Kanazawa, Kayo Kaneko, Toshiki Kano, Masao Kihara, Shogo Kimura, Takashi Kobayashi, Masayuki Maiguma, Yuko Makita, Satoshi Mano, Tomoya Mita, Takeshi Miyatsuka, Maki Murakoshi, Masahiro Muto, Masami Nakata, Junichiro Nakata, Yuya Nishida, Nao Nohara, Takeshi Ogihara, Daisuke Sato, Junko Sato, Hiroaki Sato, Yusuke Suzuki, Ruka Suzuki, Hitoshi Suzuki, Miyuki Takagi, Yoshifumi Tamura, Toyoyoshi Uchida, Seiji Ueda, Miki Asawa, Minako Miyaji, Eri Nagashima, Yoshie Shibata, Eri Yanagisawa, Toshimasa Yamauchi, Yosuke Hirakawa, Hiroshi Nishi, Nobuhiro Shojima, Satoko Horikawa, Yukiko Nakayama, Naoko Yamada, Yuki Omori, Shintaro Yano, Miyabi Ioka, Nahoko Kuwabara, Remi Nagano, Megumi Nozawa, Yumi Osawa, Hiroshi Maegawa, Shinji Kume, Shinichi Araki, Itsuko Miyazawa, Katsutaro Morino, Ikuko Kawai, Masumi Sobata, Motoko Takaoka, Yasushi Iwaita, Takashi Udagawa, Ami Inamori, Aya Kawase, Aya Yamanaka, Hitoshi Shimano, Akiko Fujita, Hitoshi Iwasaki, Hirayasu Kai, Yoshinori Osaki, Chie Saito, Motohiro Sekiya, Ryoya Tsunoda, Kunihiro Yamagata, Rikako Nakamura, Aiko Yamada, Mitsuru Ohsugi, Motoharu Awazawa, Ryotaro Bouchi, Shota Hashimoto, Makiko Hashimoto, Tomoko Hisatake, Noriko Ihana, Koko Ishizuka, Kazuo Izumi, Hiroshi Kajio, Michi Kobayashi, Noriko Kodani, Koji Maruyama, Michihiro Matsumoto, Maya Matsushita, Tomoka Nakamura, Takehiro Sugiyama, Akiyo Tanabe, Aiko Terakawa, Kojiro Ueki, Yuko Orimo, Takako Ozawa, Eriko Takahira, Yoshimitsu Yamasaki, Masakazu Haneda, Tadahiro Tomita, Saori Akimoto, Akihiro Fujimoto, Kenji Ishihara, Chiho Murakami, Akiyo Nishiyama, Yukiko Toyonaga, Kana Uozumi, Yukihiro Yamaji, Tetsuya Shigehara, Jun Okajyo, Yukihiro Shimizu, Shingo Iwasaki, Yuki Fukao, Megumi Furusho, Shintaro Nunokawa, Hideki Katagiri, Tomohito Izumi, Keizo Kaneko, Shinjiro Kodama, Mariko Miyazaki, Yuichiro Munakata, Tasuku Nagasawa, Yuji Oe, Hiroto Sugawara, Kei Takahashi, Kazushige Hirata, Keiko Inomata, Shoko Otomo, Taeko Uchida, Chigusa Yamashita, Arihiro Kiyosue, Ryota Tamura, Francois Dube, Marilene Bolduc, Marie-Christine Talbot, Leslie Cham, Vesta Lai, Josephine Tse, Shivinder Jolly, Tabbatha Duck, Scott Lyle, Rachel Epp, Camille Galloway, Susan Haskett, Elizabeta Matvienko, Liam Paulsen, Zachary Walbaum, Louise Moist, Zabrina Lozon, Tina Ramsey, Brittany Whitmore, Bader Al-Zeer, Paula Macleod, Aoife O'Sullivan, Zainab Sheriff, Sam Tholl, Amritanshu Pandey, Samantha Armstrong, Bethelihem Gebeyehu, Patrick Toth, Ronald Goldenberg, Mahsa Jahangiriesmaili, Shariff Sanguila, Neethi Suresh, Tanvi Talsania, Nadia Zalunardo, Mohsen Agharazii, Marie-Pier Roussel, Annie Saillant, France Samson, Harpreet Bajaj, Miken Bhavsar, Parul Dhall, Gagandeep Dhillon, Bhupinder Grewal, Taniya Nimbkar, Radica Richards, Julia Lee, Francois Madore, Guylaine Marcotte, Oren Steen, Mathura Bullen, Shayani Raguwaran, Andre Valleteau, Marie-France Langlois, Christine Brown, Andrew Steele, Melissa Garrity, Taneera Ghate, Holly Robinson, Michael Tolibas, Chetna Tailor, Lauren Elliott, Christine McClary-Wright, Fadia Boreky, Sameh Fikry, Ayesha Ali, Chintankumar Barot, Wagdy Basily, Thisun Saram, Vinay Varad, Karimula Mogal, Hasnain Khandwala, Alex Aguilera, Patricia Alvarez, Balwinder Gill, Nazihah Huda, Aamir Navivala, Daniel Pinto, Hitu Sharma, Micheli Bevilacqua, Elaine Fung, Geraldine Hernandez, Puneet Mann, Jaskiran Saini, Natasha Curtis, Remi Rabasa-Lhoret, Danijela Bovan, Marie Devaux, Cecilia Barnini, Giovanna Leoncini, Luca Manco, Giulia Nobili, Matteo Piemontese, Filippo Aucella, Rachele Grifa, Francesco Totaro, Gaetano La Manna, Irene Capelli, Giuseppe Cianciolo, Sarah Lerario, Fulvia Zappulo, Alberto Rosati, Filippo Fani, Giuseppe Spatoliatore, Loreto Gesualdo, Francesco Pesce, Maria Russo, Maria Zippo, Cesira Cafiero, Maria Ficarella, Marica Romano, Daria Motta, Simona Bianco, Donatella Bilucaglia, Piergiorgio Messa, Laura Pavone, Federica Tripodi, Simone Vettoretti, Giuseppe Castellano, Emilietta Brigati, Paola Fioretto, Gianni Carraro, Filippo Farnia, Anna Postal, Alessandro D'Amelio, Antonio Cardone, Giovanni Piccinni, Annalisa Aloisi, Francesco Scolari, Federico Alberici, Alice Guerini, Chiara Saccà, Chiara Salviani, Roberta Zani, Luca De Nicola, Carlo Garofalo, Maria Elena Liberti, Roberto Minutolo, Luigi Pennino, Lucio Polese, Paolo Mené, Simona Barberi, Clorinda Falcone, Francesco Russo, Maurizio Caroppo, Gennaro Santorelli, Rodolfo Rivera, Domenico Santoro, Alfio Giuffrida, Fortunata Zirino, Roberto Gallo, Cristina Calvi, Luca Estienne, Giovanni Gambaro, Concetta Gangemi, Vittorio Ortalda, Giuseppina Pessolano, Giuseppe Grandaliano, Rocco Baccaro, Pietro Ferraro, Roberto Mangiacapra, Marco Melandri, Nadia Foligno, Rita Quartagno, Giuseppe Vezzoli, Elena Brioni, Paola Maiucchi, Tunesi Francesca

**Affiliations:** a Health Economics Research Centre, Nuffield Department of Population Health, University of Oxford, Oxford, United Kingdom; b Clinical Trial Service Unit and Epidemiological Studies Unit, Nuffield Department of Population Health, University of Oxford, Oxford, United Kingdom; c Duke Clinical Research Institute, Durham, NC, USA; d University of Toronto, Toronto, Canada; e University of Washington, Seattle, WA, USA; f Providence Inland Northwest Health, Spokane, WA, USA; g Department of Clinical Studies and Epidemiology, Comprehensive Heart Failure Center, University Clinic, Würzburg, Germany; h Health Economics and Policy Research Unit, Wolfson Institute of Population Health, Queen Mary University of London, London, United Kingdom

**Keywords:** Chronic kidney disease (CKD), Empagliflozin, Quality-adjusted life-year (QALY), Health care use, Health care costs

## Abstract

**Background:**

Sodium-glucose co-transporter 2 inhibitors (SGLT2i) slow progression of chronic kidney disease (CKD) but there is no randomised evidence of their effects on health-related quality of life (QoL) and healthcare use. We explored the effects of empagliflozin on health-related QoL, healthcare use and UK healthcare costs in the EMPA-KIDNEY trial.

**Methods:**

EMPA-KIDNEY, a randomised, double blind, placebo-controlled, phase 3 trial, was conducted at 241 centres in eight countries (Canada, China, Germany, Italy, Japan, Malaysia, the UK, and the USA), and included participants aged 18 years or older with an estimated glomerular filtration rate (eGFR) of 20 to <45 mL/min/1.73 m^2^, or with an eGFR of 45 to <90 mL/min/1.73 m^2^ and a urinary albumin-to-creatinine ratio (uACR) of ≥200 mg/g at screening. They were randomly assigned (1:1) to receive empagliflozin 10 mg once daily or matching placebo. We estimated the effect of empagliflozin (UK£1.31/day) on exploratory outcomes (unless otherwise specified) of quality-adjusted life years (QALYs), UK costs (2023 UK£) of hospital admissions (a prespecified secondary outcome), concomitant medications and end-stage kidney disease (ESKD; a prespecified tertiary outcome) management over 2 years on study treatment (median active-trial follow-up) and on ESKD costs over 2 further years off study treatment (median post-trial follow-up) using shared parameter models analysing outcomes together with time to death or negative binomial models. The trial is registered with ClinicalTrials.gov, NCT03594110.

**Findings:**

Between May 15, 2019 and April 16, 2021, 6609 participants were randomly assigned to empagliflozin (3304 participants) or matching placebo (3305 participants) in the active-trial which lasted for a median of 2.0 years. Among them, 4891 (74%) were enrolled in the post-trial follow-up. Per participant allocated to empagliflozin over 2 years, total empagliflozin cost was £826 (95% confidence interval: 818 to 835), QALYs were 0.012 higher (0.001 to 0.022), with less cost for hospital admission (−£239, −449 to −29), concomitant medications (−£130, −214 to −47), and management of ESKD (−£208, −414 to −2) compared to placebo. Over a further 2 years of post-trial follow-up off study treatment, there were additional per participant ESKD cost savings (−£842, −1441 to −242), resulting in net total healthcare cost of −£593 (−1384 to 198) over 4 years. The probability of 2 years of empagliflozin treatment being cost-effective at £20 K threshold in the UK was 43% over 2 years of follow-up and 91% over 4 years. The relative effects of empagliflozin on each cost component were similar across categories by baseline levels of eGFR, uACR and diabetes status, with larger reductions in healthcare costs estimated in categories at higher risk of CKD progression.

**Interpretation:**

In EMPA-KIDNEY, 2 years treatment with empagliflozin improved QALYs, and reduced use and cost of other healthcare, resulting in high likelihood of cost-effectiveness across a broad range of patients with CKD. The study's key limitation is its relatively short active treatment period and follow-up duration, which may lead to underestimation of the cost-effectiveness of long-term SGLT2i treatment in CKD.

**Funding:**

10.13039/100008349Boehringer Ingelheim, Germany; 10.13039/100004312Eli Lilly, USA; 10.13039/501100000265Medical Research Council, UK; 10.13039/501100000274British Heart Foundation, UK; 10.13039/501100023699Health Data Research, UK; 10.13039/501100000272National Institute for Health and Care Research, UK.

**Research in context**

**Evidence before this study**

We searched PubMed for economic evaluations published between database inception and Sept 1 2024, using the search terms “CKD” or “chronic kidney disease” together with “sodium glucose transporter 2 inhibitors”, “sglt2”, “sglt-2”, “sodium-glucose cotransporter”, “empagliflozin”, “canagliflozin”, “dapagliflozin”, “ertugliflozin”, “ipragliflozin”, “luseogliflozin”, “remogliflozin”, “sergliflozin”, “sotagliflozin”, or “tofogliflozin”, and “economic evaluation”, “cost-effective”, “cost-effectiveness”, or “cost-utility”. All previous economic evaluations were model-based and reported that long-term treatment with sodium–glucose co-transporter-2 inhibitors (SGLT2i) is cost-effective in chronic kidney disease (CKD). These economic evaluations, however, lacked data for patients with CKD without diabetes or low levels of albuminuria (who constitute the majority of patients with CKD) and importantly relied on assumptions for CKD progression and long-term SGLT2i effects.

**Added value of this study**

The EMPA-KIDNEY trial recruited a broad range of patients with CKD, including patients with non-diabetic causes of kidney disease and with low albuminuria. Trial participants were observed over 2 years median follow-up in the active trial and for a further 2 years post-trial off study treatment enabling direct assessment of the effects of 2 years treatment with empagliflozin on end-stage kidney disease (ESKD) and its costs over about 4 years. We report that allocation to empagliflozin in EMPA-KIDNEY increased quality-of-life-adjusted survival and reduced the use and cost of other healthcare over 2 years. In the 2 years post-trial follow-up period off study treatment, there were further reductions in ESKD costs resulting in total net healthcare cost savings and high likelihood of cost-effectiveness over the 4 years of observation. The relative reductions in costs of hospital admissions, concomitant medications and ESKD management were similar across patient categories by diabetes status, estimated glomerular filtration rate (eGFR) and albuminuria, with larger absolute healthcare cost savings in those at higher risk of CKD progression.

**Implications of all the available evidence**

In addition to slowing progression of CKD and reducing risk of cardiovascular outcomes and hospitalisation, SGLT2i cost-effectively improve quality-of-life-adjusted survival and reduce ESKD and other healthcare costs in adults with CKD at risk of progression. These results should encourage more widespread prescription of SGLT2i to patients at risk of CKD progression. The study's key limitation is its relatively short active treatment period and follow-up duration, which may lead to underestimation of the cost-effectiveness of long-term SGLT2i treatment in CKD. Further prospective and modelling studies of the effects of long-term SGLT2i treatment will be helpful.

## Introduction

Large placebo-controlled trials and meta-analyses have shown that sodium-glucose cotransporter-2 inhibitors (SGLT2i) reduce the risk of kidney disease progression or cardiovascular death in a broad range of patients with chronic kidney disease (CKD) irrespective of diabetes status or levels of kidney function and albuminuria.^1^^,^^2^ In recent clinical practice guidelines, SGLT2i have been recommended for adults with CKD with an estimated glomerular filtration rate (eGFR) of 20 to less than 45 mL/min/1.73 m^2^, or with an eGFR of 45–90 mL/min/1.73 m^2^ with either type 2 diabetes or urine albumin-to-creatinine ratio (uACR) ≥ 200 mg/g.^3^, ^4^, ^5^ CKD is one of the leading causes of morbidity and mortality globally with substantial economic burden,^6^ which is especially pronounced in low- and middle-income countries where access to kidney dialysis or transplantation may be very limited.^7^

While there is good evidence that SGLT2i reduce risk of kidney disease progression and cardiovascular disease in a broad range of adults with CKD, their cost-effectiveness, particularly among those with non-diabetic kidney diseases or with low levels of albuminuria (or proteinuria), is uncertain. This may explain the low uptake of SGLT2i in CKD.^8^ Furthermore, although there are several model-based economic evaluations using trial data and published evidence, these all rely on assumptions about disease progression and long-term effects of SGLT2i.^9^, ^10^, ^11^, ^12^, ^13^

We aimed to use the EMPA-KIDNEY trial to address the lack of direct evidence for the effects of SGLT2i on quality of life (QoL) adjusted survival and healthcare use and costs. The trial is well-suited for this purpose as it included a broad range of patients with CKD, including those underrepresented in other trials.^1^ Other important design features of EMPA-KIDNEY which benefit economic assessments include the pre-specified key secondary outcome of all-cause hospitalisation and the post-trial follow-up of participants for a further 2 years after the end of the 2-year active-trial period. In the present analyses, we use all 4 years of follow-up data to assess the effects of 2 years of empagliflozin 10 mg daily vs placebo provided during the active trial.

## Methods

### Study design and participants

EMPA-KIDNEY was a randomised, controlled, phase 3 multinational trial, conducted in 241 centres across eight countries (ClinicalTrials.gov registration NCT03594110). Details of the EMPA-KIDNEY design and its key findings have been previously reported.^1^^,^^8^^,^^14^ The relevant regulatory authorities and research ethics committees/institutional review boards representing each centre approved the trial and its post-trial follow-up. Eligible patients were aged 18 years or older with a race-adjusted eGFR of 20–45 mL/min/1.73 m^2^ (irrespective of level of albuminuria) or eGFR of 45–90 mL/min/1.73 m^2^ with uACR ≥200 mg/g. Patients were prescribed a clinically appropriate dose of single-agent renin-angiotensin system (RAS) inhibitor where indicated and tolerated. Patients with or without diabetes were eligible. Patients with polycystic kidney disease and those on maintenance dialysis or with a functioning kidney transplant at randomisation were excluded. All the patients provided written informed consent.

Participants were randomly assigned (1:1) to receive oral empagliflozin (10 mg once daily) or matching placebo and seen in study clinics at 2 months, 6 months and every 6 months thereafter until the end of the active-trial period. At each follow-up visit, participants provided information on their kidney status (i.e. any dialysis treatment or receipt of a kidney transplant), adherence to study treatment (with reasons for stopping), and details of any concomitant medication. They were also asked in a structured interview about any serious adverse events, including hospital admissions and protocol-specified non-serious adverse events. Blood pressure and weight were measured and blood and urine samples were collected. The European Quality of Life 5-Dimensions 5-Level (EQ-5D-5L) questionnaire, assessing participants’ health-related QoL across five domains: mobility, self-care, usual activity, pain/discomfort, and anxiety/depression,^15^ was administered to study participants at baseline, and at the 18 months and final follow-up visits in the active trial. At the end of the active trial, study treatment was discontinued, and consenting participants were followed remotely for mortality, kidney status and key concomitant medication over a further 2 years of observation.^8^

### Outcomes

The health outcomes of interest in the present analysis were time to end-stage kidney disease (ESKD), a pre-specified tertiary trial outcome defined as initiation of maintenance dialysis or receipt of a kidney transplant, and hence a key determinant of healthcare costs attributed to CKD, and the exploratory outcomes of time to progression to a more advanced GFR-based CKD stage (e.g. from G3 to G4/5), health-related QoL and health-related QoL-adjusted survival. Health-related QoL was measured using the responses to the EQ-5D-5L questionnaire and the recommended UK valuation.^16^

The healthcare use outcomes and their costs included in the present analysis were all-cause first and subsequent hospital admissions (a prespecified secondary outcome in the trial) and the exploratory outcomes of hospital admission days, days on individual concomitant medications, days on ESKD management (dialysis and kidney transplantation) and days on study empagliflozin. Healthcare resource use reported during follow-up appointments were costed from the perspective of the UK National Health Service (NHS), including all-cause hospital admissions, concomitant medications, ESKD management (dialysis and kidney transplantation), and study drug empagliflozin during the active-trial period and ESKD management during the post-trial period. Hospital admissions were mapped into UK Healthcare Resource Groups (HRGs)^17^ and costed using national reference costs.^18^ Concomitant medications were costed based on the number of days the medication was used and its daily unit cost.^19^ Dialysis was costed based on the number of days on dialysis, the frequency of dialysis^20^ and the average cost per session.^18^ Kidney transplantation costs comprised of hospital admission for the transplant,^18^ and the maintenance costs of immunosuppressive drugs following transplantation (7 mg/day tacrolimus, 2 g/day mycophenolate mofetil and 5 mg/day prednisolone)^19^ until the end of the follow-up/transplant failure. Study drug empagliflozin in the empagliflozin arm was costed based on the follow-up periods in which participants were recorded to have taken ≥80% of allocated treatment using a daily empagliflozin cost of £1.31.^19^ All 2022 costs^18^ were inflated to 2023 values.^21^

### Statistical analysis

#### Effects of empagliflozin on CKD progression, QoL, healthcare use and costs

All analyses were performed according to the intention-to-treat principle, including all randomly assigned participants, and adjusting for allocation to empagliflozin and baseline variables specified in the minimisation algorithm (i.e. age, sex, region, eGFR, uACR, and previous diabetes).^1^

The effects of allocation to empagliflozin on time to ESKD and, separately, progression to more advanced CKD stage were assessed using pre-specified Cox proportional hazards models. The effect of empagliflozin on post-randomisation QoL was assessed using a post-hoc shared parameter model, with a linear mixed model of the QoL and Weibull survival model of time to death. The effects of allocation to empagliflozin on time to first and subsequent occurrences of hospital admissions were assessed using pre-specified joint frailty models, with both hazard functions for hospitalisations and for time to death conditional on the patient-specific random frailty term.^1^ Effects of allocation to empagliflozin on annualised days and costs of hospital admissions and, separately, concomitant medications were assessed using post-hoc shared parameter models, with mixed effect Poisson model of the rate of outcome and Weibull survival model of the time to death. Due to non-convergence of the shared parameter models, effects on annualised days and costs of ESKD management were estimated using negative binomial models, separately over active- and post-trial periods.

Tests for heterogeneity or trend in estimated effects were performed by including treatment allocation by subgroup interaction terms using pre-specified subgroup categories.

#### Net effects of empagliflozin on QALYs and healthcare use and costs

The net effects of allocation to empagliflozin over 2 years (median follow-up) were evaluated across all randomised trial participants in EMPA-KIDNEY using the models described above. Specifically, the effects on quality-adjusted life years (QALYs), days and costs of hospital admissions and concomitant medications were calculated using the shared parameter models. For ESKD management and study empagliflozin, the effects were calculated using the rate estimated from the negative binomial models (over active-trial and post-trial periods) and the survival estimated from a Cox proportional hazards model for the time to death over the entire follow-up. The estimated QALYs and costs were discounted using a discount rate of 3.5% per year.^22^ The total healthcare costs with and without empagliflozin were calculated by summing up the estimated costs of hospital admissions, concomitant medications, ESKD management and study empagliflozin over 2 years and, separately, adding the further 2 years post-trial ESKD costs. Incremental cost-effectiveness ratios (ICERs) were calculated over 2 years and over 4 years of follow-up using the respective incremental total healthcare costs divided by the incremental QALYs over 2 years. Uncertainty was estimated using 1000 bootstrap samples with replacement. Probabilities of being cost-effective at willingness to pay thresholds of £20,000 and £30,000 per QALY gained used by the National Institute for Health and Care Excellence were calculated. We summarised the estimated effects in participant categories by baseline values of eGFR, uACR, prior diabetes, and 5-year kidney failure risk. Sensitivity analyses with lower cost of empagliflozin at 50% and 90% of its current UK price were performed to assess the cost-effectiveness at lower prices.

Our approach was pre-specified in a Health Economic Analysis Plan finalised on October 4th, 2023 and, together with the trial protocol, is available at www.empakidney.org/downloads. The reporting of this study adheres to the Consolidated Health Economic Evaluation Reporting Standards (CHEERS) checklist. Further details and subsequent amendments are available in the Supplementary materials.

### Role of the funding source

EMPA-KIDNEY was designed, conducted, analysed and reported by the University of Oxford with a steering committee of international experts (the committee reviews trial publications). These analyses were performed on the original full database developed and held by the Nuffield Department of Population Health at the University of Oxford (Oxford, UK). The main trial funder (Boehringer Ingelheim) has minority representation on the trial Steering Committee (see Supplementary materials), which provided oversight of trial design, data collection, and data interpretation. The other funders of the study (including Eli Lilly) had no role in study design, data collection, data analysis, data interpretation, or writing of the report. JZ, NS, RH, WGH and BM are responsible for the analyses performed by the University of Oxford (Oxford, UK), where the original full database is held. BM, WH and RH had final responsibility for the decision to submit for publication.

## Results

Between May 15, 2019, and April 16, 2021, 6609 patients were randomised to either empagliflozin (n = 3304) or placebo (n = 3305) and followed for a median of 2.0 years (IQR 1.5–2.4) (Figure S1). At randomisation, the mean age of participants was 63.8 years, 33.2% were women, the mean eGFR was 37.3 mL/min/1.73 m^2^, the median uACR was 329 mg/g, 54% did not have diabetes, 36% had less than 5% risk of kidney failure over 5 years, and the mean QoL utility was 0.851 (Table 1). After the active trial, 4891 randomised participants (empagliflozin arm: n = 2472; placebo arm: n = 2419) were followed over a median of an additional 2.0 years (IQR 2.0–2.1) post-trial off-study-treatment period (Figure S1). During the post-trial period, both arms had balanced baseline characteristics (Table S1) and reported similar use of any SGLT2i (43% of participants who were allocated to empagliflozin in the active-trial period vs 40% in those allocated to placebo).

**Table 1:** Baseline characteristics of EMPA-KIDNEY participants, overall and by eGFR category and 5-year kidney failure risk.

	Overall (n = 6609)	eGFR (mL/min/1.73 m^2^)			5-year kidney failure risk		
		<30 (n = 2282)	30–45 (n = 2928)	≥45 (n = 1399)	<5% (n = 2377)	5%–20% (n = 2066)	≥20% (n = 2166)
**Demographic characteristics**							
Age (years)	63.8 (13.9)	65.8 (13.2)	64.9 (13.4)	58.3 (14.6)	66.4 (13.2)	64.7 (13.6)	60.2 (14.1)
Sex: Female	2192 (33.2%)	749 (32.8%)	991 (33.8%)	452 (32.3%)	945 (39.8%)	653 (31.6%)	594 (27.4%)
Race							
White	3859 (58.4%)	1440 (63.1%)	1833 (62.6%)	586 (41.9%)	1508 (63.4%)	1294 (62.6%)	1057 (48.8%)
Black	262 (4.0%)	98 (4.3%)	119 (4.1%)	45 (3.2%)	91 (3.8%)	85 (4.1%)	86 (4.0%)
Asian	2393 (36.2%)	707 (31.0%)	930 (31.8%)	756 (54.0%)	756 (31.8%)	658 (31.8%)	979 (45.2%)
Mixed	21 (0.3%)	6 (0.3%)	13 (0.4%)	2 (0.1%)	3 (0.1%)	9 (0.4%)	9 (0.4%)
Other	74 (1.1%)	31 (1.4%)	33 (1.1%)	10 (0.7%)	19 (0.8%)	20 (1.0%)	35 (1.6%)
Region							
Europe (UK, Germany, Italy)	2648 (40.1%)	1026 (45.0%)	1208 (41.3%)	414 (29.6%)	1096 (46.1%)	866 (41.9%)	686 (31.7%)
North American (USA, Canada)	1717 (26.0%)	602 (26.4%)	868 (29.6%)	247 (17.7%)	567 (23.9%)	587 (28.4%)	563 (26.0%)
China, Malaysia	1632 (24.7%)	520 (22.8%)	635 (21.7%)	477 (34.1%)	414 (17.4%)	468 (22.7%)	750 (34.6%)
Japan	612 (9.3%)	134 (5.9%)	217 (7.4%)	261 (18.7%)	300 (12.6%)	145 (7.0%)	167 (7.7%)
**Prior disease**							
Prior diabetes^a^	3040 (46.0%)	1151 (50.4%)	1371 (46.8%)	518 (37.0%)	1027 (43.2%)	966 (46.8%)	1047 (48.3%)
Prior diabetes type							
Type 1	68 (2.2%)	31 (2.7%)	28 (2.0%)	9 (1.7%)	16 (1.6%)	23 (2.4%)	29 (2.8%)
Type 2	2936 (96.6%)	1106 (96.1%)	1333 (97.2%)	497 (95.9%)	996 (97.0%)	938 (97.1%)	1002 (95.7%)
Other/unknown	36 (1.2%)	14 (1.2%)	10 (0.7%)	12 (2.3%)	15 (1.5%)	5 (0.5%)	16 (1.5%)
History of cardiovascular disease^b^	1765 (26.7%)	718 (31.5%)	828 (28.3%)	219 (15.7%)	639 (26.9%)	579 (28.0%)	547 (25.3%)
**Clinical measurements**							
Systolic blood pressure (mmHg)	136.5 (18.3)	137.6 (18.6)	136.0 (18.2)	135.9 (17.8)	134.2 (18.1)	135.7 (18.0)	139.9 (18.2)
Diastolic blood pressure (mmHg)	78.1 (11.8)	76.5 (11.8)	77.9 (11.7)	80.9 (11.6)	77.5 (11.7)	76.9 (11.8)	79.8 (11.8)
Body mass index (kg/m^2^)	29.7 (6.8)	30.1 (6.7)	30.1 (6.9)	28.5 (6.5)	29.6 (6.6)	30.1 (7.1)	29.5 (6.6)
**Laboratory measurements**							
eGFR (mL/min/1.73 m^2^)^c^	37.3 (14.4)	24.6 (3.6)	36.8 (4.2)	59.3 (13.5)	48.6 (15.5)	35.3 (9.5)	26.9 (6.0)
<30	2282 (34.5%)	2282 (100.0%)	NA	NA	121 (5.1%)	614 (29.7%)	1547 (71.4%)
30–45	2928 (44.3%)	NA	2928 (100.0%)	NA	1104 (46.4%)	1215 (58.8%)	609 (28.1%)
≥45	1399 (21.2%)	NA	NA	1399 (100.0%)	1152 (48.5%)	237 (11.5%)	10 (0.5%)
uACR (mg/g)^c^, ^d^	329 (49–1069)	410 (59–1373)	187 (26–781)	515 (214–1199)	62 (10–413)	212 (54–674)	1060 (448–2055)
<30	1328 (20.1%)	386 (16.9%)	789 (26.9%)	153 (10.9%)	983 (41.4%)	328 (15.9%)	17 (0.8%)
30–300	1864 (28.2%)	639 (28.0%)	896 (30.6%)	329 (23.5%)	664 (27.9%)	852 (41.2%)	348 (16.1%)
>300	3417 (51.7%)	1257 (55.1%)	1243 (42.5%)	917 (65.5%)	730 (30.7%)	886 (42.9%)	1801 (83.1%)
NT-proBNP (ng/L)	160 (69–419)	270 (114–637)	151 (71–388)	81 (15–175)	128 (55–324)	172 (70–437)	191 (88–489)
**Concomitant medication use**							
RAS inhibitor	5628 (85.2%)	1872 (82.0%)	2487 (84.9%)	1269 (90.7%)	2035 (85.6%)	1758 (85.1%)	1835 (84.7%)
Any diuretic	2815 (42.6%)	1151 (50.4%)	1271 (43.4%)	393 (28.1%)	973 (40.9%)	938 (45.4%)	904 (41.7%)
Any lipid-lowering medication	4378 (66.2%)	1657 (72.6%)	1955 (66.8%)	766 (54.8%)	1523 (64.1%)	1355 (65.6%)	1500 (69.3%)
**Cause of kidney disease**							
Diabetic kidney disease	2057 (31.1%)	801 (35.1%)	901 (30.8%)	355 (25.4%)	631 (26.5%)	640 (31.0%)	786 (36.3%)
Hypertensive/renovascular disease	1445 (21.9%)	533 (23.4%)	699 (23.9%)	213 (15.2%)	576 (24.2%)	478 (23.1%)	391 (18.1%)
Glomerular disease	1669 (25.3%)	452 (19.8%)	636 (21.7%)	581 (41.5%)	572 (24.1%)	487 (23.6%)	610 (28.2%)
Other/unknown	1438 (21.7%)	496 (21.7%)	692 (23.6%)	250 (17.9%)	598 (25.2%)	461 (22.3%)	379 (17.5%)
**Health related quality of life**							
Quality of life^e^	0.851 (0.173)	0.833 (0.180)	0.845 (0.178)	0.891 (0.143)	0.842 (0.180)	0.844 (0.171)	0.866 (0.166)

Figures are n (%), mean (SD) or median (Q1-Q3). NT-proBNP, N-terminal pro B-type natriuretic peptide. eGFR, estimated glomerular filtration rate. uACR, urine albumin-to-creatinine ratio. RAS, renin-angiotensin system.

a Participant-reported history of diabetes of any type, use of glucose-lowering medication or baseline HbA1c ≥ 48 mmol/mol at randomisation.

b Self-reported history of myocardial infarction, heart failure, stroke, transient ischemic attack, or peripheral arterial disease.

c Uses central measurement taken at the randomisation visit, or more recent local laboratory result before randomisation.

d To convert uACR from mg/g to mg/mmol units please divide by 8.84.

e EQ-5D utility.

During the active-trial follow-up, 266 participants progressed to ESKD. Allocation to empagliflozin yielded a 33% reduction in the risk of progression to ESKD (hazard ratio [HR] 0.67, 95% confidence interval [CI] 0.52–0.85) as well as reductions in risk of progression to more advanced CKD stages. Over the post-trial period, additional 402 participants progressed to ESKD. Over the active-trial and post-trial off-treatment follow-up periods, allocation to empagliflozin yielded a 26% reduction in the risk of progression to ESKD (HR 0.74, 0.64–0.87) (Table S2).

During the active-trial follow-up, allocation to empagliflozin yielded a 14% reduction in hospital admissions (HR 0.86, 95% CI 0.78–0.95) with consistent, though more uncertain, effects on days in hospital (rate ratio [RR] 0.82; 95% CI 0.64–1.03) and costs of hospital admissions (RR 0.81, 0.56–1.19) (Table S3). During the active-trial follow-up, despite the small effect on days on concomitant medications (RR 0.98, 0.95–1.00), allocation to empagliflozin resulted in a 10% (RR 0.90, 0.85–0.96) proportional reduction in costs of concomitant medications (due to the reduced use of some antihypertensive therapies; Tables S3–S4). The significant reduction in risk of ESKD was accompanied by consistent, albeit more uncertain, reductions in days in ESKD (RR 0.77, 0.41–1.47) and costs of ESKD management (RR 0.74, 0.33–1.70) over active-trial period, with similar effects over the post-trial period (Tables S3 and S5).

During the active-trial follow-up, 6163 participants completed an EQ-5D assessment by the 18-month visit (mean [SD] 1.45 [0.12] years after randomisation), and 3670 participants completed EQ-5D assessment at a subsequent visit (mean [SD] 2.37 [0.32] years after randomisation). Mean differences in QoL between empagliflozin- and placebo-allocated participants at these time points were 0.007 (95% CI, −0.002 to 0.015) and 0.005 (−0.003 to 0.013) respectively (Table S3).

**Fig. 1:**
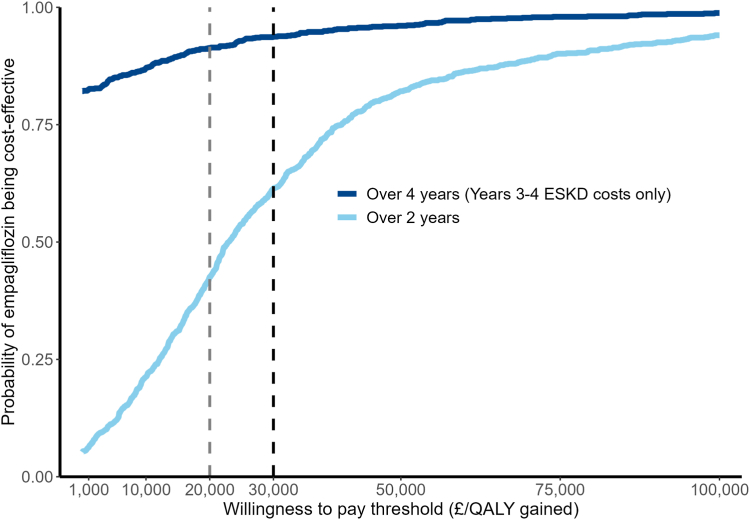
**Probability of 2 years treatment with empagliflozin being cost-effective over 2 and 4 years.** The light blue and dark blue solid lines represent the probability of 2 years treatment with empagliflozin being cost-effective over 2 years or 4 years, respectively, across different willingness to pay thresholds. Probabilities of empagliflozin being cost-effective over 2 years were 43% and 61% and over 4 years were 91% and 94% at willingness to pay thresholds of £20,000 and £30,000 per QALY gained (grey and black dashed lines), respectively. ESKD = end-stage kidney disease (i.e. maintenance dialysis or kidney transplantation); QALY = quality-adjusted life year.

Over the 2 years active-trial period, per participant allocated to empagliflozin (mean days on empagliflozin 637 (630 to 643)), there were 0.012 (0.001 to 0.022) mean QALYs gained and mean reductions in days in hospital of 0.9 days (0.0 to 1.8), in days on individual concomitant medications of 72 days (−33 to 177), and in days in ESKD of 2.3 days (−0.3 to 4.9) (Table 2). Allocation to empagliflozin for 2 years at empagliflozin cost of £826 (818 to 835) led to cost savings from hospital admissions −£239 (−449 to −29), from concomitant medications −£130 (−214 to −47), and from ESKD management −£208 (−414 to −2) resulting in net total healthcare costs of £249 (−75 to 573) per participant and an incremental cost per QALY gained of £21,515 with 43% to 61% probability of being cost-effective at £20,000 to £30,000/QALY thresholds (Table 2, Fig. 1, Figure S2).

**Table 2:** Estimated effects of 2 years treatment with empagliflozin on participants’ quality-adjusted life years and healthcare use and costs in EMPA-KIDNEY.

	Mean (95% CI)		Difference in means (95% CI)
	Empagliflozin	Placebo	
**Over 2 years (based on ACTIVE-TRIAL data)**			
QALYs	*1.641 (1.631, 1.651)*	*1.629 (1.619, 1.640)*	*0.012 (0.001, 0.022)*
Days of healthcare use			
Study empagliflozin^a^	637 (630, 643)	–	637 (630, 643)
Hospital admissions	4.4 (3.8, 4.9)	5.2 (4.6, 5.9)	−0.9 (−1.8, 0.0)
Individual concomitant medications^b^	3478 (3411, 3545)	3550 (3481, 3620)	−72 (−177, 33)
ESKD management^c^	7.9 (6.1, 9.7)	10.2 (8.3, 12.0)	−2.3 (−4.9, 0.3)
Costs of healthcare use			
Study empagliflozin^a^	£826 (818, 835)	–	£826 (818, 835)
Hospital admissions	£1080 (952, 1207)	£1319 (1161, 1477)	£−239 (−449, −29)
Concomitant medications^b^	£1228 (1166, 1289)	£1358 (1295, 1421)	£−130 (−214, −47)
ESKD management^c^	£606 (465, 747)	£814 (664, 965)	£−208 (−414, −2)
Total healthcare costs^d^	*£3740 (3529, 3951)*	*£3491 (3253, 3730)*	*£249 (*−*75, 573)*
**Over a further 2 years (based on POST-TRIAL data)**			
Days of ESKD management^c^	46 (40, 52)	57 (50, 63)	−11 (−20, −2)
Costs of ESKD management^c^	£3153 (2747, 3559)	£3995 (3565, 4425)	£−842 (−1441, −242)
**Over 4 years (Years 3**–**4 include only ESKD management costs)**			
Total healthcare costs^d^	*£6894 (6374, 7413)*	*£7487 (6919, 8054)*	*£*−*593 (*−*1384, 198)*

Unless otherwise specified, results were derived using the estimated shared parameter models. Total outcomes (in italic) are used to calculate the incremental cost-effectiveness ratios (ICERs).

ESKD, end-stage kidney disease (i.e. maintenance dialysis or kidney transplantation); QALY, quality-adjusted life year.

a Days and costs of study empagliflozin were estimated using a negative binomial model among empagliflozin-allocated participants adjusted for baseline variables specified in the minimization algorithm (age, sex, previous diabetes, estimated glomerular filtration rate, urine albumin-to-creatinine ratio, and region) and Cox proportional hazards model for time to death adjusted for allocation to empagliflozin and baseline variables in the minimization algorithm.

b Concomitant medications included were antihypertensive treatments, antiplatelets, anticoagulants, diabetes medications, lipid lowering medications, drug for anaemia, uric acid lowering medications, and phosphate binders. Each day on individual concomitant medication contributes a ‘medication day’ in the total concomitant medication days.

c ESKD management costs (i.e. costs of providing maintenance dialysis or kidney transplantation) were estimated using a negative binomial model adjusting for allocation to empagliflozin only for ESKD costs together with the Cox proportional hazards model for time to death adjusting for allocation to empagliflozin and baseline variables specified in the minimization algorithm.

d Total costs were derived by adding the separate cost components.

Over the 2 year post-trial period, per participant originally allocation to empagliflozin there were fewer days in ESKD (−11, −20 to −2) and lower ESKD costs (−£842, −1441 to −242) (Table 2). Overall, adding these cost savings from reduced progression to ESKD to the effects within 2 years active-trial period, allocation to empagliflozin treatment for 2 years led to per participant net total healthcare costs of −£593 (−1384 to 198) over 4 years, resulting in 91% to 94% probability of being cost-effective at thresholds of £20,000 to £30,000 per QALY gained (Table 2, Fig. 1, Figure S2).

**Fig. 2:**
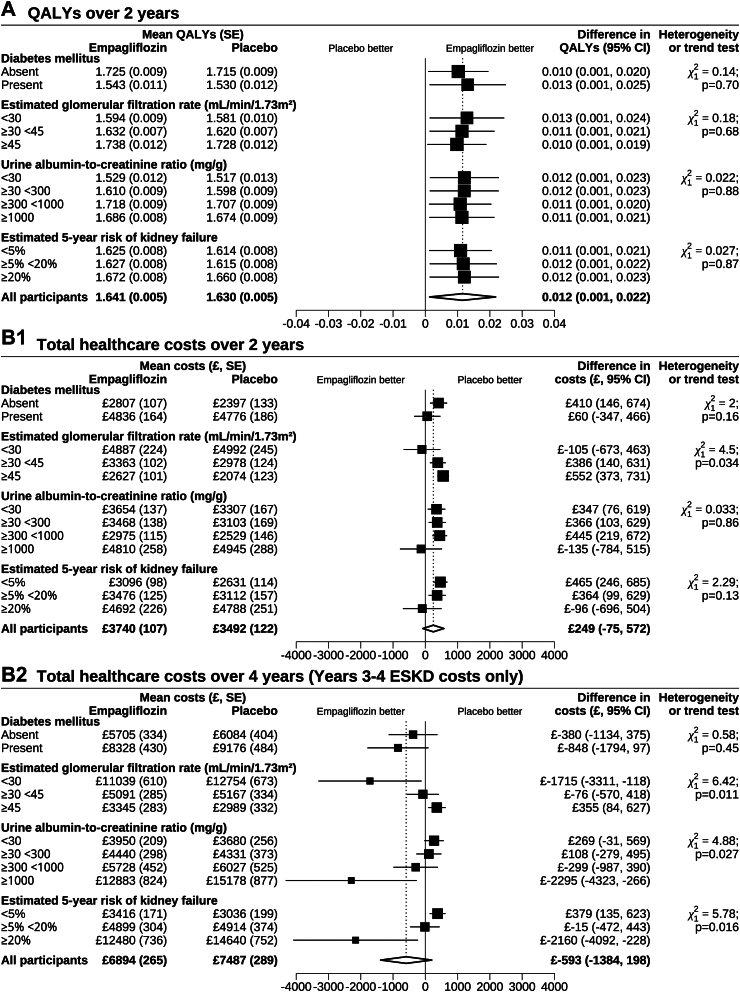
**Effects of 2 years treatment with empagliflozin on QALYs and total healthcare costs, by categories of patients with CKD.** The diamonds indicate the estimated effects of 2 years treatment with empagliflozin on the corresponding outcomes across all participants and the squares indicate respective effects in categories of participants. The error bars indicate the 95% confidence intervals of the estimated effects. ESKD = end-stage kidney disease (i.e. maintenance dialysis or kidney transplantation); QALY = quality-adjusted life year.

Across categories of participants, the proportional effects of allocation to empagliflozin on QoL, costs of hospital admissions, concomitant medications, and ESKD management were similar across categories of participants (Tables S6–S10). Using the overall proportional effects of allocation to empagliflozin, similar net absolute effects of allocation to empagliflozin were estimated over the 2 years active-trial period in key categories of participant, including those with and without diabetes, and across the spectrum of albuminuria, eGFR and kidney failure risk (Table S11). Adding the 2 years post-trial ESKD costs, larger net total healthcare cost savings were estimated among participants at higher risk of CKD progression (Fig. 2, Figure S3). Overall, 2 years of treatment with empagliflozin was highly cost-effective in most categories of participants over 4 years of follow-up in EMPA-KIDNEY (Figure S4).

**Fig. 3:**
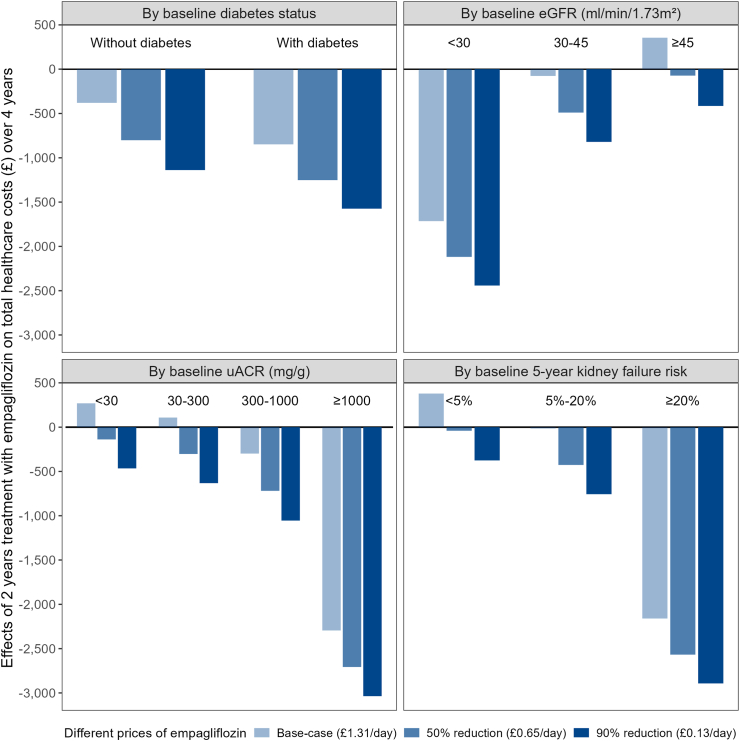
**Effects of 2 years treatment with empagliflozin on total healthcare costs over 4 years (Years 3**–**4 ESKD costs only) in categories of patients with CKD, at different empagliflozin cost**. The bars indicate the effects of 2 years treatment with empagliflozin on total healthcare costs over 4 years in categories of patients with CKD at different empagliflozin cost (light blue: with current empagliflozin cost (base-case); medium shade of blue: with 50% reduction in empagliflozin cost; dark blue: with 90% reduction in empagliflozin cost). ESKD = end-stage kidney disease (i.e. maintenance dialysis or kidney transplantation); eGFR = estimated glomerular filtration rate. uACR = urine albumin-to-creatinine ratio.

The relative effects of allocation to empagliflozin on hospital admissions, days in hospital and costs of hospital admissions were similar across types of admissions (Tables S12–S14). The relative effects on days in hospital were also similar in a sensitivity analysis after adjusting the length of stay per hospital admission from the regions outside Europe to the length of stay in Europe (results not shown), and the relative effects on days of and costs of ESKD management by modality (dialysis or transplantation) were also similar (Table S15). At 50% lower price of empagliflozin, allocation to 2 years of empagliflozin treatment led to statistically significant lower total healthcare costs over 4 years, overall and in most categories of participants (and in all categories at 90% lower price), and was highly cost-effective in all categories of participants (Fig. 3, Figures S5–S6).

## Discussion

In EMPA-KIDNEY, 2 years of treatment with empagliflozin led to a small but measurable improvement in QALYs and lower costs for hospital admissions, concomitant medications and ESKD management. These cost savings offset about three quarters of the cost of empagliflozin during the median 2 years follow-up in the active trial. The further reductions in ESKD cost, observed during the 2 years post-trial follow-up period, led to net total healthcare cost savings and high probability of cost-effectiveness over 4 years. The relative effects of empagliflozin on costs of hospital admissions, medications and ESKD management were similar across categories of participants including by diabetes status, level of kidney function and albuminuria, and kidney failure risk, with larger total healthcare cost savings over 4 years estimated in categories at higher risk of CKD progression.

A unique feature of these analyses is the direct assessment of randomised effects of about 2 years of empagliflozin on quality-of-life-adjusted survival and healthcare costs over 2 years and costs of ESKD management over 4 years. The availability of post-trial follow-up data in EMPA-KIDNEY allowed us to more fully assess the effect of empagliflozin on the risk of progression to, and costs of, ESKD across different categories of patients with CKD. Our findings indicate that empagliflozin reduced ESKD costs in all categories of patients with CKD regardless of diabetes status, level of albuminuria or kidney function, or primary kidney diagnosis, with larger effects among those at greater risk of progression to ESKD. The consistency of effects across categories of patients indicate that empagliflozin is likely to confer benefits also among patients with CKD without albuminuria although it may take somewhat longer for benefits to emerge.

The effects of empagliflozin on CKD progression and cardiovascular outcomes in EMPA-KIDNEY were quantitatively similar to effects reported in other SGLT2i trials^1^^,^^23^ and its effects on QALYs and healthcare costs, reported here, are likely generalizable to other SGLT2 inhibitors. While longer-term SGLT2i use is expected to lead to ongoing benefits on the risk of CKD progression and cardiovascular morbidity and mortality and, therefore, larger effects on QALYs and healthcare costs than reported here, the direct assessment reported here avoids the assumptions for CKD progression and long-term effects of SGLT2i made in previous exclusively model-based cost-effectiveness studies of SGLT2i,^9^^,^^10^^,^^12^ and provides useful conservative estimates of effects.

Our results indicate that SGLT2i treatment is likely to be highly cost-effective in managing CKD in the UK, supporting current UK National Institute for Health and Care Excellence (NICE) guidance on use of SGLT2i in CKD.^4^^,^^5^ The 0.012 difference in QALYs over 2 years captures the gain in quality-of-life-adjusted survival and corresponds to 4.4 extra days in full health per participant. This benefit is despite only 2 years of study treatment in a population with CKD who were at relatively low risk of death or ESKD during the short active-trial period.^1^ Although quality of life data were not collected during the post-trial follow-up periods, the sustained treatment effect on delaying progression to ESKD over this period, indicate that further QALY gains would be expected with longer term treatment. The generalizability of our findings also depends on the level of adherence to SGLT2i. Among patients randomised to empagliflozin in EMPA-KIDNEY, 92% were using it at 12 months and 88% at 24 months.^8^ A recent multinational study reported that among patients with CKD initiated on SGLT2i in 2012–2021, 50%–72% remained on treatment one year later.^24^ Lower adherence is expected to lead to smaller benefits.

SGLT2i are widely used in many countries to treat diabetes and heart failure and more recently also widely approved and recommended for treating adults with CKD.^5^^,^^25^^,^^26^ The question as to whether SGLT2i provide good value for money in CKD is important in view of the considerable impact on healthcare budgets given the global burden of CKD.^6^ In the present analyses, the effects of empagliflozin on QoL and healthcare use (i.e. hospital admissions, days in hospital and days on concomitant medications) in CKD, were similar in categories of participants by study region. Although we analysed QoL and healthcare costs using UK EQ-5D tariff and costs, our findings are likely to be relevant to clinical management of CKD in other regions. There are strong recommendations from international guidelines for the use of SGLT2i in patients with CKD with diabetes and those without diabetes in the context of albuminuria above a certain threshold.^3^ Our analyses suggest that expanding access to patients with CKD without diabetes and with little albuminuria is likely to benefit patients and healthcare systems. Moreover, following the expiry of patent protection of SGLT2i, lower-cost generic SGLT2i with high probability of price reductions reaching 50%^27^ and even 90%^28^ are expected to become available. At these lower price levels, our sensitivity analyses indicate that SGLT2i treatment would be cost-saving or highly cost-effective within several years across the broad range of patients with CKD recruited into EMPA-KIDNEY, irrespective of diabetes status or levels of kidney function and albuminuria. This is particularly relevant in contemporary practice as generic SGLT2 inhibitors at lower prices are already available in some regions. Our findings, driven by ESKD cost savings, are likely to be replicated in other countries, including low- and middle-income countries where ESKD management can be unaffordable.^29^ Moreover, the delayed progression to ESKD with SGLT2i may result in larger gains in survival and QALYs in settings with limited access to kidney dialysis or transplantation.^7^

A key strength of these analyses is the direct trial-based economic evaluation of SGLT2i in the broad CKD population in the large EMPA-KIDNEY trial. The use of shared parameter models for estimating the effects of empagliflozin on hospital admissions, concomitant medications and quality of life, integrating the dependence between the risk of death and healthcare use and quality of life, helps ensure reliable estimates of the net effects of treatment. The study's key limitation is its relatively short active treatment period and follow-up duration. The study's findings for about 2 years of treatment underestimate the cost-effectiveness of long-term SGLT2i treatment in CKD. The additional 2 years post-trial follow-up data helped partially mitigate this limitation but analyses with longer treatment duration and over longer time periods are needed to evaluate more fully the value of SGLT2i′s, particularly in patients with lower ESKD risk. Further prospective and modelling studies of the effects of long-term SGLT2i treatment, and adherence with treatment, in categories of patients with CKD, including by risk of ESKD, will be helpful. Moreover, the streamlined post-trial follow-up design did not allow assessment of potential benefits on quality of life or savings from hospital admissions and concomitant medication use post-trial. The healthcare resources considered in our analyses also did not include primary care and outpatient hospital services beyond dialysis and transplantation; these costs are, however, relatively small compared to the costs of hospital care^30^ and ESKD included in the analyses.

In summary, 2 years of allocation to empagliflozin in EMPA-KIDNEY increased quality-of-life-adjusted survival and reduced use and cost of other healthcare across a broad range of patients with CKD. Further ESKD cost savings were observed during the 2 additional years of follow-up off study treatment, confirming that empagliflozin is a highly cost-effective treatment in adults with CKD.

## Contributors

BM, RH and WGH developed the concept for these analyses. JZ, NS, RH, WGH & BM had full access to all trial data, verified the underlying data, and take responsibility for its integrity and analysis. WGH, RH, MJL, NS, DP, JRE, CB designed the trial and acquired funding (provided by Boehringer Ingelheim). The EMPA-KIDNEY Collaborative Group collected the data. JZ wrote the first draft of the manuscript, under supervision from BM and WGH. All authors contributed to interpretation of the results and manuscript revision. BM, WH and RH had final responsibility for the decision to submit for publication.

## Data sharing statement

The complete de-identified patient data set used for presented analyses will be available in due course. Departmental policy details can be found here: https://www.ndph.ox.ac.uk/data-access.

## Declaration of interests

The EMPA-KIDNEY trial was initiated, designed, conducted, analysed and reported by the University of Oxford with a Steering Committee of experts. This paper has not been published previously in whole or part. The Clinical Trial Service Unit and Epidemiological Studies Unit (Oxford, UK) has a staff policy of not accepting honorarium or other payments from the pharmaceutical industry, except for the reimbursement of costs to participate in scientific meetings (see https://www.ctsu.ox.ac.uk/about/ctsu_honoraria_25june14-1.pdf).

JZ, CW, NS, PKJ, KJM, NA, RA, DP, ChW, MJL, CB, RH, WGH, BM report grant funding paid to their institution (the University of Oxford) from Boehringer Ingelheim and Eli Lilly, and funding from the United Kingdom Medical Research Council (MRC) (to the Clinical Trial Service Unit and Epidemiological Studies Unit; reference no., MC_UU_00017/3), the British Heart Foundation, National Institute for Health and Care Research Biomedical Research Council, and Health Data Research (UK). Additionally: NS reports institutional grant funding from Novo Nordisk; and leadership role in Nephrology Dialysis and Transplant journal. PKJ reports institutional grant funding from Novartis. DP additionally reports institutional grant funding from Novo Nordisk. ChW additionally reports consultancy fees or honoraria for lectures as part of his affiliation with Wurzburg from VeraTX, Alexion, MSDBayer, Amgen, AstraZeneca, Bayer, Boehringer Ingelheim, CSLVifor, Lilly, NovoNordisk; board membership of AKTN – Australasian Kidney Trials network; and a leadership role in European Renal Association. MJL additionally reports institutional grant funding from Novartis, Janssen, GV, Flu Lab, Schmidt Future, NHS England, Wellcome, Bill & Melinda Gates Foundation; research contract with Sanofi, Regeneron, Moderna, BioNTech, Apollo Therapeutics, Verve Therapeutics and GSK; support to attend meetings from Boehringer Ingelheim; unpaid advisory role to European Society of Cardiology. CB additionally reports institutional grant funding from NIHR HTA and Health Data Research UK; participation on a Data Safety Monitoring Board or Advisory Board related to Merck, NIHR HTA, the British Heart Foundation; and leadership roles as European Society of Cardiology Chair of Committee on Practice Guidelines and with NIHR HTA (Chair: ATTACK: Aspirin To Target Arterial Events in Chronic Kidney Disease; & DASH: Desmopressin for acute stroke due to haemorrhage). RH additionally reports participation on a Data Safety Monitoring Board or Advisory Board related to Lilly (no payments received). WGH additionally reports personal fellowship grant from Kidney Research UK. BM additionally reports institutional grant funding from NIHR (HTA, Barts Biomedical Research Centre); support to attend meetings from the European Society of Cardiology and Kidney Disease Improving Global Outcomes; and unpaid role on the ESC Clinical Practice Guidelines Committee. JBG reports institutional grant funding from Merck, Roche, Boehringer Ingelheim, Lilly, Bluedrop; and consulting fees from AstraZeneca, Corcept, NovoNordisk, Bayer, Boehringer Ingelheim, Valo, Lilly, Vertex, Mineralys. DZIC has received honoraria from Boehringer Ingelheim-Lilly, Merck, AstraZeneca, Sanofi, Mitsubishi-Tanabe, Abbvie, Janssen, AMGEN, Bayer, Prometic, BMS, Maze, Gilead, CSL-Behring, Otsuka, Novartis, Youngene, Lexicon, Inversago, GSK, Biobridge, Vantage, Altimmune and Novo-Nordisk and has received operational funding for clinical trials from Boehringer Ingelheim-Lilly, Merck, Janssen, Sanofi, AstraZeneca, CSL-Behring and Novo-Nordisk, Bayer. KRT reports grant funding from the National Institutes of Health, NIH (NIDDK, NHLBI, NCATS, NIMHD), the Centers for Disease Control and Prevention (CDC), Travere, Bayer and Doris Duke Foundation; consulting fees from Lilly, Boehringer Ingelheim, Astra Zeneca, Novo Nordisk, Bayer and ProKidney; honoraria from Astra Zeneca, Novo Nordisk and Bayer; support to attend meetings from Novo Nordisk; support for role on Data Safety Monitoring Board from AstraZeneca; unpaid roles on Data Safety Monitoring/Advisory Boards for NIDDK and George Clinical; paid roles on Data Safety Monitoring Board for AstraZeneca; and unpaid leadership roles as Chair, Diabetic Kidney Disease Collaborative Taskforce, American Society of Nephrology and Board of Directors, Kidney Disease Improving Global Outcomes, and Controversies Conference on Kidney Disease Prevention, and Chair, Program Committee for Kidney Week 2025, American Society of Nephrology. JL and PC declare no competing interests.
