# Supplementary material for: Effects of empagliflozin on quality of life and healthcare use and costs in chronic kidney disease: a health economic analysis of the EMPA-KIDNEY trial
Source: eClinicalMedicine. 2025 Jul 8;85:103338. doi: 10.1016/j.eclinm.2025.103338 (PMC12274867; doi:10.1016/j.eclinm.2025.103338)
Supplement: Supplementary materials [file mmc1.docx]

**Effects of empagliflozin on quality of life and healthcare use and costs in chronic kidney disease: a health economic analysis of the EMPA-KIDNEY trial**

**Supplementary material**

**Table of Contents**

[Members of the EMPA-KIDNEY Collaborative Group 3](#_Toc200462836)

[Supplementary methods 12](#_Toc200462837)

[Supplementary tables and figures 18](#_Toc200462838)

[Supplementary Figure S1. CONSORT participant flowchart for active-trial and post−trial follow-up (PTFU) periods 18](#_Toc200462839)

[Supplementary table S1. Characteristics at randomization of participants entering and not entering post-trial follow-up 19](#_Toc200462840)

[Supplementary table S2. Effect of allocation to empagliflozin on **time to first ESKD**, **time to CKD5 or ESKD and time to CKD4, CKD5 or ESKD** 20](#_Toc200462841)

[Supplementary table S3. Effects of allocation to empagliflozin on **QoL and on hospital admissions, days of and costs of healthcare use** 20](#_Toc200462842)

[Supplementary table S4. Effects of allocation to empagliflozin on **days on and costs of individual concomitant medication of interest**, overall and by each category of interest (Limited to active-trial data) 21](#_Toc200462843)

[Supplementary table S5. Effect of 2 years treatment with empagliflozin on **days of and costs of ESKD management**, by year of analysis 22](#_Toc200462844)

[Supplementary figure S2. Incremental costs and incremental QALYs over 2 and 4 years with allocation to 2 years empagliflozin treatment in EMPA-KIDNEY (1000 bootstrapping samples) 23](#_Toc200462845)

[Supplementary table S6. Effect of allocation to empagliflozin on **QoL**, overall and by subgroups at baseline (Limited to active-trial data) 24](#_Toc200462846)

[Supplementary table S7. Effect of allocation to empagliflozin on **hospital costs**, overall and by subgroups at baseline (Limited to active-trial data) 25](#_Toc200462847)

[Supplementary table S8. Effect of allocation to empagliflozin on **costs of concomitant medications of interest**, overall and by subgroups at baseline (Limited to active-trial data) 26](#_Toc200462848)

[Supplementary table S9. Effect of allocation to empagliflozin on **costs of ESKD management**, overall and by subgroups at baseline (Limited to active-trial data) 27](#_Toc200462849)

[Supplementary table S10. Effect of allocation to empagliflozin on **costs of ESKD management**, overall and by subgroups at baseline (Limited to post-trial data) 28](#_Toc200462850)

[Supplementary table S11. Effects of 2 years treatment with empagliflozin on **QALYs and healthcare costs**, overall and by subgroups at baseline 29](#_Toc200462851)

[Supplementary figure S3. Effect of 2 years treatment with empagliflozin on h**ealthcare costs over 2 years** (ACTIVE-TRIAL) and **over 4 years** (further 2 years POST-TRIAL) by key subgroups 31](#_Toc200462852)

[Supplementary figure S4. Probability of 2 years treatment with empagliflozin being cost-effective over 2 years (ACTIVE-TRIAL) and 4 years (further 2 years POST-TRIAL) by key subgroups 32](#_Toc200462853)

[Supplementary table S12. Effect of allocation to empagliflozin on **rate of admissions**, overall and by MedDRA SOC (Limited to active-trial data) 33](#_Toc200462854)

[Supplementary table S13. Effect of allocation to empagliflozin on **days in hospital** overall and by MedDRA SOC (Limited to active-trial data) 34](#_Toc200462855)

[Supplementary table S14. Effect of allocation to empagliflozin on **hospital costs** overall and by MedDRA SOC (Limited to active-trial data) 35](#_Toc200462856)

[Supplementary table S15. Effect of 2 years treatment with empagliflozin on **days of and costs of ESKD**, overall and by type of ESKD management 36](#_Toc200462857)

[Supplementary figure S5. Effect of 2 years treatment with empagliflozin on **total healthcare costs over 4 years** (Years 3-4 ESKD costs only) in categories of CKD patients, at different empagliflozin cost 37](#_Toc200462858)

[Supplementary figure S6. Probability of 2 years treatment with empagliflozin being cost-effective over 4 years (2 years ACTIVE-TRIAL + further 2 years POST-TRIAL) in categories of CKD patients, at different empagliflozin cost 38](#_Toc200462859)

[Supplementary References 39](#_Toc200462860)

# Members of the EMPA-KIDNEY Collaborative Group

***Membership of the Executive Committee, Steering Committee and Independent Data Monitoring Committee***

*Executive Committee*

Colin Baigent (co-Chair), Martin J. Landray (co-Chair), Christoph Wanner (Deputy Chair), William G. Herrington (Chief Investigator), Richard Haynes (co-Principal Investigator), Jennifer B. Green, Sibylle J. Hauske*, Martina Brueckmann*, Mark Hopley*

(Previous members: Maximillian von-Eynatten* & Jyothis George*)

*Steering Committee*

Executive Committee members plus National Representatives: Susanne Brenner (Germany); Alfred K. Cheung (United States); David Preiss (United Kingdom); Zhi-Hong Liu, Jing Li (China); Laiseong Hooi, Wen Liu (Malaysia); Takashi Kadowaki, Masaomi Nangaku (Japan); Adeera Levin, David Cherney (Canada); Roberto Pontremoli, Aldo P. Maggioni (Italy); plus statistician members: Natalie Staplin, Jonathan Emberson, Stefan Hantel*; plus other expert members: Shinya Goto, Rajat Deo, Katherine R. Tuttle. Non-voting members: Michael Hill, Parminder Judge, Kaitlin J. Mayne, Sarah Y.A. Ng, Xavier Rossello, Emily Sammons, Doreen Zhu

* denotes a Boehringer Ingelheim employee

*Independent Data Monitoring Committee*

Peter Sandercock (Chair), Rudolf Bilous, Charles Herzog, Paul Whelton, Janet Wittes, Derrick Bennett (non-voting statistician)

***Central and Regional Coordination***

**Central Coordinating Office**

***Administration*:** Patricia Achiri, Chrissie Ambrose, Cristina Badin, Jill Barton, Richard Brown, Andy Burke, Sebastian Butler, Rejive Dayanandan, Pia Donaldson, Robert Dykas, Lucy Fletcher, Kate Frederick, Hannah Kingston, Mo Gray, Emily Harding, Akiko Hashimoto, Lyn Howie, Susan Hurley, Ryonfa Lee, Nik Luker, Kevin Murphy, Mariko Nakahara, John Nolan, Michelle Nunn, Sorcha Mulligan, Akiko Omata, Sandra Pickworth, YanRu Qiao, Shraddha Shah, Karen Taylor, Alison Timadjer, Monique Willett, Liz Wincott, Qin Yan, Hui Yu, Nichola Jones, Bridget Henderson, Genna Bobby; ***Clinical***: Louise Bowman, Fang Chen, Robert Clarke, Michelle Goonasekera, Richard Haynes, William G. Herrington, Parminder Judge, Waseem Karsan, Marion Mafham, Kaitlin J. Mayne, Sarah Y. A. Ng, David Preiss, Christina Reith, Emily Sammons, Mohammed Zayed, Doreen Zhu, Nikita Agrawal, Ryoki Arimoto; ***Data Analysis:*** Ritva Ellison, Rowan Moys, Will Stevens, Kevin Verdel, Karl Wallendszus; ***Finance:*** Chris Bowler, Anna Brewer, Andy Measor; ***IT Validation:*** Guanguo Cui, Charles Daniels, Angela Field, Bob Goodenough, Ashley Lawson, Youcef Mostefai, Dheeptha Radhakrishnan, Samee Syed, Shuang Xia; Laboratory: Ruth Adewuyi-Dalton, Thomas Arnold, Anne-Marie Beneat, Anoushka Bhatt, Chloe Bird, Andrew Breach, Laura Brown, Mark Caple, Tatyana Chavagnon, Karen Chung, Sarah Clark, Luminita Condurache, Katarzyna Eichstadt, Marta Espino Obrero, Scarlett Forest, Helen French, Nick Goodwin, Andrew Gordon, Joanne Gordon, Cat Guest, Tina Harding, Michael Hill, Michal Hozak, Matthew Lacey, David MacLean, Louise Messinger, Stewart Moffat, Martin Radley, Claire Shenton, Sarah Tipper, Jon Tyler, Lesley Weaving, James Wheeler, Elissa Williams, Tim Williams, Hamish Woodhouse; ***Monitoring:*** Angela Chamberlain, Jo Chambers, Joanne Davies, Denise Donaldson, Pati Faria-Shayler, Denise Fleming-Brown, Jennifer Ingell, Carol Knott, Anna Liew, Helen Lochhead, Juliette Meek, Isabel Rodriguez-Bachiller, Andrea Wilson, Patrick Zettergren, Meera Mistry; ***Programming:*** Rach AitSadi, Ian Barton, Alex Baxter, Yonghong Bu, Lukasz Danel, Sonja Grotjahn, Rijo Kurien, Michael Lay, Archie Maskill, Aleksandra Murawska, Rachel Raff, Allen Young; ***Principal Investigators:*** Colin Baigent, Richard Haynes, William G. Herrington, Martin J. Landray, David Preiss; ***Statistics:*** Jonathan Emberson, Rebecca Sardell, Natalie Staplin

**Regional Coordinating Centres**

**Germany (Universitätsklinikum Würzburg):** Christoph Wanner, Susanne Brenner, Vladimir Cejka, Marcela Fajardo-Moser, Christian Hartner, Doris Poehler, Janina Renner, Franziska Scheidemantel, Sharang Ghavampour

**United States (Duke Clinical Research Institute [DCRI])**: Jennifer B. Green, Miya Bryant, Anita Hepditch, Cassandra Johnson, Erin Latore, Yolanda Miller, Lauren Price, Merilee Whalen, Ashleigh Wheeler

**UK (Clinical Trial Service Unit and Epidemiological Studies Unit [CTSU], University of Oxford)**: Richard Haynes, David Preiss, Cristina Badin, Jo Chambers, Joanne Davies, Denise Donaldson, Mo Gray, Emily Harding, Jenny Ingell, Yanru Qiao, Shraddha Shah, Andrea Wilson, Patrick Zettergren

**China (National Center for Cardiovascular Disease, Fuwai Hospital & National Clinical Research**

**Center of Kidney Diseases, Jinling Hospital, Nanjing University School of Medicine):** Zhi-Hong Liu, Jing Li, Yu An, Yinghua Chen, Peiling Chen, Hao Dai, Hong Du, Fang Feng, Qing Guo, Libo Hou, Wuhanbilige Hundei, Binbin Jin, Yan Li, Jiamin Liu, Xia Song, Yanping Wang, Yanwu Yu, Ning Zhang, Lingshan Zhao, Hui Zhong, Yi Yang, Ying Sun

**Malaysia (Klinsel SDN BHD)**: Cheng Beng Goh, Ye Mun Low, Soon Yi Sor, Farah Hanis Zulkipli, Sarojini Sivanandam, Nurusyifaa’ Nadhirah Mohd Shahfari

**Japan (Parexel):** Natsuki Arai, Ai Fukasawa, Mizue Furukawa, Keisuke Habuki, Shoko Hayashi, Wakako Isari, Saki Kanegae, Maria Kawai, Reiki Kobayashi, Takako Kuramae, Chika Kuribayashi, Sawako Maeno, Satoshi Masumoto, Tomoko Morisaki, Minoru Oda, Kazue Sawada, Kenta Sugamori, Ayana Tatsuzawa, Aiko Tomita, Kazuyuki Yuasa, Hiroko Inazawa

**Canada (Providence Health Care, Vancouver):** Adeera Levin, Amanda Axler, Kerri Gallo

**Italy (ANMCO Research Center):** Aldo P. Maggioni, Ester Baldini, Barbara Bartolomei Mecatti, Francesca Bianchini, Martina Ceseri, Laura Cipressa, Gianna Fabbri, Andrea Lorimer, Donata Lucci

***List of Collaborators, by Site***

**Germany:** Universitatsklinikum Wurzburg: Christoph Wanner, Susanne Brenner, Vladimir Cejka, Sharang Ghavampour, Anja Knoppe, Tereza Cairns; Zentrum fuer Nieren-, Hochdruck- und Stoffwechselerkrankungen Hannover: Hans Schmidt-Gurtler, Hubert Dumann, Sybille Merscher, Margret Patecki, Georg Rainer Schlieper, Anke Torp, Bianca Weber, Maja Zietz; Nephrologisches Zentrum Villingen-Schwenningen: Bernd Hohenstein, Urs Benck, Diliana Draganova, Thomas Weinreich, Lothar Wolf, Jasmine Gaidu, Hanna Reiner, Mandy Visnjic; Nierenzentrum Freiburg: Daniel Steffl, Marie Breitenfeldt, Annette Kraemer-Guth, Christine Braun, Simone Hagge; Dialysezentrum Heilbronn: Michael Schomig, Stephan Matthias, Dominik Stoffler, Beate Schumacher; Klinikum der Universitat Munchen: Thomas Sitter, Louise Fuessl, Julia Krappe, Jerome Loutan, Volker Vielhauer, Luciano Andriaccio, Magdalena Maurer, Sybille Spies; ClinPhenomics GmbH Co. KG: Bernhard Winkelmann, Martin Dursch, Linda Seifert, Linda Tenbusch, Gudrun Schneckenburger, Tina Geinitz, Kerstin Michalek, Simon Steininger, Julia Mueller; Universitatsmedizin Mainz: Julia Weinmann-Menke, Simone Boedecker, Wiebke Kaluza-Schilling, Daniel Kraus, Carina Krieger, Margit Schmude, Anne Schreiber, Ewelina Eckrich; Herz- und Diabeteszentrum Nordrhein-Westfalen: Diethelm Tschope, Abdulwahab Arbi, Young Lee-Barkey, Bernd Stratmann, Natalie Prib, Sina Rolfsmeier, Irina Schneider; Universitatsklinikum Dusseldorf: Lars Rump, Johannes Stegbauer, Christine Pötz, Mara Schemmelmann, Claudia Schmidt, Sinje Landmann; Nephrocare Mettmann - Standort Velbert: Michael Koch, Sendogan Aker, Annika Küpper, Manuela Martin; Diaverum MVZ Potsdam: Thiemo Pfab, Christian Albert, Michael Haase, Barbara Zander, Claudia Schneider-Danwitz; Praxis fur Dialyse und Nierenkrankheiten - Arztezentrum Helle Mitte: Wolfgang Seeger, Wolf-Adam Seeger, Britta Zemann; Klinikum Bielefeld: Christoph Stellbrink, Kristin Marx, Ekaterina Stellbrink, Britta Brettschneider, Stephanie Watson, Marion Iselt; Studienzentrum Aschaffenburg: Gerhard Klausmann, Inga-Nadine Kummer, Auguste Kutschat, Simone Streitenberger; Universitatsklinikum Halle: Matthias Girndt, Silke Markau, Ina Girakossyan, Claudia Hanf; Klinikum St. Georg Leipzig: Joachim Beige, Ralph Wendt, Ulrike Schmidt, Birgit Labitzke, Leoni Leistner; Studienzentrum Nephrologie Nurnberg-Langwasser: Andreas Schneider, Roland Veelken, Claudia Donhauser, Auguste Kutschat; UBAG fur Nephrologie und Dialyse Neckarsulm: Luis Becker, Nexhat Miftari, Ricarda Wolfling, Sarah Morlok; Universitatsklinikum Dresden: Christian Hugo, Alexander Paliege, Jens Passauer, Julian Stumpf, Annegret Fleischer, Kerstin Haaser; Universitatsklinikum Mannheim: Bernhard Kraemer, Jan Jochims, Bernd Kruger, Claudia Foellinger, Anastassiya Reisler; Nierenzentrum Wiesbaden: Frank Strutz, Stefan Haack, Ursula Hohenstatt; Universitatsklinikum Jena: Martin Busch, Konstantin Herfurth, Gunter Wolf, Rainer Paul, Andy Steiner; Studienzentrum fur Nieren- und Hochdruckerkrankungen Hannover: Hermann Haller, Jessica Kaufeld, Jan Menne, Elisabeth Bahlmann-Kroll, Angela Bergner, Kai Schmidt-Ott; Universitatsklinikum Augsburg: Horst Weihprecht, Aydin Er, Florian Sonntag, Elif Turan, Michael Wittmann, Franziska Klauser, Eva Voigt, Julia Gatzschmann, Franziska Thieme; Nephrologisches Zentrum Gottingen: Volker Schettler, Egbert Schulz, Madlen Rohnstock, Elke Schettler; Universitaetsklinik Ulm: Bernd Schroppel, Rene van Erp, Martin Kachele, Ulla Ludwig, Lena Schulte-Kemna, Waltraud Kmietschak, Elke Preiss, Martina Ruocco; AGAPLESION Markus-Krankenhaus: Gunnar Heine, Martin Brzoska, Sebastian Gabel, Christina Büttner, Asma Sabarai, Christina Buttner; Universitatsklinikum Regensburg: Bernhard Banas, Tobias Bergler, Yvonne Ehrl, Franz Putz, Antonia Schuster, Stefanie Kuhn, Torsten Schramm; DaVita Viersen - Nettetal: Stefan Degenhardt, Gerhard Schmidt, Lea Weiland, Ulrike GiebelnHudnell; Klinikum Braunschweig: Jan Kielstein, Gabriele Eden, Brigitte Fuchs, Gina Morig, Manuela Winkler, Christina Engel; Nephrocare Mettmann: Michael Koch, Sendogan Aker, Annika Küpper, Manuela Martin; Vivantes Klinikum Neukolln: Harald Darius, Charalampos Kriatselis, Carl- Philipp Roesch; Astrid Maselli, Robert-Bosch-Krankenhaus Stuttgart: Dominik Alscher, Markus Ketteler, Moritz Schanz, Severin Schricker, Bianka Rettenmaier, Andrea Schwab, Fateme Rahimi

**United States:** Clinical Advancement Center: Pablo Pergola, Irene Leal, Melissa Cagle, Anna Romo, Anthony Torres, Natalia Cabrera; Seacoast Kidney and Hypertension Specialists: Sucharit Joshi, Kulli Barrett, Alexis Africano, Vicki Dodds, Dorleena Gowen, Ashlee Morris, Stacey Perry; Total Research Group, LLC: Juan Fernandez, Guillermo Jimenez, Ricardo Viera, Kendaling Bruce, Ryan Barrios, Maylin Garcia, Kerelyn Garcia, Iradis Leal; Nephrology Consultants, LLC: David Tietjen, David Bains, Carlo Castillo, Genielle Brewer, Justin Davis, Natalie Freking, Brittany Golson, Sally Ham, Jesslyn Roesch; Sumter Medical Specialists: Pusadee Suchinda, Shameem Beigh, Usah Lilavivat, Joyce Bilton, Kim Bocchicchia, Heidi Griswold; Yale University: Jeffrey Turner, Neera Dahl, Aldo Peixoto, Yasemin Kavak, Lauren Liberti, Hari Nair, Nicolas Page, Stephanie Rosenberg, Kathryn Simmons; Northwestern University: Tamara Isakova, Rebecca Frazier, Rupal Mehta, Anand Srivastava, Patrick Fox, Jonathan Hecktman, Alexander Hodakowski, Carlos Martinez, Rachel Phillips, Alexis Stevenson, Marija Zimkute, Reed Jaworski; University of Kansas Medical Center: Reem Mustafa, Kyle Jansson, Cassandra Kimber, Jason Stubbs, Ahmad Tuffaha, Sri Yarlagadda, Debbie Griffin, Elisabeth Laundy, Zhuo Tang, Casey Tan, Abigayle Joyce; Providence Sacred Heart Medical Center and Childrens Hospital: Radica Alicic, Katherine R. Tuttle, Ann Cooper, Lisa Davis; East Coast Research Institute: Ashwini Gore, Rebecca Goldfaden, Leslie Harvill, Lisa Hichkad, Barry Johns, Thomas Jones, Kayla Merritt, Jennifer Sheldon, Jennifer Stanfield, Lindsay Alexander, Kaitlyn Preston, Lindsey Wood; Monument Health: Rajesh Pradhan, Roger DeRaad, Kelli McIntosh, Louis Raymond, Michael Shepperd, Susan McLaughlin, Mary Seifert, Andrew Shepherd; Mountain Kidney & Hypertension Associates: Joseph Aiello, William Durham, Laurie Loudermilk, John Manley, Sabrina Burnette, Stephanie Evans, Tara Johnson; Texas Institute for Kidney and Endocrine Disorders: Lance Sloan, Judy Ann Acosta, Stacy Gillham, Katia Sloan, SueAnn Squyres; Wake Forest University Health Sciences: Michael Rocco, Amret Hawfield, Ben Bagwell, Lauren Richmond; Chase Medical Research: Joseph Soufer, Subha Clarke, Amanda Aliu, Kristine Calabrese, Amanda Davis, Veronica Poma, Tracy Spinola; East Coast Institute for Research LLC: James Magee, Ricardo Silva, Rushab Choksi, Lorraine Dajani, John Evans, Anil George, Rebecca Goldfaden, Prasanth Krish, Gerard Martins, Mae Sheikh-Ali, David Sutton, Freda Driver, Abraham Hanburry, Laura Hume, Amber Hurst, Matthew Taddeo, Marla Turner, Veronica Yousif; University of Utah Health Sciences: Srinivasan Beddhu, Laith Al-Rabadi, Nikita Abraham, Amalia Caamano, Judy Carle, Victoria Gonce, Kaitlyn Staylor, Na Zhou; University of Texas Health Science Center at San Antonio: Shweta Bansal, Manoj Bhattarai, Kumar Sharma, Subrata Debnath, Aliseiya Garza, Chakradhar Velagapudi; Academy of Diabetes, Thyroid, and Endocrine, PA: Sergio Rovner, Javier Almeida, Pablo Casares, Verlaine Stewart-Ray, Rene Almaraz, Renata Dayrell, Ana Moncada, Ricardo Pulido, Roxana Rodriquez; East Coast Institute for Research: James Magee, Wasim Deeb, Kathryn DeGoursey, Rodel Gloria, Trevor Greene, Robert Miller, Edward Pereira, Miguel Roura, Mae Sheikh-Ali, David Sutton, Debbie Domingo, Sasha Dorestin, William Hodge, Cathy Jackson, Deborah Lund, Katrina Taylor; Aventiv Research: Kenneth Boren, Brittany Cleveland, Sandra Gaiser, Mandeep Sahani, Logan Aldrich, Exodus Edmerson, Edmond Limon, Cole Valletta, Patricia Vasquez, Amanda Harrington, Haley Edwards, Jennifer Green; St. Clair Nephrology Research: Christopher Provenzano, Navkiranjot Brar, Heather Henderson, Bellovich Keith, Qur Khai, Quresh Khairullah, Gail Makos, Joel Topf, Sherry Gasko, Rosemarie Henschel, Kaitlin Knapp, Teresa Kozlowski, Paula LaFleur, Ashwathy Varughese; Kaiser Permanente San Diego: Hui Xue, Patricia Wu, Olga Arechiga, Shan Darbeau, Michael Fechter, Stephanie Martinez, Katherine Klein, Eva Rodriguez; Hanson Clinical Research Center: Lenita Hanson, Nyla Cooper, Arelis Madera, Jay Cadorna, Rita Sheridan, Helen Sparks; Saint Elizabeth Healthcare: Bradley Eilerman, Susanne Bodine, Wael Eid, Rebecca Flora, Amber Avery, Cashmere Hardy; Thomas Jefferson/ARIA Health Northeast Endocrine Metabolic Associates: Mihaela Biscoveanu, Steven Nagelberg, Tracey Cummins; Emory University: Frederic Rahbari-Oskoui, Anju Oommen, Zohreh Forghani, Stacie Hitchcock, Darya Hosein, Diane Watkins; East Coast Institute Research, LLC: Minesh Patel, Anthony Lambert, Elizabeth Newman, Autumn Wood, Tammy Ross, Stephany Topping; Kidney Care and Transplant Services of New England: Jeffrey Mulhern, Lorna Murphy, Ann Vasseur; Brookview Hills Research Associates LLC: Gregory Greenwood, Alexander Hadley, Denise Laurienti, Christopher Marshall, Nicholas McLean, Scott Satko, Brandy Caudill, Jacob Maris, Janice Rogers, Cindy Vanhoy; Cleveland Clinic: George Thomas, Georges Nakhoul, John O'Toole, Jonathan Taliercio, Leslie Cooperman, Marina Markovic, Barbara Tucky; Salem V.A. Medical Center: Devasmita Dev, Alia Hasan, Hima Yalamanchili, Namita Jain, Lesley McNeil, Eric Wines; Medstar Health Research Institute: Jean Park, Adline Ghazi, Mia Hamm, Tejas Patel; University of North Carolina Hospital: Amy Mottl, Emily Chang, Vimal Derebail, Emmie Cole, Anne Froment, Sara Kelley, Jordan Osmond Foster; Olive View - UCLA Medical Center: Vahid Mahabadi, Golriz Jafari, Anita Kamarzarian, Wendy Arriaga, Daisy Arteaga, Rosario Machicado, Genesis Naverrete; P&I Clinical Research, LLC: Prashant Kumar, Imran Nazeer, Karina Urquia, Tammi Glider, Vickie Jones, Savannah Rucker, Jennifer Wiley, Tammy Rider; Pioneer Research Solutions: Rahul Pandey, Jesus Arroyo, Harish Pariani, Mohammad Ahmad, Shahin Mozaffari, Erika Perez, Andres Miranda; Los Angeles Biomedical Research Institute at Harbor-UCLA Medical Center: Matthew Budoff, Sion Roy, Divya Birudaraju, Ahmed Ghanem, Sajad Hamal; Research Institute of Dallas: Stephen Aronoff, Elisa Joye Petr, Richard Sachson, Jaime Wiebel, Sana Akram, Laurie Jones, Curtis Knight; Maurie Tarlac, Idara Ukpong, Kim Quiroga; Renal Disease Research Institute: Shahbaz Ahmed, Harold Szerlip, Akinwande Akinfolarin, Ankit Mehta, Shana Camp, Cindy Castro, Zanaida Cooper, Jessica Terry; Clinical Research Consultants: Ahmed Awad, Bhavya Kothapalli, Ryan Lustig, Serine Alfaress, Hyder Jasim, Mary Parrigon; Lexington V.A. Health Care System: Dennis Karounos, Sadiq Ahmed, Maggie Berry, Ruth Oremus; VA Southern Nevada Healthcare System: Carlos Hernandez-Cassis, Elias Ugwu, Nazia Junejo, Nancy Suazo, Todd Clark, Rosalinda Cruz; University of Florida Health: Mark Segal, Amir Kazory, Sherry Brown, Tristan Daniels, Sofia Dayi, Renee Hogan, Kathy McCray, Jennifer Stickley; University Hospitals Cleveland Medical Center: Mahboob Rahman, Mirela Dobre, Lavinia Negrea, Aparna Padiyar, Nishigandha Pradhan, Arash Rashidi, Nagaraju Sarabu, Vicki Donley, Tricia Young, Elizabeth DeCaro; Midland Florida Clinical Research Center: Godson Oguchi, Judepatricks Onyema, Kahla Damianik, Jack Dienes, Judith Plummer-Morgan, Marilyn Roman, Mauver Skipper, Stacey-Ann Villaruel, Krystle Williams, Svetlana Shilo, Numaliz Chokr; Cedar Crosse Research Center: Danny Sugimoto, Jeffrey Dugas, Ismeal Ahmed, Jamie Bhairoo, Dolores Rijos, Huzaifa Salim, Kaleena Urquidi

**UK:** Oxford University Hospitals: Richard Haynes, William G. Herrington, Doreen Zhu, Madita Gavrila, Kathryn Lafferty, Ria Rabara, Sally Ruse, Maria Weetman; Southmead Hospital, Bristol: James Bushnell, Albert Power, Alison Jenkins, Stefanie Jones, Amanda Scott; Nottingham City Hospital: Cath Byrne, Mark Jesky, Alison Cowley, Emma McHaffie, Holly Waterfall, Neha Bhalla; Dorset County Hospital: Jo Taylor, Laura Bough, Thomas Phillips, Barbara Winter-Goodwin, Keegan Lee; King's College Hospital, London: Sui Phin Kon, Iain MacDougall, Eirini Lioudaki, Sapna Shah, Claire Sharpe, Francisco Aguilar, Abegail Hernandez Pena, Conception Pugay, Amelia Te, Tony Johny, Philip Francisco; Queen Elizabeth Hospital Birmingham: Hugh Finn, Wasim Hanif, Samiul Mostafa, Alice Aitken, Katharine Draxlbauer, Evelina Grobovaite, Jennifer Kearney, Theresa McCarthy, Faye Moore, Christianah Morakinyo, Sephora Thorpe; Royal Cornwall Hospital: Giorgio Gentile, Duncan Browne, Palanichamy Chellamuthu, Tabinda Dugal, Terri Chant, Laura Jones, Emily Laity, Megan Miners, James Muir, Elizabeth Swanson; Imperial College Healthcare NHS Trust: Andrew Frankel, James Tomlinson, Marlon Alegata, Rashid Almasarwah, Anthoula Apostolidi, Maria Vourvou, Thomas Walters; Royal Derby Hospital: Maarten Taal, Hari Dukka, Nitin Kolhe, Carly McDonald, Kelly White; The Queen Elizabeth Hospital, King's Lynn: Shiva Ugni, Smita Gunda, Rotimi Oluyombo, Vicki Brindle, Ping Coutts, Tracy Fuller, Evelyn Nadar; Princess Royal Hospital, Telford: Suresh Ramadoss, Denise Donaldson, Nichola Motherwell, Susannah Pajak, Louise Tonks, Mandy Beekes; Hull Royal Infirmary: Sunil Bhandari, Richard Bodington, Adil Hazara, Dominic Fellowes; University Hospital Aintree: Christopher Wong, Christopher Goldsmith, Sherald Barnes, Ann Bennett, Claire Burston, Samantha Hope, Nicola Hunt, Lini Kurian; UHNM Royal Stoke University Hospital: Richard Fish, Daniela Farrugia, Judy Lee, Emma Sadler, Hannah Turner; Belfast City Hospital: Christopher Hill, Henry Brown, Agnes Masengu, Peter Maxwell, Nina Bleakley, Hugh Murtagh; West Suffolk NHS Foundation Trust: William Petchey, Vivian Yiu, Joanne Kellett, Angharad Williams, Veronica Mendez Morro; Royal Devon and Exeter Hospital: Helen Clarke, Victoria Carnall, Sarah Benyon, Caroline Blake, Stephanie Estcourt, Jane Piper, Gigee Joseph; Daisy Hill Hospital: Neal Morgan, Carolyn Hutchinson, Teresa McKinley; Ulster Hospital, Dundonald: Alastair Woodman, Judi Graham, Niall Leonard, John Smyth, Vicki Adell, Samantha Hagan; Royal Free London NHS Foundation Trust: Ben Caplin, Amin Oomatia, Eleanor Damian, Toluleyi Sobande, Phil Gardiner; Kent & Canterbury Hospital: Tim Doulton, Michael Delaney, Mahmoud Montasser, Jenny Hansen, David Loader, Angela Moon, Frances Morris; Salford Royal NHS Foundation Trust: Smeeta Sinha, Chukwuma Chukwu, Amy Hudson, Diane Campbell, Melanie Kershaw, Stephanie Whittaker, Katarzyna Adeniji; Brighton and Sussex University Hospital's NHS Trust: Ayesha Irtiza-Ali, Farid Ghalli, Heba Nosseir, Allison Leslie, Kate Trivedi; University Hospital of Wales, Cardiff: Donald Fraser, Mohammad Alhadj Ali, Sian Griffin, Farah Latif, Justyna Witczak, Alexa Wonnacott, Lynda Jeffers, Yvette Webley; Edinburgh Royal Infirmary: Paul Phelan, Eve Miller-Hodges, Ailsa Geddes, Margaret Glenwright, Amy Hunter; Gloucestershire Hospitals NHS Foundation Trust: Thomas Pickett, Jim Moriarty, Linda Hill, Amanda Tyler; University Hospitals Coventry and Warwickshire: Waqar Ayub, Gail Evans, Sue Hewins, Davina Hewitt, Kerry Read; Ninewells Hospital: Samira Bell, Leanne Cosgrove, Rachel Craik, Shona Murray; Royal Berkshire Hospital, Reading: Nitin Bhandary, Holly Coles, Rashmi Easow, Maya Joseph, Deepa Thapa; Northern General Hospital, Sheffield: Arif Khwaja, Yvonne Jackson, Angeline Mbuyisa, Rachel Sellars, Sadaf Younis, Kimiko Chapman; Darent Valley Hospital, Dartford: Nihil Chitalia, Cynthia Mohandas, Anca Gherman, Charlotte Kamundi, Olumide Olufuwa, Ryan Coe; Royal London Hospital: Kieran McCafferty, Adedolapo Adeleke, Cara Healy, Damini Jeyarajah, Edward Kinsella-Perks; Ipswich Hospital: Richard Smith, Brian Camilleri, Carol Buckman, Jenny Finch, Vanessa Rivers; University Hospitals Plymouth NHS Trust: Andrew Connor, Sheila Carr, Lisa Shainberg; Cheltenham General Hospital: Thomas Pickett, Linda Hill, Amanda Tyler; St. James's University Hospital, Leeds: Andrew Lewington, Richard Baker, Suzannah Dorey, Kay Tobin, Rosalyn Wheatley; St. George's University Hospitals NHS Foundation Trust: Debasish Banerjee, Richard Hull, Sharirose Abat, Riny Paul; Norfolk and Norwich University Hospitals: Mahzuz Karim, Zay Htet, Rotimi Oluyombo, Saad Tufail, Ravi Varma, Karen Convery, Deirdre Fottrell-Gould, Lisa Hudig, Emily Tropman, Jane Platt; Walsall Healthcare NHS Trust: Thahir Abdul-Samad, Anne Grace, Marie Phipps, Gemma Highway; St Helier Hospital, Carshalton: Rebecca Suckling, Subash Somalanka, Bhrigu Sood, Pauline Swift, Sarah Acheampong, Kwame Ansu, Martia Augustin; Wessex Kidney Centre, Queen Alexandra Hospital, Portsmouth: Anna Sampson, Lynn Vinall, Kim Wren; St Bartholomew's Medical Centre: Shamila Wanninayake, Nicholas Wooding, Heather Edwards, Lydia Owen; Antrim Area Hospital: Stephanie Bolton, Marion Carson, Michael Matthews; University Hospitals of Leicester: Nigel Brunskill, Jorge Jesus-Silva, Alex Howson, Mary Quashie-Akponeware, April Maria Murillo; North Middlesex University Trust Diabetes Department, North Middlesex University Hospital: Hilary Tindall, Chidambaram Nethaji, Helen Eldon; Glasgow Clinical Research Facility, Queen Elizabeth University Hospital: Rajan Patel, Patrick Mark, Alastair Rankin, Michael Sullivan, Kirsty Forsyth, Rowan McDougall; Great Western Hospital, Swindon: Tanaji Dasgupta, Louisa Davies, Maggie Ryder, Suzannah Pegler; Hathaway Medical Centre, Chippenham: Philip Grimmer, Clare Macdonald, Mary Webster; Newcastle: Timothy Ellam, Edwin Wong, Christine Meshykhi, Andrea Webster, Peter Wilson; Lister Hospital: Enric Vilar, Jocelyn Berdeprado, Eunice Doctolero, Lily Wilkinson; Altnagelvin Hospital, Western Health & Social Care Trust: Frank McCarroll, Hesham Ammar, Ying Kuan, Conor Moran, Girish Shivashankar, Ryan Campbell, Deborah Glowski, Paula McDermott; Oakenhurst Medical Practice, Blackburn: Amar Ali, Zuber Patel, Christine Bond, Gillian Whalley

**China:** National Clinical Research Center of Kidney Diseases, Jinling Hospital, Nanjing University School of Medicine: Haitao Zhang, Peiling Chen, Yu An, Yinghua Chen, Liu Yang, Lihua Zhang, Tingting Kan, Ling Zhu; The Second Affiliated Hospital of Army Medical University, PLA: Jinghong Zhao, Weiping Hou, Jing Wu; Beijing Anzhen Hospital, Capital Medical University: Hong Cheng, Weijing Bian, Zhirui Zhao; Henan Provincial People's Hospital: Fengmin Shao, Huixia Cao, Xiaojing Jiao, Peiyuan Niu; Shanghai Fifth People's Hospital, Fudan University: Jianying Niu, Yu Chen, Lihong Zhang; Huazhong University of Science and Technology Union Shenzhen Hospital: Shenglang Zhu, Haiyan Lin, Shaopeng Yao, Jiehui Chen, Ying Jiang; The second affiliated hospital of Zhejiang University School of Medicine: Ying Hu, Huaying Xiao, Fuye Yang; Shenzhen People's Hospital: Xinzhou Zhang, Baochun Guo, Qiu Jin, Lixia Liu; Xiangya Hospital, Central South University: Xiangcheng Xiao, Yanyun Xie, Ting Meng; Wuhan Fourth Hospital: Chuanwen Xu, Jie Huang, Yanmei Xu; Suzhou Kowloon Hospital: Weixin Kong, Xiaoliang Wang, Qianpan Liu,; Jinzhou Central Hospital: Xueying Wang, Ming Gao; Zhuzhou Central Hospital (Nephrology): Xiumei Hu, Ying Lu; Sichuan Provincial People's Hospital: Li Wang, Kun Peng, Wei Wang; Fuwai Hospital, Chinese Academy of Medical Sciences: Qiuhong Gong, Jianfang Cai, Xiaojue Li, Xuejiao Liu, Haitao Zhang, Shuhan Zhou; Zhuzhou Central Hospital (Endocrinology): Hong Liu, Yao Weng, Shuai Tang, Yao Yao; The Central Hospital of Wuhan: Shi Zhao, Chen Cheng, Wei Wei, Na Li

**Malaysia:** Hospital Kajang: Sadanah Aqashiah Mazlan, Alia Zubaidah Bahtar, Elliyyin Katiman, Noraini Othman; Hospital Tuanku Ja'afar: Lily Mushahar, Nurdiana Mazlan, Nur Sharafina Safiee, Sarasa Ramasamy; Hospital Selayang: Hin Seng Wong, Hajar Ahmad Rosdi, Esther Zhao Zhi Tan, Ju Fan Tay; Hospital Taiping: Kok Seng Teng, Hasnah Yahaya; Hospital Sultanah Aminah: Wen Jiun Liu, Lik Wee Ee, Kenneth Kay Leong Khoo, Yuana Mohd Yusoff; Hospital Tengku Ampuan Afzan: Fariz Safhan Mohamad Nor, Mohd Kamil Ahmad, Mohd Ramli Seman; Hospital Umum Sarawak: Clare Hui Hong Tan, Laura Lui Sian Ngu, Jaime Yoke May Chan, Javelin Peji; Hospital Raja Permaisuri Bainun: Chek Loong Loh, Yee Yan Lee, Sridhar Ramanaidu, Kah Mean Thong, Yik Hong Wong, Suria Junus; Hospital Sultanah Bahiyah: Chen Hua Ching, Mohammad Faisal Asmee, Ku Ruziana Ku Md Razi, Chun Leong Low, Christopher Sze Bing Sim, Zhang Duan Tham, Noor Kamila Abdullah; Hospital Sultan Abdul Halim: Tai Meng Chen, Yong Chieh Chan, Eason Chang, Huan Yean Kang, Kai Quan Lee, Sue Ann Lee, Aik Kheng Lee, Jeevika Vinathan, Chyi Shyang Tan; Universiti Kebangsaan Malaysia Medical Centre: Rizna Abdul Cader, Ruslinda Mustafar, Lydia Kamaruzaman, Rozita Mohd, Rahimah Ismail; Hospital Kulim: Chong Men Leong, Chee Koon Low, Liang Wei Wong, Yik Shen Lim, Norlezah Adnan, Sabariah Ibrahim; Hospital Kuala Lumpur: Mohamad Zaimi Abdul Wahab, Sunita Bavanandan, Yik Shen Lim, Zhang Duan Tham, Wan Hazlina Wan Mohamad, Siti Munirah Jaafar, Nur Ashykeen Mohd Fauzi, Aziee Sudin; University Malaya Medical Centre: Soo Kun Lim, Chye Chung Gan, Albert Hing, Wan Ahmad Faizal Alaidin Razali; Hospital Pulau Pinang: Yew Fong Liew, Chelsia Bao Tyng Chan, Mei Chih Cheng, Yu Chen Ong, Loke Meng Ong, Farah Amalina Mohamed Affandi; Hospital Melaka: Korina Rahmat, Ban Chai Peng, Masayu Amat; Hospital Pakar Sultanah Fatimah: Nuzaimin Hadafi Ahmad, Doo Yee Mah, Yi Loon Tye, Zaid Azhari, Siti Nabilah Mohamad Zaini, Mohd Aidil Musa, Nur Nadzifah Hanim Zainal Abidin, Zher Lin Go; Hospital Ampang: Norazinizah Ahmad Miswan, Rafizanur Ramli, Nor Aziah Ahmad; Hospital Serdang: Bak Leong Goh, Nurul Izah Ahmad, Fairol Huda Ibrahim, Tze Jian Ng, Malini Shanmuganathan, Li Lian Tay; Hospital Sultanah Nur Zahirah: Zaiha Harun, Salmi Ramli, Nurul 'Ain Yusof, Rossenizal Abd Rahman; Pusat Perubatan UiTM: Muhammad Iqbal Abdul Hafidz, Nur Hidayati Mohd Sharif, Irda Yasmoon Awang

**Japan:** Chubu Rosai Hospital: Eitaro Nakashima, Rui Imamine, Makiko Minatoguchi, Yukari Miura, Miduki Nakaoka, Yoshiki Suzuki, Hitomi Yoshikawa; Shin Clinic: Koki Shin, Kanae Fujita, Misuzu Iwasa, Haruka Sasajima, Airi Sato; Kansai Electric Power Hospital: Yoshiyuki Hamamoto, Yuki Fujita, Takuya Haraguchi, Takanori Hyo, Kiyohiro Izumi, Toshiyuki Komiya, Sodai Kubota, Takeshi Kurose, Hitoshi Kuwata, Susumu Nakatani, Kaori Oishi, Saki Okamoto, Kaori Okamura, Jun Takeoka, Nagaaki Tanaka, Katsuya Tanigaki, Naohiro Toda, Koin Watanabe, Hiromi Komori, Rika Kumuji, Asako Takesada, Aya Tanaka; Nagoya University Hospital: Shoichi Maruyama, Tomonori Hasegawa, Akiko Ishiguro, Takuji Ishimoto, Kazuhiro Ito, Yutaka Kamimura, Noritoshi Kato, Sawako Kato, Hiroshi Kojima, Tomoki Kosugi, Kayaho Maeda, Masasi Mizuno, Shoji Saito, Hitomi Sato, Yuka Sato, Yasuhiro Suzuki, Akihito Tanaka, Yoshinari Yasuda, Fujiko Hasegawa, Maiko Hayashi, Shizuka Higashi, Kaho Shimamura, Momoko Sumi, Kazuki Tajima, Chimaki Unekawa, Kana Wakayama, Yukiko Wakita; Ota diabetes clinic: Takatoshi Otani, Ayako Imai, Sayaka Kawashima, Eri Kogure, Tomoe Sato, Misato Takezawa, Shinya Yoshida; Fukui Prefectural Hospital: Hideo Araki, Yuko Katsuda, Masahiro Konishi, Takahiro Matsunaga, Masashi Oe, Kunihiro Ogane, Masato Sakai, Tomoko Takahashi, Takahiro Yamano, Takuya Yokoyama, Hitomi Ito, Masayo Katayama, Emi Kuroda; Medical Corporation Seijinkai Ikeda Hospital: Toru Ikeda, Takuma Kojo, Etsuo Yoshidome, Rieko Mizumachi, Akane Yamamoto, Narihisa Yamasaki, Yoshihiko Yamasaki; Okayama University Hospital: Jun Wada, Jun Eguchi, Chigusa Higuchi, Akihiro Katayama, Masaru Kinomura, Masashi Kitagawa, Shinji Kitamura, Satoshi Miyamoto, Hiroshi Morinaga, Atsuko Nakatsuka, Ichiro Nojima, Kenichi Shikata, Hitoshi Sugiyama, Katsuyuki Tanabe, Kenji Tsuji, Haruhito Uchida, Mayu Watanabe, Chie Hashimoto, Takahiro Kato, Sayaka Yamamoto; Tokai University Hospital: Takehiko Wada, Masafumi Fukagawa, Naoto Hamano, Masahiro Koizumi, Hirotaka Komaba, Yosuke Nakagawa, Michiyo Iwamoto; Fukuoka University Hospital: Kosuke Masutani, Akane Katanosaka, Mayu Kiyota, Hikari Uchi, Yuka Ueda, Sonoka Yamamoto; Kawasaki Medical School Hospital: Hajime Nagasu, Seiji Itano, Tsukasa Iwakura, Hiroyuki Kadoya, Eiichiro Kanda, Naoki Kashihara, Kengo Kidokoro, Megumi Kondo, Tamaki Sasaki, Minoru Satoh, Atsuyuki Tokuyama, Reina Umeno, Yoshihisa Wada, Toshiya Yamamoto, Yu Yamanouchi, Masumi Abe, Yoko Inukai; Kobe University Hospital: Wataru Ogawa, Shunichiro Asahara, Hideki Fujii, Shunsuke Goto, Yushi Hirota, Tetsuya Hosooka, Keiji Kono, Shinichi Nishi, Yuko Okada, Kazuhiko Sakaguchi, Kenji Sugawara, Michiko Takahashi, Tomoko Takai, Yoshikazu Tamori, Kentaro Watanabe, Miyu Kitajima, Misaki Nishi, Junko Wada; Aichi Medical University Hospital: Yasuhiko Ito, Hideki Kamiya, Akimasa Asai, Nao Asai, Saeko Asano, Shogo Banno, Yohei Ejima, Hanako Hase, Tomohide Hayami, Tatsuhito Himeno, Takahiro Ishikawa, Mayumi Ito, Shiho Iwagaitsu, Rina Kasagi, Yoshiro Kato, Makoto Kato, Koichi Kato, Takayuki Katsuno, Miyuka Kawai, Hiroshi Kinashi, Masaki Kondo, Masako Koshino, Naoya Matsuoka, Yoshiaki Morishita, Mikio Motegi, Jiro Nakamura, Hiromi Shimoda, Hirokazu Sugiyama, Shin Tsunekawa, Makoto Yamaguchi, Kazuyo Takahashi; Juntendo University Hospital: Hirotaka Watada, Takashi Funayama, Yasuhiko Furukawa, Tomohito Gohda, Hiromasa Goto, Hideyoshi Kaga, Yasuhiko Kanaguchi, Akio Kanazawa, Kayo Kaneko, Toshiki Kano, Masao Kihara, Shogo Kimura, Takashi Kobayashi, Masayuki Maiguma, Yuko Makita, Satoshi Mano, Tomoya Mita, Takeshi Miyatsuka, Maki Murakoshi, Masahiro Muto, Masami Nakata, Junichiro Nakata, Yuya Nishida, Nao Nohara, Takeshi Ogihara, Daisuke Sato, Junko Sato, Hiroaki Sato, Yusuke Suzuki, Ruka Suzuki, Hitoshi Suzuki, Miyuki Takagi, Yoshifumi Tamura, Toyoyoshi Uchida, Seiji Ueda, Miki Asawa, Minako Miyaji, Eri Nagashima, Yoshie Shibata, Eri Yanagisawa; The University of Tokyo School of Medicine/Toranomon Hospital: Takashi Kadowaki, Toshimasa Yamauchi, Masaomi Nangaku, Yosuke Hirakawa, Hiroshi Nishi, Nobuhiro Shojima, Satoko Horikawa, Yukiko Nakayama, Naoko Yamada, Yuki Omori; Maebashi Hirosegawa Clinic: Shintaro Yano, Miyabi Ioka, Nahoko Kuwabara, Remi Nagano, Megumi Nozawa, Yumi Osawa; Shiga University of Medical Science Hospital: Hiroshi Maegawa, Shinji Kume, Shinichi Araki, Itsuko Miyazawa, Katsutaro Morino, Ikuko Kawai, Masumi Sobata, Motoko Takaoka; Koukan Clinic: Yasushi Iwaita, Takashi Udagawa, Ami Inamori, Aya Kawase, Aya Yamanaka; University of Tsukuba Hospital: Hitoshi Shimano, Akiko Fujita, Hitoshi Iwasaki, Hirayasu Kai, Yoshinori Osaki, Chie Saito, Motohiro Sekiya, Ryoya Tsunoda, Kunihiro Yamagata, Rikako Nakamura, Aiko Yamada; Center Hospital of the National Center for Global Health and Medicine: Mitsuru Ohsugi, Motoharu Awazawa, Ryotaro Bouchi, Shota Hashimoto, Makiko Hashimoto, Tomoko Hisatake, Noriko Ihana, Koko Ishizuka, Kazuo Izumi, Hiroshi Kajio, Michi Kobayashi, Noriko Kodani, Koji Maruyama, Michihiro Matsumoto, Maya Matsushita, Tomoka Nakamura, Takehiro Sugiyama, Akiyo Tanabe, Aiko Terakawa, Kojiro Ueki, Yuko Orimo, Takako Ozawa, Eriko Takahira; AMC Nishi-Umeda Clinic: Yoshimitsu Yamasaki, Masakazu Haneda, Tadahiro Tomita, Saori Akimoto, Akihiro Fujimoto, Kenji Ishihara, Chiho Murakami, Akiyo Nishiyama, Yukiko Toyonaga, Kana Uozumi, Yukihiro Yamaji; Jyoumou Ohashi Clinic: Tetsuya Shigehara, Jun Okajyo, Yukihiro Shimizu; Iwasaki internal medicine clinic: Shingo Iwasaki, Yuki Fukao, Megumi Furusho, Shintaro Nunokawa; Tohoku University Hospital: Hideki Katagiri, Tomohito Izumi, Keizo Kaneko, Shinjiro Kodama, Mariko Miyazaki, Yuichiro Munakata, Tasuku Nagasawa, Yuji Oe, Hiroto Sugawara, Kei Takahashi, Kazushige Hirata, Keiko Inomata, Shoko Otomo, Taeko Uchida, Chigusa Yamashita; Tokyo-eki Center-building Clinic: Arihiro Kiyosue, Ryota Tamura

**Canada:** CRIUCPQ: Francois Dube, Marilene Bolduc, Marie-Christine Talbot; University Health Network-Toronto General Hospital: David Cherney, Leslie Cham, Vesta Lai, Josephine Tse; Clinical Research Solutions Inc.: Shivinder Jolly, Tabbatha Duck; Interior Health Kelowna General Hospital: Scott Lyle, Rachel Epp, Camille Galloway, Susan Haskett, Elizabeta Matvienko, Liam Paulsen, Zachary Walbaum; London Health Sciences Centre: Louise Moist, Kerri Gallo, Zabrina Lozon, Tina Ramsey, Brittany Whitmore; St Paul's Hospital: Adeera Levin, Bader Al-Zeer, Paula Macleod, Aoife O'Sullivan, Zainab Sheriff, Sam Tholl; Cambridge Cardiac Care Centre: Amritanshu Pandey, Samantha Armstrong, Bethelihem Gebeyehu, Patrick Toth; LMC Clinical Research Inc. (Thornhill): Ronald Goldenberg, Mahsa Jahangiriesmaili, Shariff Sanguila, Neethi Suresh, Tanvi Talsania; Vancouver General Hospital: Nadia Zalunardo, Bader Al-Zeer, Paula Macleod, Aoife O'Sullivan, Zainab Sheriff; CHU de Quebec-Universite Laval: Mohsen Agharazii, Marie-Pier Roussel, Annie Saillant, France Samson; LMC Clinical Research Inc. Brampton: Harpreet Bajaj, Miken Bhavsar, Parul Dhall, Gagandeep Dhillon, Bhupinder Grewal, Taniya Nimbkar, Radica Richards, Julia Lee; CIUSSS Nord de l'ile de Montreal: Francois Madore, Guylaine Marcotte; LMC Clinical Research Inc. (Bayview): Oren Steen, Mathura Bullen, Shayani Raguwaran, Andre Valleteau; CIUSSS de l'Estrie-CHUS, Hopital Fleurimont: Marie-France Langlois, Christine Brown; Lakeridge Health: Andrew Steele, Melissa Garrity, Taneera Ghate, Holly Robinson, Michael Tolibas; LMC Clinical Research Inc. (Ottawa): Chetna Tailor, Lauren Elliott, Christine McClary-Wright; Fadia El Boreky Medicine Professional: Fadia Boreky, Sameh Fikry, Ayesha Ali, Chintankumar Barot, Wagdy Basily, Bethelihem Gebeyehu, Thisun Saram, Vinay Varad, Karimula Mogal; LMC Clinical Research Inc (Etobicoke): Hasnain Khandwala, Alex Aguilera, Patricia Alvarez, Balwinder Gill, Nazihah Huda, Aamir Navivala, Daniel Pinto, Hitu Sharma; Kidney Care Centre-Fraser Health: Micheli Bevilacqua, Elaine Fung, Geraldine Hernandez, Puneet Mann, Jaskiran Saini, Natasha Curtis; Institut de recherches cliniques de Montreal: Remi Rabasa-Lhoret, Danijela Bovan, Marie Devaux

**Italy:** Policlinico San Martino, Genova: Roberto Pontremoli, Cecilia Barnini, Giovanna Leoncini, Luca Manco, Giulia Nobili; Ospedale Casa Sollievo della Sofferenza, San Giovanni Rotondo: Matteo Piemontese, Filippo Aucella, Rachele Grifa, Francesco Totaro; Policlinico S. Orsola-Malpighi, Bologna: Gaetano La Manna, Irene Capelli, Giuseppe Cianciolo, Sarah Lerario, Fulvia Zappulo; Ospedale S. Giovanni di Dio, Firenze: Alberto Rosati, Filippo Fani, Giuseppe Spatoliatore, Ester Baldini, Francesca Bianchini; AOU Policlinico, Bari: Loreto Gesualdo, Francesco Pesce, Maria Russo, Maria Zippo, Cesira Cafiero, Maria Ficarella, Marica Romano; Ospedale Martini, Torino: Daria Motta, Simona Bianco, Donatella Bilucaglia; Ospedale Maggiore Policlinico, Milano: Piergiorgio Messa, Laura Pavone, Federica Tripodi, Simone Vettoretti, Giuseppe Castellano, Emilietta Brigati; AOU Padova: Paola Fioretto, Gianni Carraro, Filippo Farnia, Anna Postal; Ospedale Sacro Cuore di Gesù, Gallipoli: Alessandro D'Amelio, Antonio Cardone, Giovanni Piccinni, Annalisa Aloisi; ASST Spedali Civili, Brescia: Francesco Scolari, Federico Alberici, Alice Guerini, Chiara Saccà, Chiara Salviani, Roberta Zani; AOU L. Vanvitelli, Napoli: Luca De Nicola, Carlo Garofalo, Maria Elena Liberti, Roberto Minutolo, Luigi Pennino, Lucio Polese; AOU Sant' Andrea, Roma: Paolo Mené, Simona Barberi, Clorinda Falcone; Ospedale Ignazio Veris delli Ponti, Scorrano: Francesco Russo, Maurizio Caroppo; Ospedale di Circolo, Desio: Gennaro Santorelli, Rodolfo Rivera; AOU Policlinico G. Martino, Messina: Domenico Santoro, Alfio Giuffrida, Fortunata Zirino, Roberto Gallo ; Ospedale Civile SS. Antonio e Biagio, Alessandria: Cristina Calvi, Luca Estienne; AOUI, Verona: Giovanni Gambaro, Concetta Gangemi, Vittorio Ortalda, Giuseppina Pessolano; Fondazione Policlinico Universitario Agostino Gemelli, Roma: Giuseppe Grandaliano, Rocco Baccaro, Pietro Ferraro, Roberto Mangiacapra; IRCCS Ospedale San Raffaele, Milano: Marco Melandri, Nadia Foligno, Rita Quartagno, Giuseppe Vezzoli, Elena Brioni, Paola Maiucchi, Tunesi Francesca

# Supplementary methods

*Costing ESKD treatments*

End-stage kidney disease (ESKD) management includes dialysis and transplant. Dialysis was costed based on the number of days on dialysis, the frequency of dialysis and the average cost per session. Kidney transplantation costs comprised of hospital admission for the transplant, and the maintenance costs of immunosuppressive drugs following transplantation (7 mg/day tacrolimus, 2 g/day mycophenolate mofetil and 5 mg/day prednisolone based on expert opinion) until the end of the follow-up/transplant failure. All 2022 costs^1^ were inflated to 2023 values^2^. (Supplementary methods Table 1) The above captures most costs associated with ESKD management, though other hidden costs were not included such as annual follow-up for kidney transplant.

**Supplementary methods Table 1. Summary of frequency and unit costs for different types of ESKD management.**

| **Component of ESKD management** | **Frequency of use** | **Unit cost** |
| --- | --- | --- |
| **Haemodialysis** | 3 sessions / week^3^ | £186.72 / session^1,2^ |
| **Peritoneal dialysis** | 1 session / day^3^ | £88.97 / session^1,2^ |
| **Kidney transplantation** |  |  |
| Hospital admission for the transplant | One-off | £17,101 per admission^1,2^ |
| Maintenance immunosuppressive drugs |  |  |
| Tacrolimus | 7 mg / day | £1.11 / mg^4^ |
| Mycophenolate mofetil | 2 g / day | £0.25 / g^4^ |
| Prednisolone | 5 mg / day | £0.01 / mg^4^ |

*Analyses of quality of life (QoL)*

For a small number of EQ5D assessments with missing response in some domains (n=21), the missing domains were imputed using multinomial logistic regression with age at assessment, sex and all the EQ5D responses at domain level of all participants.

We analysed post-randomization QoL in 2 time periods during follow-up: until 21 months of follow-up and thereafter. The cutting point of 21-month was chosen because EMPA-KIDNEY planned to assess EQ5D at about 18-month follow-up, with 18-month defined in the trial as the follow-up between 15 to 21 months.

A shared parameter model was used to analyse QoL. The approach jointly modelled:

1. The QoL utility at baseline and during follow-up using a linear mixed effects model with random effects for each participant’s QoL in the period of first 21 months follow-up; and
2. The hazard function for time to death using a Weibull survival model in which the scale parameter is assumed to be linearly related to the random effects from the mixed effects model.

The shared parameter model included treatment allocation, baseline QoL, time periods of QoL measurement (≤21 months and >21 months), treatment-by-time periods of QoL measurement interaction, baseline QoL-by-time periods of QoL measurement interaction and the prognostic variables used in the minimization algorithm (age, sex, region, eGFR, uACR and prior diabetes as categorical variables).

The shared parameter model allows for the dependence between QoL utility during follow-up and time to death (i.e. those having larger QoL reduction will generally have a shorter time to death).

*Analyses of time to first occurrence of progression to more advanced CKD stage*

We analysed the following time to first occurrence of CKD progression

- Time to ESKD for all participants
- Time to CKD 5 or ESKD for all participants with eGFR >15 ml/min/1.73 m^2^
- Time to CKD 4, CKD 5 or ESKD for all participants with eGFR >30 ml/min/1.73 m^2^

CKD stages 1-5 were determined by: (1) eGFR measures in the specified range at two consecutive scheduled study follow-up visits at least 30 days apart, with the stage taken from date of the first such measure; or (2) eGFR in the specified range measured at the last scheduled study follow-up visit or the last scheduled visit before death (or withdrawal of consent or loss to follow-up).

Cox proportional hazards regression adjusted for the variables used in the minimization algorithm (age, sex, region, estimated glomerular filtration rate [eGFR], urine albumin-to-creatinine ratio [uACR] and prior diabetes as categorical variables) were used to estimate the hazard ratio associated with allocation to empagliflozin versus placebo (with the Wald chi-square statistic used to both test significance and generate an asymptotic 95% confidence interval). Any ties were handled using Breslow’s method. The effects of allocation to empagliflozin on time to progression to CKD stage 5 or ESKD, and progression to CKD stage 4, 5 or ESKD were assessed restricted to participants at earlier stages at 6-month follow-up (to control for the impact of the acute dip of eGFR on initiation of empagliflozin)^5^.

*Analyses of time to occurrences of hospital admission analyses*

A semi-parametric joint frailty model (one type of shared parameter model) was used to analyse the time to hospital admission (overall and by MedDRA SOC). The approach jointly modelled:

1. The hazard function for recurrent hospitalizations conditional on the patient-specific random frailty; and
2. The hazard function for time to death conditional on the patient-specific random frailty.

We assumed that the patient-specific random frailty follows a gamma distribution with mean 1 and variance θ, where θ is the correlation between the recurrent events. Piecewise constant hazards were assumed for both hazard functions to allow estimation of the likelihood by Gaussian quadrature, with follow-up time split into five equally sized intervals. Hazard ratios for the effect of treatment on the rate of recurrent all-cause hospitalizations and the rate of death were calculated by the model, but only the former was formally interpreted. To improve expected precision around effect size estimates, the model was adjusted for sex, prior diabetes and region (in the same categories used in the minimization process) as well as for age, eGFR and log-transformed uACR, each as continuous variables. Adjustment for baseline predictors as continuous variables where possible was done to improve expected convergence of the models.

*Analyses of rate of healthcare resource outcome*

We analysed the rate of the following healthcare resource outcomes

- Days in and costs of hospital admissions
- Days on and costs of selected concomitant medications
- Days on and costs of kidney replacement therapy

A shared parameter model was used to analyse the rate of healthcare resource outcome. The approach jointly modelled:

1. The rate of total cumulative healthcare resource outcome using a mixed effects Poisson-log model with random effects for each participant’s rate of healthcare resource outcome; and
2. The hazard function for time to death using a Weibull survival model in which the scale parameter is assumed to be linearly related to the random effects from the mixed effects model.

The shared parameter model included treatment allocation and the prognostic variables used in the minimization algorithm (age, sex, region, eGFR, uACR and prior diabetes as categorical variables).

It is hypothesized that patients at higher risk of death are also at higher risk of healthcare resource uses/higher costs before death. However, the risk of healthcare resource use/cost becomes zero following death. By jointly modelling the rate of days/costs of healthcare resource uses and death, we could better capture the overall effect of empagliflozin on the healthcare resource use/costs.

Assessments for the effects of allocation to empagliflozin were additionally performed on each pre-specified type of hospital admissions (by Medical Dictionary for Regulatory Activities (MedDRA) System Organ Class (SOC)), medication categories, and EKSD management.

*Cost-consequence analyses using estimated models*

**Estimation of healthcare resource outcome using the shared parameter models**

We estimated the treatment effect on healthcare resource outcomes over 2 years (median follow-up over active-trial period) by

- Estimating the outcomes for all EMPA-KIDNEY participants under two scenarios: (1) assuming everyone used empagliflozin; (2) assuming no one used empagliflozin;
  - Firstly derive the annual rate of the outcome for the participant from the mixed effect Poisson model
  - Secondly derive the total survival (years) for the participant from the Weibull survival model including the random effect for annual rate of the outcome (see below)
  - Thirdly multiply the two estimates
- Using the estimate from empagliflozin-treated scenario to subtract the estimate from untreated scenario.

Total survival time over a certain timeframe is the surface under the curve from inception to the timeframe of the survival equation for time to death. As the equation that we used for time to death is a Weibull survival model without a closed form to derive the surface under the curve, we derived surface under the curve using the following approximation:

- Firstly we split the target timeframe into cycles (e.g. each cycle with a length of 1-month)
- Secondly we calculated the life year over each cycle assuming patients having the probability of surviving over the cycle with the same probability as those at the beginning of the cycle
  - $h(t_{ij})=\gamma exp \left( \phi+{\eta_{1}u}_{i}+\alpha_{1}X_{i}+\alpha Z_{i} \right)^{\gamma}t_{ij}^{\gamma-1}$
  - $S(t_{ij})=exp(-exp \left( \phi+{\eta_{1}u}_{1i}+\alpha_{1}X_{i}+\alpha Z_{i} \right)^{\gamma}t_{ij}^{\gamma})$
  - $LY_{ij}=S\left( t_{ij} \right)*cycle length$
  - Where
    - $h(t_{ij})$ is the hazard at the entry time of cycle j for participant i
    - $S(t_{ij})$ is the survival probability at the entry of cycle j for participant i
    - $LY_{ij}$ is the life year over cycle j for participant i
    - $t_{ij}$ is the entry time of cycle j for participant i
    - $\gamma$ is the shape parameter of the Weibull survival model
    - $\phi$ is the natural logarithm of the baseline hazard
    - $u_{i}$ is the random effect for the annual rate of the healthcare resource outcome for the participant i
    - $X_{i}$ is the treatment allocation of the participant i
    - $Z_{i}$ is the vector of minimization factors of the participant i
    - $\eta_{1}$, $\alpha_{1}$, and $\alpha$ are the coefficients from the hazard model
- Thirdly, we summed up the calculated life years across cycles

**Estimation of healthcare resource outcomes using the estimated negative binomial models for the healthcare resource outcome and Cox proportional hazards model for the time to death**

For the healthcare resource outcomes where the shared parameter model failed to converge, we estimated the rate of healthcare resource outcomes using negative binomial models and estimated the outcome over 2 years with a separate Cox proportional hazards model for the time to death.

- $H(t_{ij})=h_{0}(t_{j})\exp\left( \alpha_{1}X_{i}+\alpha Z_{i} \right)$
- $S\left( t_{ij} \right)=exp(-H\left( t_{ij} \right))$
- Where
  - ${H(t}_{ij})$ is the cumulative hazard at entry time of cycle j for participant i
  - $S(t_{ij})$ is the survival probability at the entry of cycle j for participant i
  - $h_{0}(t_{j})$ is the baseline hazard at the entry time of cycle j
  - $t_{ij}$ is the entry time of cycle j for participant i
  - $t_{j}$ is the entry time of cycle j
  - $X_{i}$ is the treatment allocation of the participant i
  - $Z_{i}$ is the vector of minimization factors of the participant i
  - $\alpha_{1}$, and $\alpha$ are the coefficients from the hazard model

We estimated the treatment effect on healthcare resource outcomes similar to above, but using the estimated negative binomial model for estimating the rate of the outcome and using Cox proportional hazards model for estimating total life years instead.

**Supplementary methods Figure 1. An illustrative example of steps for total healthcare uses/costs estimation.**

The estimation of total QALYs followed similar steps with the only difference that quality of life was modelled over time in step 1 (instead of modelling rate of healthcare uses/costs). Images by Freepik from Flaticon.com.

**Estimation of quality-adjusted life years (QALYs) using the shared parameter model**

We estimated the treatment effect on quality-adjusted life years (QALYs) over 2 years using the same approach to the survival time calculation mentioned above but adjusting the life year over each cycle by the QoL estimated in the middle of the cycle. For estimating the QoL at each time point, we firstly used the QoLs at baseline, and estimated the mean time of the assessments up until 21 months and that after 21 months from the linear mixed model part of the shared parameter model; then we estimated the QoLs at the time point of interest assuming the QoL declines linearly over time between baseline and mean time of the assessments up until 21 months, and between mean time of the assessments up until 21 months and mean time of the assessments after 21 months. The total QALYs over 2 years were the sum of QALYs across all the cycles over the 2 years.

**Estimation of uncertainty around the treatment effects on the estimates from the cost-consequence analyses**

The uncertainty around the treatment effect was estimated using the bootstrapping approach. We generated bootstrap samples of the study participants stratified by treatment allocation, then estimated the models and performed the calculation as mentioned above. The standard deviance around the bootstrap estimates was the standard error of the estimate. To derive a stable standard error, 1000 bootstraps were performed.

*Amendments made after finalizing the health economic analyses plan*

The following changes were made during the analysis after the health economic analyses plan (HEAP) was finalized:

1. We used negative binomial models instead of shared parameter models to analyze days and costs of ESKD management as the base-case shared parameter models failed to converge. Poisson model was not used due to evidence of over dispersion.
2. We modelled progression to more advanced CKD stages from 6 months in the study instead of from entry to avoid the acute dip in eGFR levels with initiation of empagliflozin.
3. We analyzed QoL by estimating the absolute difference in the QoL over 2 discrete periods (up until 21 months and after 21 months in the study) because we did not find evidence to support the assumptions for a slope in QoL over time.

We estimated active-trial net effects over 2 years instead of over 2.5 years because combining rate analyses with survival analyses for absolute effect estimation performed well over the 2 years mean follow-up period, but its performance worsened in projections beyond observed data. Nevertheless, the estimated net effects over 2.5 years were similar (data not shown).

# Supplementary tables and figures

## Supplementary Figure S1. CONSORT participant flowchart for active-trial and post−trial follow-up (PTFU) periods

**Empagliflozin**

**N=3304**

**Placebo**

**N=3305**

**Attended screening visit**

N=8544

**Randomized**

**N=6609**

**Excluded N=1935**

Before pre-randomization run-in (n=360)

Before randomization (n=1575)

**PTFU analyses N= 2472**

Completed PTFU (n=2427)

Consent withdrawn during PTFU (n=4)

Mortality status unknown after 01Apr24 (n= 41)

**Excluded N=832**

Ineligible for PTFU (n=774)

Others (n=58)

**Active-trial analyses N= 3304**

Completed follow-up (n=3273)

Consent withdrawn (n=22)

Lost to follow-up (n=9)

**Active-trial analyses N= 3305**

Completed follow-up (n=3279)

Consent withdrawn (n=17)

Lost to follow-up (n=9)

**PTFU analyses N= 2419**

Completed PTFU (n=2371)

Consent withdrawn during PTFU (n=3)

Mortality status unknown after 01Apr24 (n= 45)

**Entered PTFU**

N=2472

**Entered PTFU**

N=2419

**Excluded N=886**

Ineligible for PTFU (n=806)

Others (n=80)

Proportions of participants entering PTFU were: Empagliflozin group: 2472/3304 (74.8%) and placebo group 2419/3305 (73.2%).

## Supplementary table S1. Characteristics at randomization of participants entering and not entering post-trial follow-up

|  | **Participants entered PTFU** | | | **Participants not known to have died at the end of active-trial follow-up but did not enter PTFU**  **(n = 1362)** |
| --- | --- | --- | --- | --- |
|  | **Empagliflozin**  **(n=2472)** | **Placebo**  **(N=2419)** | **Overall**  **(n=4891)** |  |
| **Demographic characteristics** |  |  |  |  |
| Age (years) | 63.0 (14.1) | 62.5 (14.2) | 62.8 (14.1) | 65.4 (12.7) |
| Sex: Women | 840 (34.0%) | 824 (34.1%) | 1664 (34.0%) | 433 (31.8%) |
| Race |  |  |  |  |
| White | 1552 (62.8%) | 1503 (62.1%) | 3055 (62.5%) | 554 (40.7%) |
| Black | 91 (3.7%) | 87 (3.6%) | 178 (3.6%) | 66 (4.8%) |
| Asian | 791 (32.0%) | 791 (32.7%) | 1582 (32.3%) | 725 (53.2%) |
| Mixed | 14 (0.6%) | 6 (0.2%) | 20 (0.4%) | 1 (0.1%) |
| Other | 24 (1.0%) | 32 (1.3%) | 56 (1.1%) | 16 (1.2%) |
| Region |  |  |  |  |
| Europe (UK, Germany, Italy) | 1222 (49.4%) | 1160 (48.0%) | 2382 (48.7%) | 111 (8.1%) |
| North American (USA, Canada) | 528 (21.4%) | 528 (21.8%) | 1056 (21.6%) | 539 (39.6%) |
| China, Malaysia | 722 (29.2%) | 731 (30.2%) | 1453 (29.7%) | 124 (9.1%) |
| Japan | 0 (0%) | 0 (0%) | 0 (0%) | 588 (43.2%) |
| **Prior disease** |  |  |  |  |
| Prior diabetes* | 1087 (44.0%) | 1020 (42.2%) | 2107 (43.1%) | 683 (50.1%) |
| History of cardiovascular disease§ | 639 (25.8%) | 641 (26.5%) | 1280 (26.2%) | 311 (22.8%) |
| **Clinical measurements** |  |  |  |  |
| Systolic blood pressure (mmHg) | 136.9 (18.3) | 136.9 (18.3) | 136.9 (18.3) | 134.8 (17.3) |
| Diastolic blood pressure (mmHg) | 78.6 (11.6) | 78.6 (11.8) | 78.6 (11.7) | 77.6 (11.8) |
| Body mass index (kg/m²) | 29.9 (6.6) | 30.0 (6.7) | 29.9 (6.6) | 28.8 (7.0) |
| **Laboratory measurements** |  |  |  |  |
| eGFR (mL/min/1.73m²)† | 36.9 (14.1) | 36.9 (14.1) | 36.9 (14.1) | 40.5 (16.0) |
| <30 | 854 (34.5%) | 857 (35.4%) | 1711 (35.0%) | 378 (27.8%) |
| 30 to 45 | 1128 (45.6%) | 1082 (44.7%) | 2210 (45.2%) | 575 (42.2%) |
| ≥45 | 490 (19.8%) | 480 (19.8%) | 970 (19.8%) | 409 (30.0%) |
| uACR (mg/g)† | 324 (44-1045) | 313 (45-1079) | 317 (44-1063) | 393 (81-1085) |
| <30 | 515 (20.8%) | 515 (21.3%) | 1030 (21.1%) | 218 (16.0%) |
| 30 to 300 | 686 (27.8%) | 677 (28.0%) | 1363 (27.9%) | 380 (27.9%) |
| >300 | 1271 (51.4%) | 1227 (50.7%) | 2498 (51.1%) | 764 (56.1%) |
| NT-proBNP (ng/L) | 161 (72-396) | 152 (68-386) | 156 (70-392) | 132 (54-348) |
| **Concomitant medication use** |  |  |  |  |
| RAS inhibitor | 2142 (86.7%) | 2066 (85.4%) | 4208 (86.0%) | 1141 (83.8%) |
| Any diuretic | 1028 (41.6%) | 1052 (43.5%) | 2080 (42.5%) | 509 (37.4%) |
| Any lipid-lowering medication | 1638 (66.3%) | 1582 (65.4%) | 3220 (65.8%) | 878 (64.5%) |
| **Cause of kidney disease** |  |  |  |  |
| Diabetic kidney disease | 727 (29.4%) | 677 (28.0%) | 1404 (28.7%) | 472 (34.7%) |
| Hypertensive/renovascular disease | 553 (22.4%) | 572 (23.6%) | 1125 (23.0%) | 247 (18.1%) |
| Glomerular disease | 670 (27.1%) | 636 (26.3%) | 1306 (26.7%) | 342 (25.1%) |
| Other/unknown | 522 (21.1%) | 534 (22.1%) | 1056 (21.6%) | 301 (22.1%) |
| **5 year kidney failure risk** | 10% (3%-29%) | 10% (3%-30%) | 10% (3%-29%) | 7% (2%-25%) |
| **Health related quality of life**^¥^ | 0.848 (0.180) | 0.855 (0.168) | 0.851 (0.174) | 0.874 (0.147) |

Figures are n (%), mean (SD) or median (Q1-Q3). NT-proBNP, N-terminal pro B-type natriuretic peptide. eGFR, estimated glomerular filtration rate. uACR, urine albumin-to-creatinine ratio. RAS, renin-angiotensin system. *Participant-reported history of diabetes of any type, use of glucose-lowering medication or baseline HbA1c ≥48 mmol/mol at randomization. §Self-reported history of myocardial infarction, heart failure, stroke, transient ischemic attack, or peripheral arterial disease. †Uses central measurement taken at the randomization visit, or more recent local laboratory result before randomization. ^¥^EQ-5D utility.

## Supplementary table S2. Effect of allocation to empagliflozin on **time to first ESKD**, **time to CKD5 or ESKD and time to CKD4, CKD5 or ESKD**

| Time to progression of CKD stage ^a^ | Empagliflozin | | Placebo | | Effect of allocation to empagliflozin,  Hazard ratio (95%CI) |
| --- | --- | --- | --- | --- | --- |
|  | **Total** | **N event (%)** | **Total** | **N event (%)** |  |
| Based on ACTIVE-TRIAL data |  |  |  |  |  |
| To ESKD | 3304 | 108 (3.3%) | 3305 | 158 (4.8%) | 0.67 (0.52-0.85) |
| CKD 1-4 to CKD 5 or ESKD ^b^ | 3238 | 248 (7.7%) | 3230 | 320 (9.9%) | 0.71 (0.60-0.84) |
| CKD 1-3 to CKD 4-5 or ESKD ^b^ | 1841 | 372 (20.2%) | 1983 | 482 (24.3%) | 0.81 (0.71-0.93) |
| Based on ACTIVE- and POST-TRIAL data combined | | | | | |
| To ESKD | 3304 | 296 (9.0%) | 3305 | 372 (11.3%) | 0.74 (0.64-0.87) |

Hazard ratio estimates are derived from Cox proportional hazards models adjusted for allocation to empagliflozin and baseline variables specified in the minimization algorithm (age, sex, previous diabetes, eGFR, uACR, and region). ^a^ CKD stage includes CKD 1-5 defined based on eGFR (ml/min/1.73m^2^) (1: ≥90; 2: 60-90; 3: 30-60; 4: 15-30; 5: <15) and ESKD defined by the initiation of maintenance dialysis or receipt of a kidney transplant. ^b^ To assess the long-term effects of empagliflozin, the first 6 months including the initial acute dip in kidney function were excluded. CKD, chronic kidney disease; eGFR, estimated glomerular filtration rate; ESKD, end stage kidney disease; uACR, urine albumin-to-creatinine ratio

## Supplementary table S3. Effects of allocation to empagliflozin on **QoL and on hospital admissions, days of and costs of healthcare use**

|  | **Raw mean (SD)** | | **Estimated effects (95%CI)** |
| --- | --- | --- | --- |
|  | **Empagliflozin**  **N = 3304** | **Placebo**  **N = 3305** |  |
| **Based on ACTIVE-TRIAL data** |  |  |  |
| *QoL* | *N_1_* | *N_2_* | *Mean difference (95%CI)* |
| Baseline (N_1_=3303; N_2_=3305) | 0.849 (0.177) | 0.853 (0.170) | 0.000 (-0.005, 0.005) ^a^ |
| ≤21 months (N_1_=3089;N_2_=3074) | 0.850 (0.193) | 0.848 (0.194) | 0.007 (-0.002, 0.015) ^a^ |
| >21 months (N_1_=1857;N_2_=1813) | 0.832 (0.212) | 0.831 (0.210) | 0.005 (-0.003, 0.013) ^a^ |
| *Number of event / person-year* |  |  | *Hazard ratio (95%CI)* |
| Hospital admission | 0.248 | 0.293 ^b^ | 0.86 (0.78-0.95) |
| *Days of healthcare use / year* |  |  | *Rate ratio (95%CI)* |
| Hospital admission | 2.58 (8.90) | 2.91 (10.17) | 0.82 (0.64-1.03) |
| Individual concomitant medications ^c^ | 1773 (864) | 1821 (888) | 0.98 (0.95-1.00) |
| ESKD management | 4.01 (27.28) | 5.18 (28.63) | 0.77 (0.41-1.47) ^d^ |
| *Costs of healthcare use / year* |  |  | *Rate ratio (95%CI)* |
| Hospital admission | £582 (1592) | £699 (2069) | 0.81 (0.56-1.19) |
| Individual concomitant medications ^c^ | £649 (909) | £699 (944) | 0.90 (0.85-0.96) |
| ESKD management | £313 (2142) | £422 (2341) | 0.74 (0.33-1.70) ^d^ |
| **Based on POST-TRIAL data** |  |  |  |
| Days of ESKD management | 22 (87) | 27 (97) | 0.80 (0.51-1.24) ^d^ |
| Costs of ESKD management | £1646 (6671) | £2134 (7703) | 0.78 (0.45-1.36) ^d^ |

Unless otherwise specified, all analyses were based on shared parameter model adjusted for allocation to empagliflozin and baseline variables specified in the minimization algorithm (age, sex, previous diabetes, eGFR, uACR, and region). ^a^ Estimated from shared parameter model adjusted for allocation to empagliflozin, QoL at baseline, QoL at baseline interacted with period of assessment, period of assessment, period of assessment interacted with treatment allocation, and baseline variables specified in the minimization algorithm; ^b^ different from the value (0.292) shown in table 2 in the main paper of the EMPA-KIDNEY trial^6^ because we identified and corrected an error of the admission date of one hospital episode in this analysis; ^c^ selected concomitant medications included: antihypertensive treatments, antiplatelets, anticoagulants, diabetes medications, lipid lowering medications, drug for anemia, uric acid lowering medications, and phosphate binders; Each day on individual concomitant medication contributes a ‘medication day’ in the total concomitant medication days; ^d^ based on negative binomial model without adjustments. eGFR, estimated glomerular filtration rate; ESKD, end stage kidney disease; QoL, quality of life; uACR, urine albumin-to-creatinine ratio.

## Supplementary table S4. Effects of allocation to empagliflozin on **days on and costs of individual concomitant medication of interest**, overall and by each category of interest (Limited to active-trial data)

|  | Days on individual concomitant medication per year, Mean (SD) | | Effect of allocation to empagliflozin on days on individual concomitant medication,  Rate ratio (95%CI) | Costs of concomitant medication per year,  Mean (£, SD) | | Effect of allocation to empagliflozin on costs of concomitant medication,  Rate ratio (95%CI) |
| --- | --- | --- | --- | --- | --- | --- |
|  | **Empagliflozin**  **(N = 3304)** | **Placebo**  **(N = 3304)** |  | **Empagliflozin**  **(N = 3304)** | **Placebo**  **(N = 3304)** |  |
| All categories of interest | **1773 (864)** | **1821 (888)** | **0.98 (0.95-1.00)** | **£649 (909)** | **£699 (944)** | **0.90 (0.85-0.96)** |
| By categories of interest |  |  |  |  |  |  |
| Any antihypertensive | 923 (482) | 956 (494) | 0.96 (0.92-1.00) | £171 (284) | £195 (301) | 0.89 (0.83-0.94) |
| *Renin-angiotensin-system inhibitor* | 302 (132) | 303 (133) | 1.00 (0.90-1.12) | £37 (73) | £39 (77) | 0.98 (0.91-1.05) |
| *Beta blocker* | 156 (177) | 155 (178) | 1.15 (0.79-1.68) | £16 (33) | £17 (36) | 1.07 (0.85-1.35) |
| *Calcium channel blockers* | 199 (191) | 208 (192) | 0.74 (0.56-0.97) | £13 (19) | £14 (20) | 0.85 (0.73-0.99) |
| *Mineralocorticoid receptor antagonists* | 24 (86) | 27 (92) | 0.87 (0.48-1.59) | £2 (7) | £2 (8) | 0.87 (0.52-1.45) |
| *Other diuretics* | 154 (199) | 169 (202) | 0.50 (0.35-0.71) | £92 (258) | £112 (279) | 0.55 (0.41-0.72) |
| *Other antihypertensive* | 89 (173) | 94 (179) | 0.90 (0.63-1.30) | £10 (34) | £11 (38) | 0.88 (0.64-1.20) |
| Anticoagulant/Antiplatelet | 178 (212) | 184 (215) | 0.86 (0.63-1.19) | £94 (291) | £95 (279) | 0.87 (0.69-1.10) |
| *Anticoagulant* | 44 (117) | 46 (117) | 0.92 (0.57-1.49) | £78 (254) | £80 (250) | 0.92 (0.56-1.49) |
| *Antiplatelet* | 134 (192) | 138 (194) | 0.87 (0.60-1.26) | £16 (149) | £15 (132) | 0.91 (0.71-1.16) |
| Anti-diabetics | 251 (345) | 250 (343) | 0.85 (0.73-1.01) | £260 (623) | £268 (644) | 0.85 (0.72-1.01) |
| Lipid lowering | 277 (209) | 277 (210) | 0.99 (0.82-1.18) | £39 (307) | £35 (283) | 0.99 (0.90-1.10) |
| Drug for anaemia | 9 (50) | 12 (57) | 0.63 (0.27-1.48)^a^ | £57 (331) | £77 (383) | 0.66 (0.27-1.59) |
| Uric acid lowering | 133 (171) | 138 (172) | 0.85 (0.61-1.18) | £24 (115) | £26 (124) | 0.79 (0.60-1.03) |
| Phosphate binders | 3 (31) | 3 (29) | 1.54 (0.46-5.18)^b^ | £4 (49) | £3 (31) | 1.25 (0.39-4.01)^b^ |

Each day on individual concomitant medication contributes a ‘medication day’ in the total concomitant medication days; Unless further specified, rate ratio estimates are derived from shared parameter models adjusted for allocation to empagliflozin and baseline variables specified in the minimization algorithm (age, sex, previous diabetes, eGFR, uACR, and region); ^a^ Rate ratio estimated based on shared parameter models adjusting for only treatment allocation as the fully adjusted model did not converge; ^b^ Rate ratio estimated based on negative binomial models adjusting for treatment allocation and baseline variables specified in the minimization algorithm as the shared parameter model did not converge. eGFR, estimated glomerular filtration rate; uACR, urine albumin-to-creatinine ratio.

## Supplementary table S5. Effect of 2 years treatment with empagliflozin on **days of and costs of ESKD management**, by year of analysis

|  | Number of participants | | Follow-up years,  Mean (SD) | EKSD days per year, Mean (SD) | | Rate ratio  (95%CI) | EKSD costs per year, Mean (SD) | | Rate ratio  (95%CI) |
| --- | --- | --- | --- | --- | --- | --- | --- | --- | --- |
|  | **Empagliflozin** | **Placebo** |  | **Empagliflozin** | **Placebo** |  | **Empagliflozin** | **Placebo** |  |
| Based on ACTIVE-TRIAL data | | | | | | | | | |
| Year 1 | 3304 | 3305 | 0.99 (0.06) | 1 (15) | 1 (13) | 1.19 (0.36-3.97) | £101 (1246) | £90 (1132) | 1.13 (0.23-5.56) |
| Year 2 | 3239 | 3235 | 0.76 (0.30) | 6 (43) | 8 (46) | 0.79 (0.38-1.63) | £465 (3385) | £629 (3854) | 0.74 (0.28-1.92) |
| Year 3+ | 1653 | 1650 | 0.46 (0.26) | 11 (61) | 19 (79) | 0.60 (0.28-1.31) | £879 (5071) | £1437 (6277) | 0.61 (0.22-1.70) |
| Based on POST-TRIAL data | | | | | | | | | |
| Year 1 | 2472^a^ | 2419^a^ | 0.99 (0.09) | 18 (73) | 23 (83) | 0.77 (0.45-1.31) | £1410 (6034) | £1813 (6667) | 0.78 (0.39-1.54) |
| Year 2+ | 2385 | 2329 | 0.99 (0.15) | 31 (97) | 39 (108) | 0.81 (0.52-1.25) | £2250 (7488) | £2891 (8448) | 0.78 (0.45-1.36) |

Rate ratio estimates are derived from negative binomial model adjusting for only treatment allocation as the shared parameter models and fully adjusted negative binomial models did not converge. ^a^ 12 participants, 6 in each arm, entering post-trial follow-up but censored at the first day of post-trial follow-up were excluded from the rate analyses. ESKD, end stage kidney disease.

## Supplementary figure S2. Incremental costs and incremental QALYs over 2 and 4 years with allocation to 2 years empagliflozin treatment in EMPA-KIDNEY (1000 bootstrapping samples)


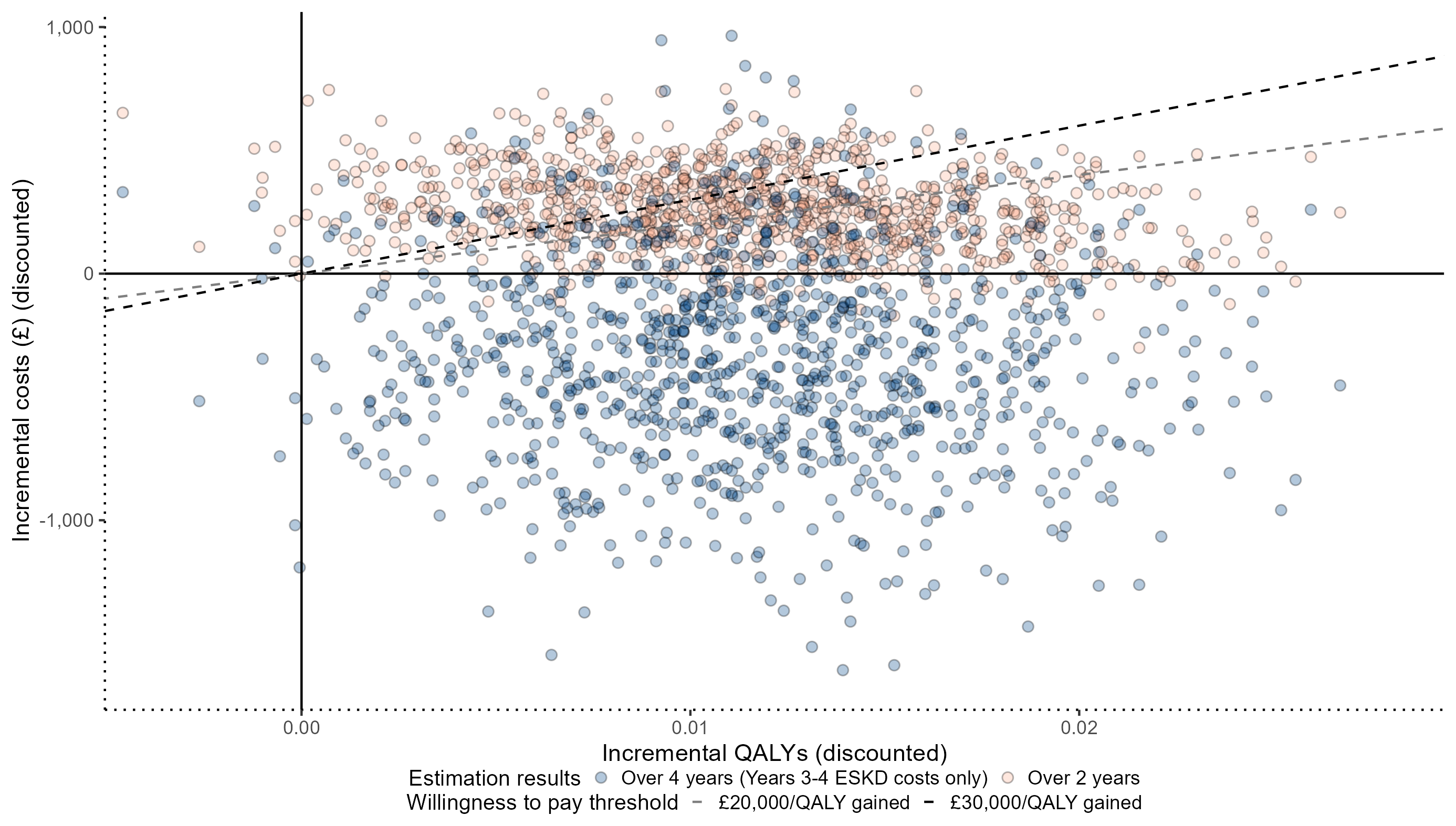


At willingness to pay thresholds of £20,000 and £30,000 per QALY gained, the probabilities of empagliflozin being cost-effective over 2 years were 43% and 61% and over 4 years were 91% and 94%, respectively (proportion of bootstrapping samples falling below the line for the given willingness to pay threshold). ESKD = end-stage kidney disease (i.e. maintenance dialysis or kidney transplantation); QALY = quality-adjusted life year; per QALY gained, respectively.

## Supplementary table S6. Effect of allocation to empagliflozin on **QoL**, overall and by subgroups at baseline (Limited to active-trial data)

| Subgroup at baseline | | # Patients | | Effect on QoL over >0, ≤ 21 months | | Effect on QoL > 21 months | |
| --- | --- | --- | --- | --- | --- | --- | --- |
| Name | **Level** | **Empagliflozin** | **Placebo** | **Mean (95%CI)** | **P trend / Heterogeneity** | **Mean (95%CI)** | **P trend / Heterogeneity** |
| All | **-** | **3304** | **3305** | **0.007 (-0.002 to 0.015)** | **-** | **0.005 (-0.003 to 0.013)** | **-** |
| Age (year) | <60 | 1136 | 1116 | 0.003 (-0.011 to 0.018) | 0.490 ^a^ | 0.009 (-0.005 to 0.023) | 0.402 ^a^ |
|  | 60-70 | 853 | 867 | 0.012 (-0.005 to 0.029) |  | 0.000 (-0.015 to 0.015) |  |
|  | ≥70 | 1315 | 1322 | 0.005 (-0.009 to 0.019) |  | 0.004 (-0.009 to 0.017) |  |
| Sex | Male | 2207 | 2210 | 0.012 (0.001 to 0.022) | 0.105 | 0.002 (-0.008 to 0.012) | 0.329 |
|  | Female | 1097 | 1095 | -0.004 (-0.019 to 0.012) |  | 0.011 (-0.003 to 0.025) |  |
| Region | Europe | 1344 | 1304 | 0.002 (-0.012 to 0.016) | 0.629 | -0.003 (-0.015 to 0.009) | 0.180 |
|  | North America | 251 | 237 | -0.001 (-0.032 to 0.031) |  | 0.004 (-0.030 to 0.038) |  |
|  | Japan | 304 | 308 | 0.020 (-0.008 to 0.049) |  | 0.026 (0.003 to 0.049) |  |
|  | China and Malaysia | 1405 | 1456 | 0.010 (-0.003 to 0.023) |  | 0.008 (-0.004 to 0.021) |  |
| eGFR (mL/min/1.73m2) | <30 | 1131 | 1151 | 0.008 (-0.007 to 0.023) | 0.493 ^a^ | 0.012 (-0.002 to 0.025) | 0.497 ^a^ |
|  | 30-45 | 1467 | 1461 | 0.006 (-0.007 to 0.019) |  | -0.006 (-0.018 to 0.006) |  |
|  | ≥45 | 706 | 693 | 0.006 (-0.012 to 0.025) |  | 0.017 (0.000 to 0.035) |  |
| uACR (mg/g) | <30 | 665 | 663 | -0.001 (-0.020 to 0.019) | 0.217 ^a^ | -0.008 (-0.026 to 0.009) | 0.003 ^a^ |
|  | ≥30<300 | 927 | 937 | 0.008 (-0.009 to 0.024) |  | -0.002 (-0.017 to 0.013) |  |
|  | ≥300<1000 | 844 | 820 | 0.004 (-0.013 to 0.022) |  | 0.024 (0.007 to 0.040) |  |
|  | ≥1000 | 868 | 885 | 0.013 (-0.004 to 0.030) |  | 0.005 (-0.011 to 0.021) |  |
| Diabetes status | Absence | 1779 | 1790 | 0.006 (-0.006 to 0.018) | 0.828 | 0.007 (-0.004 to 0.018) | 0.582 |
|  | Presence | 1525 | 1515 | 0.008 (-0.005 to 0.021) |  | 0.003 (-0.009 to 0.014) |  |
| Primary kidney diagnosis | Diabetic kidney disease | 1032 | 1025 | 0.009 (-0.006 to 0.025) | 0.027 | 0.005 (-0.010 to 0.019) | 0.018 |
|  | Hypertension/reno-vascular | 706 | 739 | -0.007 (-0.026 to 0.011) |  | -0.018 (-0.035 to -0.001) |  |
|  | Glomerular diseases | 853 | 816 | -0.004 (-0.021 to 0.013) |  | 0.010 (-0.006 to 0.027) |  |
|  | Other or unknown | 713 | 725 | 0.029 (0.010 to 0.047) |  | 0.020 (0.003 to 0.037) |  |
| 5-year kidney failure risk | <5% | 1186 | 1191 | 0.004 (-0.010 to 0.019) | 0.327 ^a^ | 0.003 (-0.011 to 0.016) | 0.365^a^ |
|  | ≥5%<20% | 1039 | 1027 | 0.004 (-0.011 to 0.020) |  | 0.003 (-0.011 to 0.017) |  |
|  | ≥20% | 1079 | 1087 | 0.012 (-0.004 to 0.027) |  | 0.009 (-0.005 to 0.023) |  |

Mean estimates are derived from shared parameter models adjusted for allocation to empagliflozin, QoL at baseline, period of assessment, period of assessment interacted with QoL at baseline, period of assessment interacted with treatment allocation, subgroup of interest, subgroup of interest interacted with allocation to empagliflozin, subgroup of interest interacted with period of assessment, subgroup of interest interacted with allocation to empagliflozin and period of assessment, and the other baseline variables specified in the minimization algorithm (age, sex, previous diabetes, eGFR, uACR, and region, excluding the one being the subgroup of interest). ^a^ test for trend; eGFR, estimated glomerular filtration rate. QoL, quality of life. uACR, urine albumin-to-creatinine ratio.

## Supplementary table S7. Effect of allocation to empagliflozin on **hospital costs**, overall and by subgroups at baseline (Limited to active-trial data)

| Subgroup at baseline | | # Patients | | Hospital costs per year (UK£), Mean (SD) | | Effect of allocation to empagliflozin on hospital costs | |
| --- | --- | --- | --- | --- | --- | --- | --- |
| Name | **Level** | **Empagliflozin** | **Placebo** | **Empagliflozin** | **Placebo** | **Hazard ratio (95%CI)** | **P trend / Heterogeneity** |
| All | **-** | **3304** | **3305** | **£582 (1592)** | **£699 (2069)** | **0.81 (0.56-1.19)** | **-** |
| Age (year) | <60 | 1136 | 1116 | £344 (1134) | £352 (1275) | 1.09 (0.55-2.18) | 0.051 ^a^ |
|  | 60-70 | 853 | 867 | £628 (1755) | £645 (1750) | 0.92 (0.45-1.90) |  |
|  | ≥70 | 1315 | 1322 | £758 (1783) | £1027 (2668) | 0.62 (0.35-1.09) |  |
| Sex | Male | 2207 | 2210 | £637 (1743) | £702 (1914) | 0.90 (0.57-1.42) | 0.460 |
|  | Female | 1097 | 1095 | £471 (1224) | £693 (2352) | 0.66 (0.34-1.29) |  |
| Region | Europe | 1344 | 1304 | £704 (1719) | £887 (2602) | 0.83 (0.47-1.45) ^b^ | 0.986 ^b^ |
|  | North America | 251 | 237 | £481 (1839) | £601 (1948) | 0.80 (0.16-3.91) ^b^ |  |
|  | Japan | 304 | 308 | £431 (1194) | £639 (1784) | 1.02 (0.31-3.35) ^b^ |  |
|  | China and Malaysia | 1405 | 1456 | £516 (1482) | £558 (1522) | 0.80 (0.45-1.41) ^b^ |  |
| eGFR (mL/min/1.73m2) | <30 | 1131 | 1151 | £737 (1731) | £885 (2653) | 0.84 (0.45-1.56) | 0.489 ^a^ |
|  | 30-45 | 1467 | 1461 | £547 (1648) | £661 (1756) | 0.75 (0.42-1.32) |  |
|  | ≥45 | 706 | 693 | £407 (1160) | £470 (1460) | 0.97 (0.41-2.30) |  |
| uACR (mg/g) | <30 | 665 | 663 | £576 (1503) | £824 (2198) | 0.68 (0.30-1.56) | 0.454 ^a^ |
|  | ≥30<300 | 927 | 937 | £611 (1736) | £755 (2569) | 0.78 (0.38-1.61) |  |
|  | ≥300<1000 | 844 | 820 | £529 (1477) | £489 (1434) | 1.03 (0.48-2.23) |  |
|  | ≥1000 | 868 | 885 | £608 (1604) | £739 (1853) | 0.78 (0.38-1.59) |  |
| Diabetes status | Absence | 1779 | 1790 | £413 (1226) | £480 (1630) | 0.90 (0.53-1.54) | 0.596 |
|  | Presence | 1525 | 1515 | £779 (1914) | £957 (2466) | 0.73 (0.43-1.25) |  |
| Primary kidney diagnosis | Diabetic kidney disease | 1032 | 1025 | £743 (1873) | £897 (2005) | 0.69 (0.36-1.32) | 0.869 |
|  | Hypertension/reno-vascular | 706 | 739 | £593 (1530) | £750 (2782) | 0.90 (0.40-1.99) |  |
|  | Glomerular diseases | 853 | 816 | £312 (995) | £374 (1220) | 0.74 (0.33-1.66) |  |
|  | Other or unknown | 713 | 725 | £662 (1743) | £733 (2026) | 1.04 (0.47-2.30) |  |
| 5-year kidney failure risk | <5% | 1186 | 1191 | £501 (1445) | £669 (1920) | 0.85 (0.46-1.58) ^b^ | 0.500 ^a, b^ |
|  | ≥5%<20% | 1039 | 1027 | £603 (1618) | £732 (2491) | 0.80 (0.41-1.54) ^b^ |  |
|  | ≥20% | 1079 | 1087 | £650 (1713) | £700 (1768) | 0.85 (0.45-1.60) ^b^ |  |

Rate ratios are estimated from shared parameter models adjusted for allocation to empagliflozin, subgroup of interest, interaction between allocation to empagliflozin and subgroup of interest, and the other baseline variables specified in the minimization algorithm (age, sex, previous diabetes, eGFR, uACR, and region, excluding the one being the subgroup of interest). ^a^ test for trend; ^b^ estimated from shared parameter models adjusting only for treatment allocation, subgroup and their interaction as the fully adjusted models did not converge; eGFR, estimated glomerular filtration rate. uACR, urine albumin-to-creatinine ratio.

## Supplementary table S8. Effect of allocation to empagliflozin on **costs of concomitant medications of interest**, overall and by subgroups at baseline (Limited to active-trial data)

| Subgroup at baseline | | # Patients | | Concomitant medication costs per year (UK£), Mean (SD) | | Effect of allocation to empagliflozin on concomitant medication costs | | |
| --- | --- | --- | --- | --- | --- | --- | --- | --- |
| Name | **Level** | **Empagliflozin** | **Placebo** | **Empagliflozin** | **Placebo** | **Rate ratio (95%CI)** | **P trend / Heterogeneity** |  |
| All | **-** | **3304** | **3305** | **£649 (909)** | **£699 (944)** | **0.90 (0.85-0.96)** | **-** |  |
| Age (year) | <60 | 1136 | 1116 | £383 (694) | £421 (674) | 0.85 (0.77-0.95) | <0.001 ^a^ |  |
|  | 60-70 | 853 | 867 | £715 (954) | £781 (1025) | 0.87 (0.78-0.98) |  |  |
|  | ≥70 | 1315 | 1322 | £836 (987) | £879 (1027) | 0.97 (0.88-1.06) |  |  |
| Sex | Male | 2207 | 2210 | £683 (946) | £710 (946) | 0.93 (0.87-1.01) | 0.081 |  |
|  | Female | 1097 | 1095 | £580 (826) | £678 (940) | 0.84 (0.75-0.93) |  |  |
| Region | Europe | 1344 | 1304 | £666 (889) | £702 (914) | 0.91 (0.83-1.00) | 0.766 |  |
|  | North America | 251 | 237 | £1071 (1409) | £1099 (1376) | 1.00 (0.80-1.24) |  |  |
|  | Japan | 304 | 308 | £539 (644) | £712 (936) | 0.89 (0.73-1.08) |  |  |
|  | China and Malaysia | 1405 | 1456 | £581 (841) | £628 (868) | 0.88 (0.80-0.96) |  |  |
| eGFR (mL/min/1.73m2) | <30 | 1131 | 1151 | £702 (905) | £780 (986) | 0.90 (0.82-1.00) | 0.109 ^a^ |  |
|  | 30-45 | 1467 | 1461 | £699 (996) | £714 (970) | 0.95 (0.87-1.04) |  |  |
|  | ≥45 | 706 | 693 | £460 (674) | £534 (786) | 0.81 (0.71-0.92) |  |  |
| uACR (mg/g) | <30 | 665 | 663 | £841 (1118) | £833 (1067) | 1.00 (0.87-1.14) | 0.012 ^a^ |  |
|  | ≥30<300 | 927 | 937 | £697 (905) | £744 (941) | 0.90 (0.81-1.01) |  |  |
|  | ≥300<1000 | 844 | 820 | £502 (772) | £577 (864) | 0.86 (0.76-0.96) |  |  |
|  | ≥1000 | 868 | 885 | £593 (827) | £664 (906) | 0.87 (0.78-0.98) |  |  |
| Diabetes status | Absence | 1779 | 1790 | £332 (571) | £361 (580) | 0.89 (0.82-0.96) | 0.614 |  |
|  | Presence | 1525 | 1515 | £1019 (1076) | £1098 (1120) | 0.91 (0.84-1.00) |  |  |
| Primary kidney diagnosis | Diabetic kidney disease | 1032 | 1025 | £1060 (1123) | £1128 (1134) | 0.93 (0.84-1.03) | 0.034 |  |
|  | Hypertension/reno-vascular | 706 | 739 | £581 (794) | £610 (846) | 0.98 (0.86-1.11) |  |  |
|  | Glomerular diseases | 853 | 816 | £295 (525) | £384 (646) | 0.78 (0.69-0.87) |  |  |
|  | Other or unknown | 713 | 725 | £545 (803) | £539 (803) | 0.95 (0.83-1.07) |  |  |
| 5-year kidney failure risk | <5% | 1186 | 1191 | £642 (929) | £660 (891) | 0.88 (0.80-0.97) | 0.425 ^a^ |  |
|  | ≥5%<20% | 1039 | 1027 | £680 (935) | £772 (1066) | 0.92 (0.83-1.02) |  |  |
|  | ≥20% | 1079 | 1087 | £627 (862) | £673 (873) | 0.91 (0.82-1.01) |  |  |

Rate ratios are estimated from shared parameter models adjusted for allocation to empagliflozin, subgroup of interest, interaction between allocation to empagliflozin and subgroup of interest, and the other baseline variables specified in the minimization algorithm (age, sex, previous diabetes, eGFR, uACR, and region, excluding the one being the subgroup of interest). ^a^ test for trend. eGFR, estimated glomerular filtration rate. uACR, urine albumin-to-creatinine ratio.

## Supplementary table S9. Effect of allocation to empagliflozin on **costs of ESKD management**, overall and by subgroups at baseline (Limited to active-trial data)

| Subgroup at baseline | | # Patients | | EKSD costs per year (UK£), Mean (SD) | | Effect of allocation to empagliflozin on ESKD costs | |
| --- | --- | --- | --- | --- | --- | --- | --- |
| Name | **Level** | **Empagliflozin** | **Placebo** | **Empagliflozin** | **Placebo** | **Rate ratio (95%CI)** | **P trend / Heterogeneity** |
| All | **-** | **3304** | **3305** | **£313 (2142)** | **£422 (2341)** | **0.74 (0.33-1.70)** | **-** |
| Age (year) | <60 | 1136 | 1116 | £469 (2556) | £612 (2779) | 0.77 (0.19-3.13) | 0.350 ^a^ |
|  | 60-70 | 853 | 867 | £388 (2468) | £354 (2128) | 1.09 (0.22-5.48) |  |
|  | ≥70 | 1315 | 1322 | £131 (1371) | £306 (2043) | 0.43 (0.12-1.57) |  |
| Sex | Male | 2207 | 2210 | £363 (2324) | £480 (2490) | 0.76 (0.28-2.08) | 0.925 |
|  | Female | 1097 | 1095 | £213 (1713) | £306 (2003) | 0.70 (0.17-2.92) |  |
| Region | Europe | 1344 | 1304 | £326 (2216) | £395 (2276) | 0.83 (0.22-3.04) | 0.988 |
|  | North America | 251 | 237 | £75 (704) | £157 (1680) | 0.48 (0.02-9.96) |  |
|  | Japan | 304 | 308 | £257 (1961) | £427 (2413) | 0.60 (0.04-8.94) |  |
|  | China and Malaysia | 1405 | 1456 | £356 (2272) | £489 (2471) | 0.73 (0.21-2.55) |  |
| eGFR (mL/min/1.73m2) | <30 | 1131 | 1151 | £775 (3369) | £973 (3558) | 0.80 (0.20-3.11) | 0.420 ^a^ |
|  | 30-45 | 1467 | 1461 | £92 (1080) | £155 (1306) | 0.60 (0.18-1.99) |  |
|  | ≥45 | 706 | 693 | £34 (592) | £69 (869) | 0.49 (0.09-2.78) |  |
| uACR (mg/g) | <30 | 665 | 663 | £53 (711) | £98 (998) | 0.53 (0.09-3.22) | 0.474 ^a^ |
|  | ≥30<300 | 927 | 937 | £109 (1297) | £137 (1463) | 0.80 (0.17-3.62) |  |
|  | ≥300<1000 | 844 | 820 | £184 (1671) | £188 (1339) | 0.98 (0.20-4.87) |  |
|  | ≥1000 | 868 | 885 | £857 (3488) | £1183 (3876) | 0.72 (0.15-3.46) |  |
| Diabetes status | Absence | 1779 | 1790 | £317 (2145) | £385 (2281) | 0.82 (0.27-2.53) | 0.799 |
|  | Presence | 1525 | 1515 | £309 (2138) | £465 (2411) | 0.66 (0.20-2.24) |  |
| Primary kidney diagnosis | Diabetic kidney disease | 1032 | 1025 | £368 (2332) | £491 (2459) | 0.75 (0.17-3.28) | 1.000 |
|  | Hypertension/reno-vascular | 706 | 739 | £211 (1695) | £288 (1934) | 0.73 (0.13-4.26) |  |
|  | Glomerular diseases | 853 | 816 | £423 (2489) | £568 (2905) | 0.74 (0.14-3.83) |  |
|  | Other or unknown | 713 | 725 | £206 (1760) | £298 (1756) | 0.69 (0.12-4.03) |  |
| 5-year kidney failure risk | <5% | 1186 | 1191 | £22 (464) | £52 (788) | 0.42 (0.11-1.56) | 0.356 ^a^ |
|  | ≥5%<20% | 1039 | 1027 | 129 (1341) | 147 (1229) | 0.87 (0.21-3.61) |  |
|  | ≥20% | 1079 | 1087 | 812 (3422) | 1087 (3729) | 0.74 (0.19-2.97) |  |

Rate ratios are estimated from negative binomial model adjusting for only treatment allocation, subgroup and their interaction as the shared parameter models and fully adjusted negative binomial models did not converge; ^a^ test for trend; ESKD, end stage kidney disease.

## Supplementary table S10. Effect of allocation to empagliflozin on **costs of ESKD management**, overall and by subgroups at baseline (Limited to post-trial data)

| Subgroup at baseline | | # Patients | | EKSD costs per year (UK£), Mean (SD) | | Effect of allocation to empagliflozin on ESKD costs | |
| --- | --- | --- | --- | --- | --- | --- | --- |
| Name | **Level** | **Empagliflozin** | **Placebo** | **Empagliflozin** | **Placebo** | **Rate ratio (95%CI)** | **P trend / Heterogeneity** |
| All | **-** | **2472** | **2419** | **£1844 (6301)** | **£2362 (7163)** | **0.78 (0.45-1.36)** | **-** |
| Age (year) | <60 | 913 | 914 | £2897 (7638) | £3527 (8581) | 0.82 (0.33-2.02) | 0.493 ^a^ |
|  | 60-70 | 636 | 613 | £1606 (5967) | £2183 (6784) | 0.74 (0.25-2.19) |  |
|  | ≥70 | 923 | 892 | £969 (4721) | £1289 (5437) | 0.75 (0.30-1.86) |  |
| Sex | Male | 1632 | 1595 | £2093 (6678) | £2570 (7426) | 0.81 (0.41-1.61) | 0.792 |
|  | Female | 840 | 824 | £1363 (5467) | £1959 (6608) | 0.70 (0.27-1.80) |  |
| Region | Europe | 1222 | 1160 | £1754 (6109) | £1909 (6423) | 0.92 (0.42-2.03) | 0.891 |
|  | North America | 156 | 145 | £515 (3475) | £728 (4280) | 0.71 (0.08-6.60) |  |
|  | Japan^b^ | 0 | 0 | - | - | - |  |
|  | China and Malaysia | 1094 | 1114 | £2133 (6780) | £3046 (8069) | 0.70 (0.31-1.59) |  |
| eGFR (mL/min/1.73m2) | <30 | 854 | 857 | £3919 (8884) | £4737 (9656) | 0.83 (0.33-2.08) | 0.496 ^a^ |
|  | 30-45 | 1128 | 1082 | £860 (4197) | £1291 (5354) | 0.67 (0.30-1.50) |  |
|  | ≥45 | 490 | 480 | £500 (3300) | £518 (3352) | 0.96 (0.28-3.25) |  |
| uACR (mg/g) | <30 | 515 | 515 | £352 (3180) | £225 (2289) | 1.57 (0.49-5.04) | 0.363 ^a^ |
|  | ≥30<300 | 686 | 677 | £436 (3019) | £732 (4236) | 0.60 (0.22-1.65) |  |
|  | ≥300<1000 | 633 | 575 | £1326 (5125) | £2027 (6407) | 0.65 (0.22-1.93) |  |
|  | ≥1000 | 638 | 652 | £5073 (9760) | £6032 (10561) | 0.84 (0.30-2.39) |  |
| Diabetes status | Absence | 1385 | 1399 | £1859 (6252) | £2108 (6829) | 0.88 (0.42-1.83) | 0.633 |
|  | Presence | 1087 | 1020 | £1826 (6366) | £2710 (7587) | 0.67 (0.29-1.56) |  |
| Primary kidney diagnosis | Diabetic kidney disease | 727 | 677 | £2220 (6922) | £3260 (8255) | 0.68 (0.24-1.91) | 0.982 |
|  | Hypertension/reno-vascular | 553 | 572 | £1309 (5355) | £1382 (5679) | 0.95 (0.30-3.00) |  |
|  | Glomerular diseases | 670 | 636 | £2289 (6896) | £2888 (7765) | 0.79 (0.27-2.30) |  |
|  | Other or unknown | 522 | 534 | £1316 (5389) | £1639 (6077) | 0.80 (0.24-2.63) |  |
| 5-year kidney failure risk | <5% | 845 | 860 | £172 (2143) | £236 (2345) | 0.73 (0.30-1.79) | 0.496 ^a^ |
|  | ≥5%<20% | 814 | 765 | £856 (4442) | £1071 (4959) | 0.80 (0.31-2.04) |  |
|  | ≥20% | 813 | 794 | £4569 (9214) | £5895 (10370) | 0.78 (0.31-1.96) |  |

Rate ratios are estimated from negative binomial model adjusting for only treatment allocation, subgroup and their interaction as the shared parameter models and fully adjusted negative binomial models did not converge; 12 participants, 6 in each arm, entering post-trial follow-up but censored at the first day of post-trial follow-up were excluded from the rate analyses; ^a^ test for trend; ^b^ no participants from Japan joined the post-trial follow-up; ESKD, end stage kidney disease.

## Supplementary table S11. Effects of 2 years treatment with empagliflozin on **QALYs and healthcare costs**, overall and by subgroups at baseline

| Subgroup at baseline | | **Treatment effect (empagliflozin – placebo), Mean (95%CI)** | | | | | | | | | |
| --- | --- | --- | --- | --- | --- | --- | --- | --- | --- | --- | --- |
|  |  | **Over 2 years**  **(ACTIVE-TRIAL data)** | | | | | | **Two further years**  **(POST-TRIAL data)** | **Over 4 years**  **(ACTIVE-TRIAL + POST-TRIAL)** | | |
|  |  | **QALY** | **Each healthcare cost component (UK£)** | | | | **Total healthcare costs ^d^** | **Costs of ESKD management (UK£) ^c^** | **Costs of ESKD management (UK£) ^e^** | **Total healthcare costs (UK£) ^e^** |  |
| Name | **Level** |  | **Study medication ^a^** | **Hospital admission** | **Concomitant medication ^b^** | **ESKD management ^c^** |  |  |  |  |  |
| All | **-** | **0.012**  **(0.001, 0.022)** | **£826**  **(818, 834)** | **£-239**  **(-449, -29)** | **£-130**  **(-214, -47)** | **£-208**  **(-414, -2)** | **£249**  **(-75, 572)** | **£-842**  **(-1441, -242)** | **£-1050**  **(-1775, -324)** | **£-593**  **(-1384, 198)** |  |
| Age (year) | <60 | 0.009  (0.000, 0.018) | £850  (838, 861) | £-138  (-260, -15) | £-81  (-131, -31) | £-307  (-616, 1) | £324  (-27, 674) | £-1372  (-2383, -360) | £-1679  (-2882, -477) | £-1048  (-2279, 182) |  |
|  | 60-70 | 0.011  (0.001, 0.022) | £824  (813, 835) | £-243  (-457, -28) | £-146  (-239, -53) | £-175  (-355, 6) | £260  (-53, 574) | £-782  (-1385, -179) | £-957  (-1665, -248) | £-522  (-1301, 257) |  |
|  | ≥70 | 0.014  (0.001, 0.026) | £808  (796, 820) | £-324  (-610, -37) | £-162  (-268, -56) | £-148  (-307, 10) | £174  (-186, 533) | £-422  (-769, -75) | £-570  (-1014, -126) | £-248  (-820, 324) |  |
| Sex | Male | 0.012  (0.001, 0.023) | £826  (817, 836) | £-250  (-471, -29) | £-134  (-220, -48) | £-236  (-469, -2) | £207  (-147, 560) | £-902  (-1599, -205) | £-1138  (-1977, -298) | £-695  (-1600, 209) |  |
|  | Female | 0.011  (0.001, 0.020) | £826  (812, 840) | £-218  (-409, -27) | £-123  (-201, -45) | £-151  (-313, 10) | £334  (59, 608) | £-719  (-1266, -172) | £-870  (-1515, -225) | £-385  (-1090, 319) |  |
| Region | Europe | 0.012  (0.001, 0.022) | £826  (814, 838) | £-296  (-557, -35) | £-132  (-217, -47) | £-194  (-390, 2) | £204  (-153, 562) | £-671  (-1190, -153) | £-865  (-1509, -222) | £-467  (-1203, 269) |  |
|  | North America | 0.013  (0.001, 0.026) | £774  (755, 792) | £-194  (-377, -11) | £-205  (-339, -70) | £-76  (-220, 68) | £299  (24, 573) | £-243  (-532, 47) | £-319  (-702, 64) | £56  (-400, 512) |  |
|  | Japan | 0.010  (0.001, 0.019) | £860  (837, 882) | £-205  (-389, -21) | £-125  (-203, -46) | £-214  (-459, 32) | £316  (-25, 657) | -^f^ | -^f^ | - ^f^ |  |
|  | China and Malaysia | 0.011  (0.001, 0.022) | £828  (818, 839) | £-202  (-381, -24) | £-117  (-193, -42) | £-241  (-487, 5) | £268  (-63, 598) | £-1095  (-1929, -262) | £-1337  (-2318, -356) | £-828  (-1856, 200) |  |
| eGFR (mL/min/1.73m2) | <30 | 0.013  (0.001, 0.024) | £807  (795, 820) | £-297  (-561, -33) | £-141  (-233, -49) | £-474  (-944, -5) | £-105  (-673, 463) | £-1609  (-2857, -361) | £-2084  (-3620, -548) | £-1715  (-3311, -118) |  |
|  | 30-45 | 0.011  (0.001, 0.021) | £827  (817, 837) | £-227  (-427, -28) | £-137  (-225, -50) | £-76  (-160, 7) | £386  (140, 631) | £-462  (-822, -102) | £-538  (-948, -129) | £-76  (-570, 418) |  |
|  | ≥45 | 0.010  (0.001, 0.019) | £856  (844, 867) | £-170  (-322, -18) | £-99  (-160, -37) | £-35  (-83, 14) | £552  (373, 731) | £-197  (-365, -29) | £-232  (-430, -33) | £355  (84, 627) |  |
| uACR (mg/g) | <30 | 0.012  (0.001, 0.023) | £817  (802, 831) | £-259  (-491, -27) | £-162  (-266, -58) | £-48  (-107, 11) | £347  (76, 619) | £-78  (-158, 2) | £-126  (-247, -6) | £269  (-31, 569) |  |
|  | 30-300 | 0.012  (0.001, 0.023) | £823  (812, 834) | £-251  (-473, -30) | £-138  (-227, -49) | £-67  (-150, 16) | £366  (103, 629) | £-257  (-479, -36) | £-325  (-594, -56) | £108  (-279, 495) |  |
|  | 300-1000 | 0.011  (0.001, 0.020) | £840  (830, 850) | £-196  (-372, -20) | £-105  (-172, -38) | £-93  (-196, 9) | £445  (219, 672) | £-744  (-1321, -167) | £-838  (-1473, -203) | £-299  (-987, 390) |  |
|  | ≥1000 | 0.011  (0.001, 0.021) | £824  (812, 837) | £-253  (-473, -32) | £-122  (-200, -44) | £-584  (-1166, -3) | £-135  (-784, 515) | £-2161  (-3784, -537) | £-2744  (-4725, -763) | £-2295  (-4323, -266) |  |
| Diabetes status | Absence | 0.010  (0.001, 0.020) | £843  (833, 853) | £-172  (-323, -21) | £-69  (-112, -25) | £-192  (-386, 2) | £410  (146, 674) | £-790  (-1384, -195) | £-982  (-1698, -265) | £-380  (-1134, 375) |  |
|  | Presence | 0.013  (0.001, 0.025) | £807  (795, 819) | £-318  (-600, -37) | £-203  (-333, -72) | £-226  (-455, 3) | £60  (-347, 466) | £-908  (-1623, -192) | £-1134  (-1984, -284) | £-848  (-1794, 97) |  |
| Primary kidney diagnosis | Diabetic kidney disease | 0.013  (0.001, 0.025) | £806  (793, 818) | £-301  (-568, -34) | £-209  (-344, -74) | £-239  (-483, 6) | £57  (-351, 464) | £-1091  (-1953, -230) | £-1330  (-2332, -328) | £-1035  (-2120, 50) |  |
|  | Hypertension/reno-vascular | 0.012  (0.001, 0.022) | £825  (815, 835) | £-250  (-472, -28) | £-116  (-190, -41) | £-142  (-299, 16) | £317  (20, 615) | £-489  (-877, -102) | £-631  (-1115, -146) | £-172  (-736, 393) |  |
|  | Glomerular diseases | 0.010  (0.001, 0.019) | £851  (841, 861) | £-133  (-251, -15) | £-67  (-110, -25) | £-284  (-583, 15) | £366  (28, 704) | £-1104  (-1935, -274) | £-1388  (-2407, -370) | £-738  (-1784, 308) |  |
|  | Other or unknown | 0.011  (0.001, 0.021) | £828  (819, 838) | £-263  (-498, -28) | £-105  (-173, -38) | £-147  (-298, 4) | £313  (12, 614) | £-589  (-1069, -109) | £-736  (-1311, -161) | £-276  (-929, 376) |  |
| 5-year kidney failure risk | <5% | 0.011  (0.001, 0.021) | £839  (828, 849) | £-221  (-415, -26) | £-127  (-208, -47) | £-26  (-58, 7) | £465  (246, 685) | £-86  (-163, -9) | £-112  (-211, -12) | £379  (135, 623) |  |
|  | ≥5%<20% | 0.012  (0.001, 0.022) | £824  (815, 833) | £-248  (-469, -28) | £-140  (-229, -50) | £-73  (-153, 8) | £364  (99, 629) | £-379  (-682, -75) | £-451  (-802, -101) | £-15  (-472, 443) |  |
|  | ≥20% | 0.012  (0.001, 0.023) | £814  (802, 827) | £-252  (-473, -30) | £-125  (-206, -44) | £-534  (-1061, -6) | £-96  (-696, 504) | £-2064  (-3632, -497) | £-2598  (-4484, -712) | £-2160  (-4092, -228) |  |

Estimates are derived using the overall relative effect of allocation to empagliflozin from the estimated shared parameter models. ^a^ estimates are derived using the estimated negative binomial model in the empagliflozin arm adjusted for baseline variables specified in the minimization algorithm (age, sex, previous diabetes, eGFR, uACR, and region) and the overall relative effect of allocation to empagliflozin on time to death from the Cox proportional hazards model adjusted for treatment allocation and baseline variables specified in the minimization algorithm. ^b^ Selected concomitant medications included: antihypertensive treatments, antiplatelets, anticoagulants, diabetes medications, lipid lowering medications, drug for anemia, uric acid lowering medications, and Phosphate binders; ^c^ estimates are derived using the overall relative effect of allocation to empagliflozin on rate of costs from the estimated negative binomial model and on time to death from the estimated Cox proportional hazards model adjusted for treatment allocation and baseline variables specified in the minimization algorithm. ^d^ Total costs were derived by adding the separate cost components; ^e^ costs were derived by adding the costs over 2 years and ESKD costs over further two years;  ^f^ no participants from Japan entered post-trial follow-up. eGFR, estimated glomerular filtration rate; ESKD, end stage kidney disease; uACR, urine albumin-to-creatinine ratio

## Supplementary figure S3. Effect of 2 years treatment with empagliflozin on h**ealthcare costs over 2 years** (ACTIVE-TRIAL) and **over 4 years** (further 2 years POST-TRIAL) by key subgroups


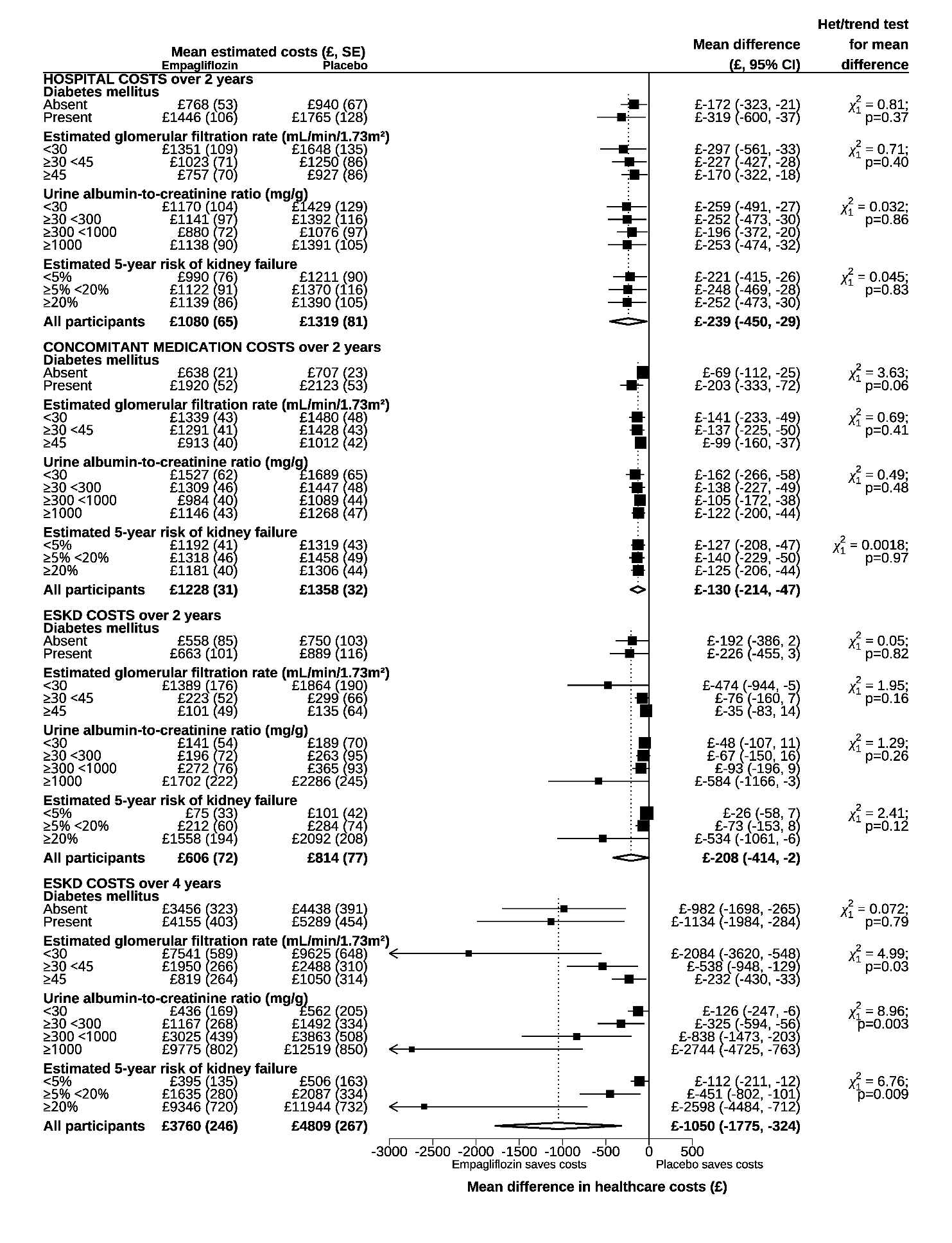


Concomitant medications included were antihypertensive treatments, antiplatelets, anticoagulants, diabetes medications, lipid lowering medications, drug for anemia, uric acid lowering medications, and Phosphate binders.

ESKD costs over 4 years were estimated based on rates of costs estimated over ACTIVE-TRIAL for the first 2 years and over POST-TRIAL for the latter 2 years and on time to death estimated over ENTIRE-TRIAL (both periods combined).

ESKD, End stage kidney disease;

## Supplementary figure S4. Probability of 2 years treatment with empagliflozin being cost-effective over 2 years (ACTIVE-TRIAL) and 4 years (further 2 years POST-TRIAL) by key subgroups


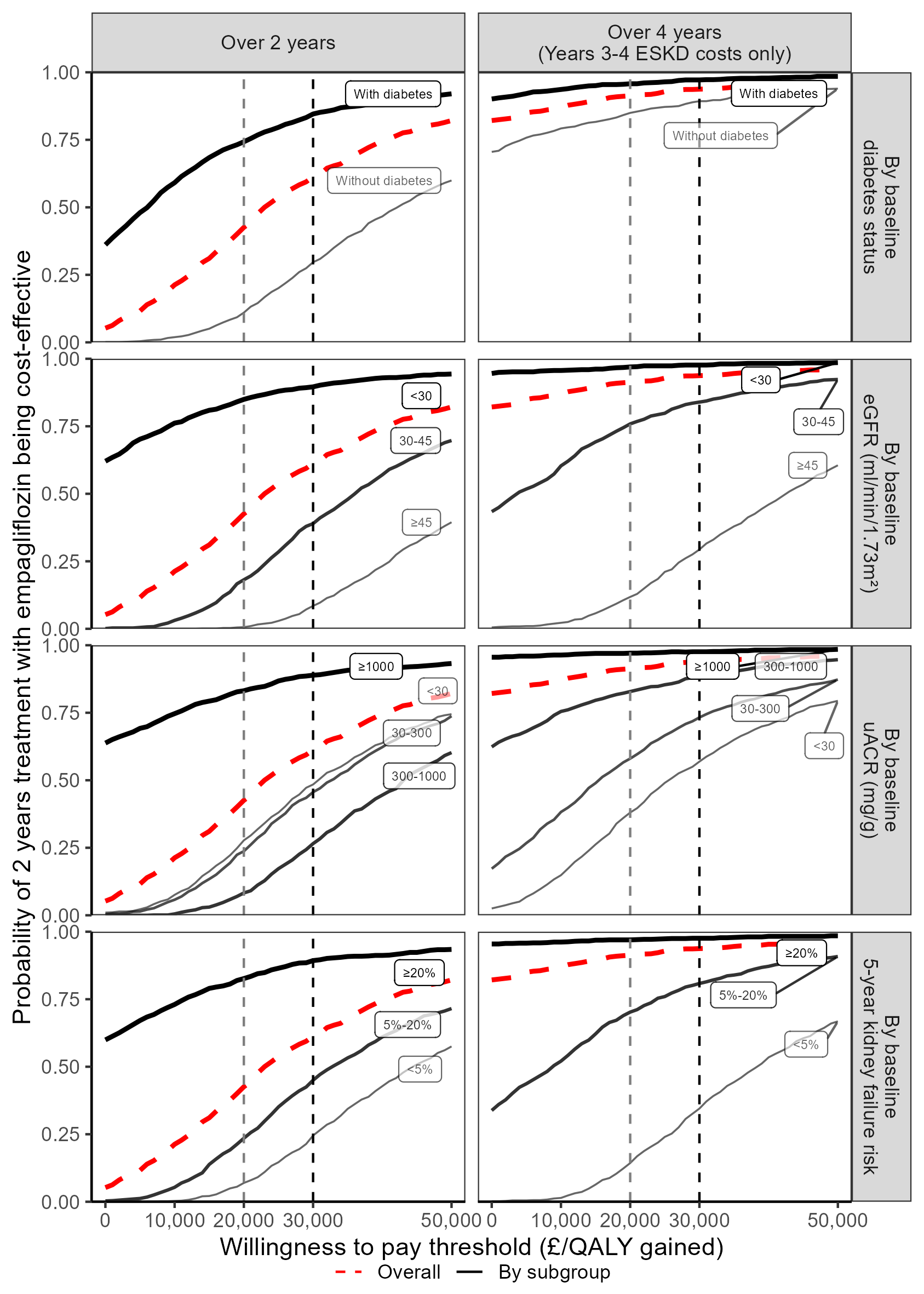


eGFR = estimated glomerular filtration rate. ESKD = end-stage kidney disease. QALY = quality-adjusted life year. uACR = urine albumin-to-creatinine ratio.

## Supplementary table S12. Effect of allocation to empagliflozin on **rate of admissions**, overall and by MedDRA SOC (Limited to active-trial data)

|  | # admissions/100 patient years of follow-up | | Effect of allocation to empagliflozin on rate of hospital admissions, Hazard ratio (95%CI) |
| --- | --- | --- | --- |
|  | Empagliflozin  (N = 3304) | Placebo  (N = 3305) |  |
| All | **24.8** | **29.3^a^** | **0.86 (0.78-0.95)** |
| By MedDRA SOC |  |  |  |
| Blood and lymphatic system disorders | 0.4 | 0.4 | 0.95 (0.55-1.62)^b^ |
| Cardiac disorders | 4.0 | 4.7 | 0.84 (0.68-1.04) |
| Congenital, familial and genetic disorders | 0.0 | 0.1 |  |
| Ear and labyrinth disorders | 0.1 | 0.0 | 2.34 (0.59-9.28)^b^ |
| Endocrine disorders | 0 | 0.0 |  |
| Eye disorders | 0.2 | 0.1 | 1.23 (0.51-3.00) |
| Gastrointestinal disorders | 1.4 | 1.7 | 0.83 (0.62-1.12) |
| General disorders and administration site conditions | 0.3 | 0.6 | 0.51 (0.30-0.89) |
| Hepatobiliary disorders | 0.5 | 0.4 | 1.29 (0.75-2.20) |
| Immune system disorders | 0.1 | 0 |  |
| Infections and infestations | 4.8 | 5.1 | 0.94 (0.78-1.12) |
| Injury, poisoning and procedural complications | 1.6 | 1.7 | 0.96 (0.72-1.28) |
| Investigations | 1.9 | 2.3 | 0.85 (0.66-1.09) |
| Metabolism and nutrition disorders | 1.6 | 1.9 | 0.85 (0.64-1.12) |
| Musculoskeletal and connective tissue disorders | 0.5 | 0.6 | 0.77 (0.48-1.24) |
| Neoplasms benign, malignant and unspecified (incl cysts and polyps) | 1.1 | 1.6 | 0.68 (0.49-0.95) |
| Nervous system disorders | 1.6 | 1.8 | 0.89 (0.67-1.18) |
| Pregnancy, puerperium and perinatal conditions | 0.0 | 0 |  |
| Psychiatric disorders | 0.1 | 0.3 | 0.47 (0.20-1.09)^b^ |
| Renal and urinary disorders | 2.6 | 3.4 | 0.79 (0.63-0.99) |
| Reproductive system and breast disorders | 0.1 | 0.1 |  |
| Respiratory, thoracic and mediastinal disorders | 0.8 | 0.9 | 0.95 (0.64-1.41) |
| Skin and subcutaneous tissue disorders | 0.2 | 0.2 | 0.92 (0.42-2.03)^b^ |
| Social circumstances | 0.0 | 0 |  |
| Surgical and medical procedures | 4.5 | 5.4 | 0.83 (0.69-0.99) |
| Vascular disorders | 0.7 | 1.0 | 0.75 (0.50-1.10) |

Hospitalization defined as the adverse event leading to hospitalization. Unless further specified, hazard ratio are estimated from shared parameter models adjusted for allocation to empagliflozin and baseline variables specified in the minimization algorithm (age, sex, previous diabetes, estimated glomerular filtration rate, uACR, and region); Hazard ratios not shown when fewer than 10 events occur.

^a^ different from the value (29.2) shown in table 2 in the main paper of the EMPA-KIDNEY trial^6^ because we identified and corrected an error of the admission date of one hospital episode in this analysis; ^b^ hazard ratio estimated based on shared parameter models only adjusting for treatment allocation as the fully adjusted shared parameter models did not converge. MedDRA SOC, Medical Dictionary for Regulatory Activities System Organ Class.

## Supplementary table S13. Effect of allocation to empagliflozin on **days in hospital** overall and by MedDRA SOC (Limited to active-trial data)

|  | Days in hospital per year of follow-up, Mean (SD) | | Effect of allocation to empagliflozin on  days in hospital , Rate ratio (95%CI) |
| --- | --- | --- | --- |
|  | Empagliflozin  (N = 3304) | Placebo  (N = 3305) |  |
| All | **2.58 (8.90)** | **2.91 (10.17)** | **0.82 (0.64-1.03)** |
| By MedDRA SOC |  |  |  |
| Blood and lymphatic system disorders | 0.02 (0.41) | 0.04 (0.64) | 0.67 (0.11-4.20)^a^ |
| Cardiac disorders | 0.39 (2.71) | 0.55 (4.72) | 0.89 (0.52-1.54) |
| Congenital, familial and genetic disorders | 0.00 (0.09) | 0.01 (0.59) |  |
| Ear and labyrinth disorders | 0.01 (0.19) | 0.00 (0.04) | 6.61 (0.87-50.41)^c^ |
| Endocrine disorders | 0 (0) | 0.00 (0.02) |  |
| Eye disorders | 0.01 (0.13) | 0.00 (0.10) | 1.64 (0.29-9.29)^b^ |
| Gastrointestinal disorders | 0.13 (1.87) | 0.13 (1.14) | 0.82 (0.34-1.98) |
| General disorders and administration site conditions | 0.01 (0.23) | 0.04 (0.69) | 0.36 (0.13-1.03)^a^ |
| Hepatobiliary disorders | 0.10 (3.32) | 0.04 (0.76) | 1.01 (0.11-9.64)^a^ |
| Immune system disorders | 0.00 (0.11) | 0 (0) |  |
| Infections and infestations | 0.59 (3.76) | 0.67 (4.18) | 0.96 (0.59-1.55) |
| Injury, poisoning and procedural complications | 0.22 (2.67) | 0.22 (3.60) | 1.06 (0.46-2.43) |
| Investigations | 0.12 (1.14) | 0.16 (1.47) | 0.86 (0.43-1.72) |
| Metabolism and nutrition disorders | 0.16 (1.53) | 0.15 (1.44) | 0.92 (0.42-2.01) |
| Musculoskeletal and connective tissue disorders | 0.05 (0.71) | 0.05 (0.64) | 0.82 (0.18-3.70)^a^ |
| Neoplasms benign, malignant and unspecified (incl cysts and polyps) | 0.19 (2.91) | 0.19 (2.42) | 0.81 (0.29-2.26) |
| Nervous system disorders | 0.21 (2.33) | 0.22 (2.38) | 0.94 (0.41-2.15) |
| Pregnancy, puerperium and perinatal conditions | 0.00 (0.01) | 0 (0) |  |
| Psychiatric disorders | 0.02 (0.65) | 0.07 (2.43) | 3.32 (0.70-15.66) |
| Renal and urinary disorders | 0.33 (2.36) | 0.40 (2.53) | 0.81 (0.43-1.52) |
| Reproductive system and breast disorders | 0.00 (0.12) | 0.00 (0.09) |  |
| Respiratory, thoracic and mediastinal disorders | 0.08 (1.24) | 0.09 (1.52) | 0.97 (0.28-3.39) |
| Skin and subcutaneous tissue disorders | 0.03 (1.04) | 0.01 (0.28) | 2.44 (0.46-12.94)^c^ |
| Social circumstances | 0.00 (0.09) | 0 (0) |  |
| Surgical and medical procedures | 0.36 (2.84) | 0.38 (2.77) | 0.83 (0.52-1.32) |
| Vascular disorders | 0.07 (1.00) | 0.06 (0.95) | 0.81 (0.24-2.76)^a^ |

Days in hospital per admission defined as the period of the adverse event leading to the admission. Unless further specified, rate ratios are estimated from shared parameter models adjusted for allocation to empagliflozin and baseline variables specified in the minimization algorithm (age, sex, previous diabetes, estimated glomerular filtration rate, uACR, and region). Rate ratios not shown when fewer than 10 events occur. ^a^ Rate ratio estimated based on shared parameter models adjusting for only treatment allocation; ^b^ Rate ratio estimated based on negative binomial models; ^c^ Rate ratio estimated based on negative binomial models adjusting for only treatment allocation. MedDRA SOC, Medical Dictionary for Regulatory Activities System Organ Class.

## Supplementary table S14. Effect of allocation to empagliflozin on **hospital costs** overall and by MedDRA SOC (Limited to active-trial data)

|  | Hospital costs per year of follow-up (UK£), Mean (SD) | | Effect of allocation to empagliflozin on hospital costs,  Rate ratio (95%CI) |
| --- | --- | --- | --- |
|  | Empagliflozin  (N = 3304) | Placebo  (N = 3305) |  |
| All | **£582 (1592)** | **£699 (2069)** | **0.81 (0.56-1.19)** |
| By MedDRA System Organ Class |  |  |  |
| Blood and lymphatic system disorders | £4 (59) | £6 (87) | 0.62 (0.03-13.27)^b^ |
| Cardiac disorders | £63 (316) | £83 (451) | 0.77 (0.42-1.42)^b^ |
| Congenital, familial and genetic disorders | £1 (37) | £1 (31) |  |
| Ear and labyrinth disorders | £1 (25) | £0 (17) | 2.77 (0.00-14923)^b^ |
| Endocrine disorders | £0 (0) | £0 (17) |  |
| Eye disorders | £2 (28) | £1 (28) | 1.16 (0.00-487)^b^ |
| Gastrointestinal disorders | £19 (182) | £23 (173) | 0.87 (0.31-2.42)^b^ |
| General disorders and administration site conditions | £2 (32) | £6 (70) | 0.18 (0.02-1.34)^b^ |
| Hepatobiliary disorders | £11 (215) | £7 (85) | 1.40 (0.19-10.19)^b^ |
| Immune system disorders | £1 (23) | £0 (0) |  |
| Infections and infestations | £97 (463) | £106 (615) | 0.96 (0.57-1.65)^b^ |
| Injury, poisoning and procedural complications | £34 (277) | £55 (1053) | 1.17 (0.40-3.46)^b^ |
| Investigations | £19 (124) | £20 (133) | 0.86 (0.33-2.26) |
| Metabolism and nutrition disorders | £19 (193) | £18 (117) | 0.90 (0.31-2.60) |
| Musculoskeletal and connective tissue disorders | £7 (94) | £9 (121) | 0.36 (0.04-2.95)^b^ |
| Neoplasms benign, malignant and unspecified (incl cysts and polyps) | £15 (161) | £29 (413) | 0.46 (0.14-1.52)^b^ |
| Nervous system disorders | £49 (544) | £61 (548) | 0.92 (0.30-2.85)^a^ |
| Pregnancy, puerperium and perinatal conditions | £0 (5) | £0 (0) |  |
| Psychiatric disorders | £4 (108) | £10 (213) | 0.06 (0.00-2.05)^b^ |
| Renal and urinary disorders | £45 (268) | £51 (264) | 0.81 (0.35-1.92) |
| Reproductive system and breast disorders | £1 (22) | £1 (27) |  |
| Respiratory, thoracic and mediastinal disorders | £11 (128) | £12 (122) | 0.94 (0.18-4.94) |
| Skin and subcutaneous tissue disorders | £3 (62) | £3 (49) | 0.50 (0.02-15.60)^b^ |
| Social circumstances | £2 (88) | £0 (0) |  |
| Surgical and medical procedures | £223 (1109) | £251 (1161) | 0.82 (0.41-1.65) |
| Vascular disorders | £10 (158) | £18 (413) | 0.57 (0.09-3.48)^a^ |

Rate ratios are estimated from shared parameter models adjusted for allocation to empagliflozin and baseline variables specified in the minimization algorithm (age, sex, previous diabetes, estimated glomerular filtration rate, uACR, and region). Rate ratios not shown when fewer than 10 events occur. ^a^ Rate ratios are estimated based on shared parameter models adjusting for only treatment allocation as the fully adjusted shared parameter models did not converge; ^b^ Rate ratio estimated based on negative binomial models as the shared parameter models did not converge. MedDRA SOC = Medical Dictionary for Regulatory Activities System Organ Class.

## Supplementary table S15. Effect of 2 years treatment with empagliflozin on **days of and costs of ESKD**, overall and by type of ESKD management

|  | EKSD days per year, Mean (SD) | | Rate ratio  (95%CI) | EKSD costs per year (UK£), Mean (SD) | | Rate ratio  (95%CI) |
| --- | --- | --- | --- | --- | --- | --- |
|  | **Empagliflozin** | **Placebo** |  | **Empagliflozin** | **Placebo** |  |
| Based on ACTIVE-TRIAL data | | | | | | |
| *All ESKD management* | *4.01 (27.28)* | *5.18 (28.63)* | *0.77 (0.41-1.47)* | *£313 (2142)* | *£422 (2341)* | *0.74 (0.33-1.70)* |
| *By type of ESKD management* |  |  |  |  |  |  |
| Dialysis | 3.49 (24.55) | 4.84 (27.71) | 0.72 (0.38-1.38) | £289 (2038) | £397 (2276) | 0.73 (0.31-1.69) |
| Transplant | 0.37 (7.69) | 0.31 (6.53) | 1.16 (0.10-13.40) | £25 (469) | £25 (490) | 1.01 (0.04-23.10) |
| Based on POST-TRIAL data | | | | | | |
| *All ESKD management* | *22 (87)* | *27 (97)* | *0.80 (0.51-1.24)* | *£1646 (6671)* | *£2134 (7703)* | *0.78 (0.45-1.36)* |
| *By type of ESKD management* |  |  |  |  |  |  |
| Dialysis | 18 (77) | 24 (91) | 0.75 (0.48-1.19) | £1490 (6375) | £1996 (7499) | 0.76 (0.42-1.35) |
| Transplant | 4 (35) | 3 (30) | 1.12 (0.36-3.53) | £156 (1333) | £138 (1249) | 1.13 (0.28-4.62) |

Rate ratios are estimated from negative binomial models adjusting for only treatment allocation as the shared parameter models and fully adjusted negative binomial models did not converge. ESKD, end stage kidney disease.

## Supplementary figure S5. Effect of 2 years treatment with empagliflozin on **total healthcare costs over 4 years** (Years 3-4 ESKD costs only) in categories of CKD patients, at different empagliflozin cost


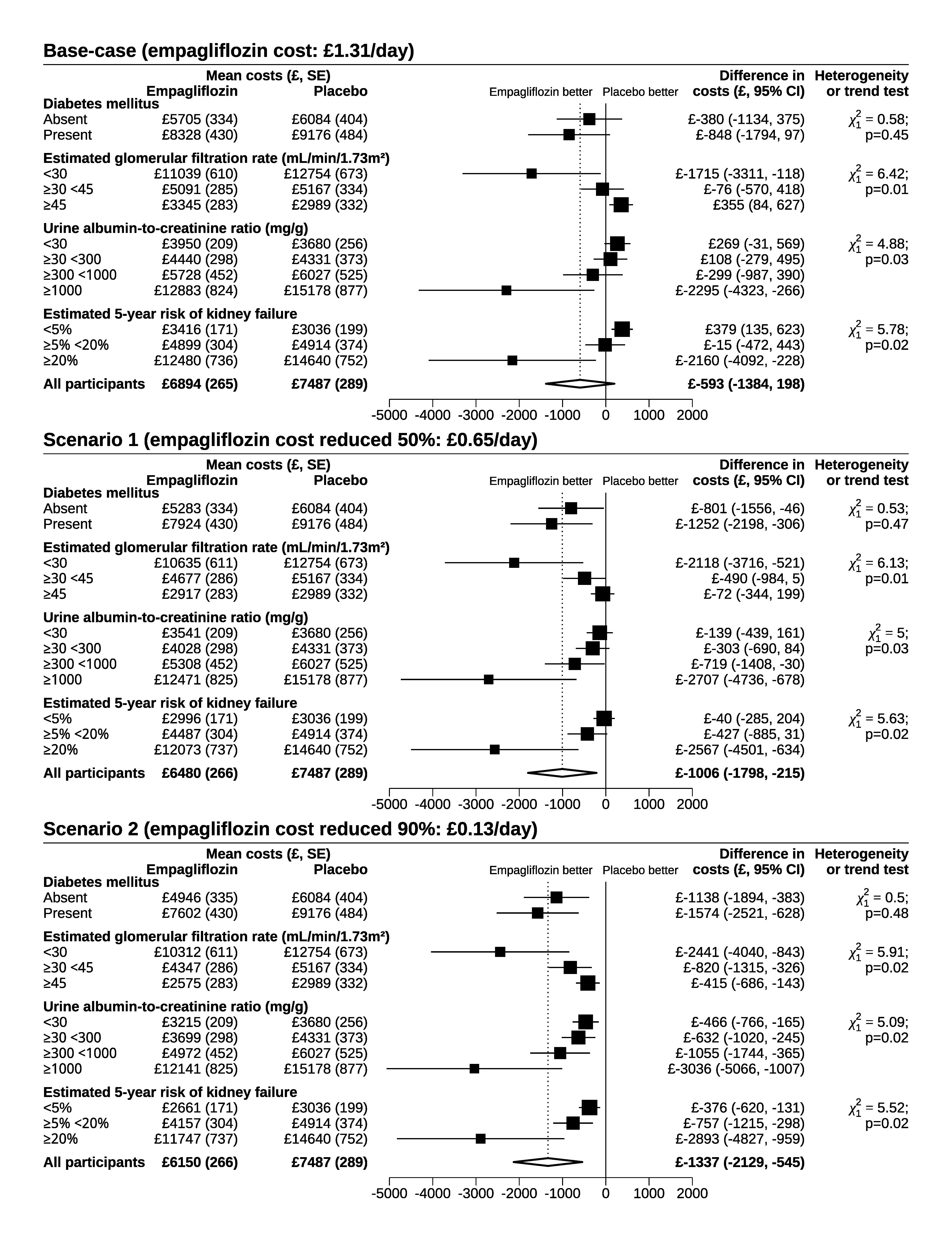


Total healthcare costs over 4 years included costs of empagliflozin, hospital admission and concomitant medications and ESKD costs over 4 years. Concomitant medications included were antihypertensive treatments, antiplatelets, anticoagulants, diabetes medications, lipid lowering medications, drug for anemia, uric acid lowering medications, and Phosphate binders) over 2 years;

ESKD = end stage kidney disease.

## Supplementary figure S6. Probability of 2 years treatment with empagliflozin being cost-effective over 4 years (2 years ACTIVE-TRIAL + further 2 years POST-TRIAL) in categories of CKD patients, at different empagliflozin cost


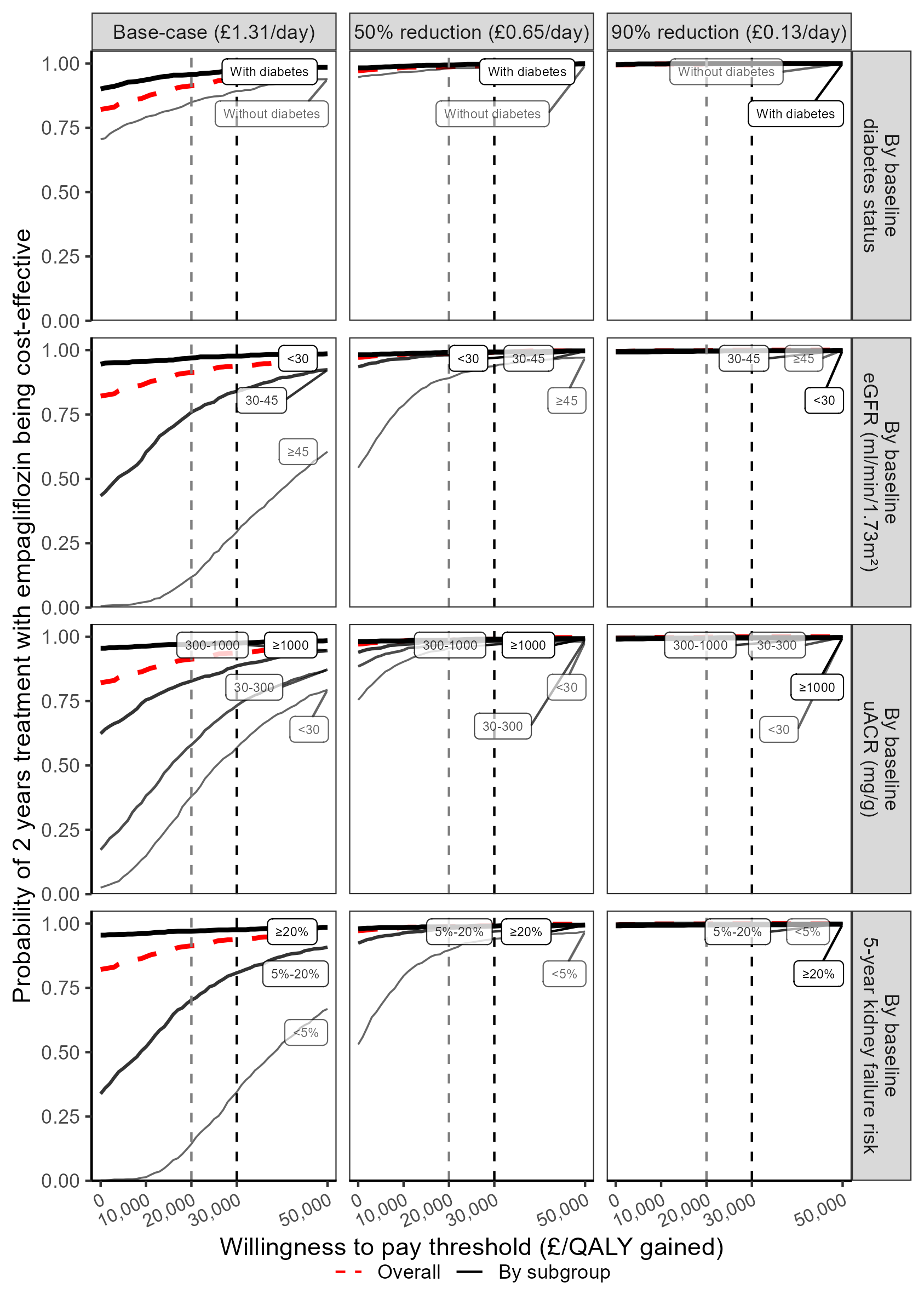


eGFR = estimated glomerular filtration rate. QALY = quality-adjusted life year. uACR = urine albumin-to-creatinine ratio.

## Supplementary References

1. NHS Improvement. National cost collection guidance 2021/22. 2023. Available from <https://www.england.nhs.uk/costing-in-the-nhs/national-cost-collection/> (accessed on 2024/11/01).

2. Personal Social Services Research Unit. Unit Costs of Health and Social Care 2023 Manual. 12.1. Inflation indices. 2024. Available from: <https://doi.org/10.22024/UniKent/01.02.105685> (accessed on 2024/11/01).

3. UK Kidney Association. UK Renal Registry 24th Annual Report (Data to 31/12/2020). 2022. Available from: <https://ukkidney.org/sites/renal.org/files/24th_UKRR_ANNUAL_REPORT_BOOK%20version%203.pdf> (accessed on 2024/11/01).

4. NHS Business Services Authority. Prescription Cost Analysis - England - 2022/23. 2023. Available from: <https://www.nhsbsa.nhs.uk/statistical-collections/prescription-cost-analysis-england/prescription-cost-analysis-england-202223> (accessed on 2024/11/01).

5. The EMPA-KIDNEY Collaborative Group. Effects of empagliflozin on progression of chronic kidney disease: a prespecified secondary analysis from the empa-kidney trial. *Lancet Diabetes Endocrinol* 2024; **12**(1): 39-50.

6. The EMPA-KIDNEY Collaborative Group. Empagliflozin in Patients with Chronic Kidney Disease. *N Engl J Med* 2023; **388**(2): 117-27.
